# A positive feedback loop: RAD18-YAP-TGF-β between triple-negative breast cancer and macrophages regulates cancer stemness and progression

**DOI:** 10.1038/s41420-022-00968-9

**Published:** 2022-04-12

**Authors:** Xueqi Yan, Yaozhou He, Shikun Yang, Tianyu Zeng, Yijia Hua, Shengnan Bao, Fan Yang, Ningjun Duan, Chunxiao Sun, Yan Liang, Ziyi Fu, Xiang Huang, Wei Li, Yongmei Yin

**Affiliations:** 1 Department of Oncology, the First Affiliated Hospital of Nanjing Medical University, 210029 Nanjing, China; 2 Hepatobiliary/Liver Transplantation Center, The First Affiliated Hospital of Nanjing Medical University; Key Laboratory of Liver Transplantation, Chinese Academy of Medical Sciences, 210029 Nanjing, China; 3 Jiangsu Key Lab of Cancer Biomarkers, Prevention and Treatment, Collaborative Innovation Center for Personalized Cancer Medicine, Nanjing Medical University, 211166 Nanjing, China

**Keywords:** Breast cancer, Cancer microenvironment

## Abstract

As a key regulator of the DNA translesion synthesis (TLS) pathway, RAD18 is error-prone and contributes to the accumulation of DNA mutations. Our previous study showed that it plays an essential role in the progression of multiple tumors. However, the mechanism through which RAD18 influences triple-negative breast cancer (TNBC), especially the interaction between tumor cells and the tumor microenvironment, remains elusive. In this study, we showed that RAD18 expression is markedly higher in patients with high T stage TNBC and inversely correlated with prognosis. High expression of RAD18 facilitated a highly stem-cell phenotype through the Hippo/YAP pathway, which supports the proliferation of TNBC. In addition, the cytokine byproduct TGF-β activates macrophages to have an M2-like tumor-associated macrophage (TAM) phenotype. Reciprocally, TGF-β from TAMs activated RAD18 in TNBC to enhance tumor stemness, forming a positive feedback loop. Inhibition of YAP or TGF-β breaks this loop and suppresses cancer stemness and proliferation In nude mice, RAD18 promoted subcutaneous transplanted tumor growth and M2-type TAM recruitment. Collectively, the RAD18-YAP-TGF-β loop is essential for the promotion of the stemness phenotype by TNBC and could be a potential therapeutic target for TNBC.

## Introduction

Breast cancer surpass lung cancer, being the most commonly diagnosed cancer worldwide [1]. Triple-negative breast cancer (TNBC), which lacks the expression of the estrogen receptor (ER), progesterone receptor (PR), and human epidermal growth factor receptor 2 (HER2), accounts for 10–15% of breast cancers [2]. TNBC is considered the most aggressive subtype of breast cancer and is characterized by early recurrence, high incidence of visceral metastasis, and short survival time [3]. Due to its high heterogeneity and lack of effective molecular targets, the efficacies of current treatments are still limited. Therefore, novel effective strategies for TNBC treatment are urgently required.

The E3 ubiquitin ligase RAD18 is well known for the maintenance of genome stability and cell survival through many DNA damage response (DDR) pathways such as translesion DNA synthesis (TLS) and homologous recombination repair (HRR) [4, 5]. Additionally, RAD18 functions beyond DNA repair and may regulate many other key biological processes, such as chromatin strengthening, cell survival/death, stemness, and differentiation. For example, RAD18 plays a vital role in regulating the DNA stability of embryonic stem cells and cellular homeostasis in highly prolific cells [6]. In response to endogenous and exogenous insults, malignant cells mostly have an intensive DNA repair capacity that allows them to proliferate and survive. Previous studies have shown that RAD18 is highly expressed in multiple human cancers and that elevated RAD18 expression is related to poor outcome [7]. For example, high expression of RAD18 regulates melanoma cell proliferation [8]. RAD18 promotes the migration and invasion of esophageal squamous cell cancer via the JNK-MMPs pathway [9]. RAD18^high^ predicts the poor response to preoperative concurrent chemoradiotherapy in patients with locally advanced rectal cancer [10]. Nevertheless, the role of RAD18 in breast cancer has not been well explored.

Tumor microenvironment (TME) remodeling is a critical process in primary tumor progression. TAMs are the major inflammatory cells in the TME, and are closely related to poor outcomes in several types of tumors [11]. TAMs are affected by activated signals and exhibit phenotypes with different functions. The most common subtypes include M1-like (pro-inflammatory) and M2-like (anti-inflammatory) [12]. The strategy of converting M2- into M1-like macrophages has shown promising results in cancer treatment. An anti-MARCO monoclonal antibody inhibits tumor activity and reprograms TAM populations into M1-like macrophages [13]. Meanwhile, DDR in tumors can also regulate the polarization of TAMs [14, 15]. For example, ATR is a key factor in single-strand break (SSB) repair, in which mutations stimulate M2 macrophage accumulation associated with tumor growth and invasion [16]. However, the connection between RAD18 and its immune-related function is mostly unknown, although Bachl et al. speculated its contribution to somatic hypermutation and Ig diversification by promoting proliferating cell nuclear antigen ubiquitination [17].

In this study, we showed that RAD18 was highly expressed in TNBC and inversely related to patient prognosis. RAD18 overexpression increased CD44+/CD24-BCSCs through the Hippo/YAP pathway, thus promoting breast cancer progression. Furthermore, we showed that the effects of RAD18 on TAM polarization and the underlying mechanisms were related to the TGF-β. Overall, this study highlights the potential regulatory pathways of RAD18-mediated tumor progression, thus improving RAD18-based prognosis prediction and anticancer therapy.

## Material and methods

### Patients and samples

A total of 40 human TNBC tissues and 10 adjacent tissues were obtained from the First Affiliated Hospital of Nanjing Medical University. The histopathology was confirmed by the pathology department of First Affiliated Hospital of Nanjing Medical University. This study was approved by the Institutional Ethics Committee of the Nanjing Medical University. The written informed consents were signed for each patient.

### Cell lines and cell culture

Human breast epithelial cell line MCF10A, human breast cancer cell lines MCF-7, T-47D, SKBR3, HCC1954, HCC1806, MDA-MB-231, and the human monocytic cell line THP-1 were all bought from American Type Culture Collection (ATCC, VA, US). All cell lines were recently authenticated by short tandem repeat profiling and tested for mycoplasma contamination. MCF10A was cultured in DMEM/F12 (Gibco, NY, US) medium supplemented with insulin, hydrocortisone, EGF, 5% FBS (Gibco, NY, US), and 1% penicillin/streptomycin (Gibco, NY, US). MDA-MB-231 were cultured in Dulbecco’s modified Eagle’s medium (DMEM, Hyclone, UT, US) with 10% FBS and 1% penicillin/streptomycin. SKBR3 were cultured in McCoy’s 5a Medium with10% FBS and 1% penicillin/streptomycin. Other cells were all cultured in RPMI 1640 medium (Hyclone, UT, US) with 10% FBS and 1% penicillin/streptomycin. A humidified atmosphere of 5% CO2 and 37 °C were maintained for cell culture.

### Reagents and antibodies

Anti-RAD18 (MA532284) was from Invitrogen. StemLight™ Pluripotency Transcription Factor Antibody Kit (9093T) and Hippo Signaling antibody sampler kit (8579T) was from Cell Signaling Technology. Anti-CD44 (15675-1-AP) was from Proteintech. PMA (Sigma-Aldrich, MO, US) was dissolved in DMSO and stored at −20 °C. Anti-CD68 (25747-1-AP), anti-CD86 (13395-1-AP) and anti-CD163 (16646-1-AP) for immunofluorescence (IF) staining were purchased from Proteintech. APC Anti-CD86 (65165) and PE anti-CD163 (12-1639-41) were bought separately from Proteintech and eBioscience. The secondary antibody were bought from CWBIO (Beijing, China). Anti-human TGF-β1 antibody (21898-1-AP) and neutralizing antibodies (69012-1-Ig) were purchased from Proteintech.

### TNBC cell transfections

RAD18 short hairpin RNA (shRNA) and negative control shRNA (Hanbio, Shanghai, China). The RAD18 shRNA target sequences are 5′-GCUCUCUGAUCGUGAUUUATT-3′ and 5′-UAAAUCACGAUCAGAGAGCTT-3′. MDA-MB-231 and HCC1806 cells (1 × 10^6^) were seeded into six-well culture plates and transfected with RAD18 shRNA or negative control shRNA. The siRNA targeting YAP and stable overexpression YAP and control cell lines were constructed. The transfection efficiency was detected by qRT-PCR or western blot (WB) after incubation for 48 h.

### CCK‐8 proliferation assay

Cells (2000/100 μL) were seeded into 96‐well culture plates each well and incubated for 24, 48, 72, 96, 120 h. Then the cultivation medium were substituted by fresh medium with 10% CCK‐8 (Beyotime Biotechnology, Shanghai, China) per well for 1 h. The absorbance was detected at 450 nm by a microplate reader (Thermo, USA).

### Colony formation assay

Cells were cultivated in six‐well culture plates at 1000 cells per well, respectively. The cell colonies were dyed with crystal violet reagent (Beyotime Biotechnology) after 2 weeks. The colonies containing more than 50 cells were counted. Cell survival histogram was fitted by GraphPad Prism 7 (GraphPad Software, Inc La Jolla, USA).

### Tumor sphere assay

Cells were cultured in ultra-low attachment six-well-plates with MammoCult™ Human Medium Kit (#05620, Stemcell Technologies, Canada) supplemented with Hydrocortisone Stock Solution (#07925) and Heparin Solution (Catalog #07980). After culture for 7–10 days, the tumor spheres containing more than 50 cells were counted.

### Macrophage generation and differentiation

THP-1 monocytes were treated with 200 nM phorbol 12-myristate 13-acetate (PMA, P8139; Sigma-Aldrich, St. Louis, MO, USA) for 48 h to differentiate to macrophages. The macrophages (1 × 106 cells/well) were co-cultured with MDA-MB-231 shNC or MDA-MB-231 shRAD18 cells (2 × 105 cells/well) for 72 h.

### Cytokine detection

The conditioned medium (CM) was harvested and filtered to detect secreted cytokines. To detect the secreted cytokines from particular cell, the cell should be washed with PBS three times after terminating co-cultivation and cultivated with serum-free medium for another 24 h. Then the supernatant was harvested. All the medium should be centrifuged for 20 min at 1000×*g* at 4 °C before use. The primary macrophage (M0) functioned as control group.

### RNA isolation and quantitative real-time PCR

Total RNA was extracted from MDA-MB-231, HCC1806 cells, and macrophages using the TRIZOL extraction kit (Invitrogen), and then reverse transcripted by TAKARA reverse transcriptase (RR047A, Japan). mRNA levels were determined by qPCR using particular primers and SYBR green PCR kit (RR820A, TAKARA, Japan). The primers for human RAD18, YAP, IL1B, IL6, TNF, TGFB1, IL10, and VEGFA were as follows: RAD18, Forward: 5′-GTCCTTTCATCCTCTACTCTCGT-3′, Reverse: 5′- TAGCCTTCTCTATGTTGTCTATCCC-3′; YAP, Forward: 5′-TAGCCCTGCGTAGCCAGTTA-3′, Reverse: 5′-TCATGCTTAGTCCACTGTCTGT-3′; CD44, Forward: 5′-CTGCCGCTTTGCAGGTGTA-3′, Reverse: 5′-CATTGTGGGCAAGGTGCTATT-3′; OCT4, Forward: 5′-CTTGAATCCCGAATGGAAAGGG-3′, Reverse: 5′-GTGTATATCCCAGGGTGATCCTC-3′; SOX2, Forward: 5′-GCCGAGTGGAAACTTTTGTCG-3′, Reverse: 5′-GGCAGCGTGTACTTATCCTTCT-3′; NANOG, Forward: 5′-CCCCAGCCTTTACTCTTCCTA-3′, Reverse: 5′-CCAGGTTGAATTGTTCCAGGTC-3′; IL1B, Forward: 5′-ATGATGGCTTATTACAGTGGCAA-3′, Reverse: 5′-GTCGGAGATTCGTAGCTGGA-3′; IL6, Forward: 5′-ACTCACCTCTTCAGAACGAATTG-3′, Reverse: 5′-CCATCTTTGGAAGGTTCAGGTTG-3′; TNF, Forward: 5′-GAGGCCAAGCCCTGGTATG-3′, Reverse: 5′-CGGGCCGATTGATCTCAGC-3′; TGFB1, Forward: 5′-CTAATGGTGGAAACCCACAACG-3′, Reverse: 5′-TATCGCCAGGAATTGTTGCTG-3′; IL10, Forward: 5′-TCAAGGCGCATGTGAACTCC-3′, Reverse: 5′-GATGTCAAACTCACTCATGGCT-3′; VEGFA, Forward: 5′-AGGGCAGAATCATCACGAAGT-3′, Reverse: 5′-AGGGTCTCGATTGGATGGCA-3′. The relative mRNA levels were determined compared to the GAPDH (Forward: 5′-GGTATGACAACGAATTTGGC-3′, Reverse: 5′-GAGCACAGGGTACTTTATTG-3′) control.

### Nuclear and cytoplasmic extraction

The cytoplasmic and nuclear extracts were separated by Membrane and Cytosol Protein Extraction Kit (P0033, Beyotime, Jiangsu, China). Histone H3 (1:1000; #9715, CST) and GAPDH (1:1000; ab181602, Abcam) were respectively used as nuclear and cytoplasmic protein reference.

### Western blot

Cells were harvested and lysed in ice-cold buffer (Beyotime, Jiangsu, China). WB was performed following manufacturer’s protocol. Primary antibodies against RAD18, CD44, Nanog, SOX2, OCT-4, MST1, MST2, LATS1, p-YAP S127, YAP, Histone H3,CD68, CD86, CD163, NF-κBp65, NF-κBp-p65, c-Jun, PPAR-δ, JNK, and p-JNK were diluted as 1:1000 in 5% bovine serum albumin, GAPDH (1:1000; ab181602, Abcam) and Histone H3 (1:1000; #9715, CST) was used as the internal control.

### Immunofluorescence

Cells were seeded on glass coverslips and cultured for time based on the objective of the experiments. Then they were double stained and DAPI was used as a nuclear counterstain. The images were taken by a confocal laser scanning microscope.

### Bioinformation analysis

Expression level of RAD18 in cancer were analyzed on TCGA and GSE65194 database. Kaplan–Meier plots were accessed online. The factors inducing macrophage polarization that related to RAD18 were searched based on the TIMER software [18]. The cytokines with significant different were chosen for subsequent enzyme linked immunosorbent assay (ELISA) verification.

### Enzyme-linked immunosorbent assay

ELISA detection of TGF-β in the culture supernatant was followed the kit instructions (E0124h, EIAab, Wuhan, China).

### Flow cytometry analysis

Flow cytometry analysis was to evaluate the cell apoptosis, cell stemness, and macrophage polarization. Apoptosis was assessed by the Annexin V-7-AAD Kit (Beyotime, Jiangsu, China). Cells were centrifuged at 100×*g* for 5 min and washed twice with PBS, then resuspended with binding buffer. Annexin V-FITC (5 µL) and 7-AAD (5 µL) were added for 20 min in the dark at 25 °C and analyzed using a FACS Calibur flow cytometer (Becton Dickinson, Mountain View, CA, USA). The analysis steps of cell stemness were the same as the former cell experiments. The cells were labeled with CD44 (5 µL) and CD24 (5 µL) for 20 min and analyzed using the FACS. The macrophages were trypsinized and resuspended into the flow cytometry tubes. 3% bovine serum albumin (Sigma) was used to block for 25 min. Then 5 μl extracellular antibody APC anti-human CD86 and PE anti-human CD163 were added in cells for 30 min and then detected by flow cytometry.

### Immunohistochemistry

The 50 clinical specimens and the tumors of mice were detected by immunohistochemistry. In all, 4 μm formalin-fixed and paraffin-embedded sections were de-paraffinized with xylene twice and rehydrated in graded 100%, 90%, 80%, and 70% alcohol solution, and the antigens were retrieved with Tris-EDTA buffer for 3–5 min at 100 °C. The slides were peroxidase blocked with 3% hydrogen peroxide solution for 10 min and then blocked using 5% bovine serum albumin (Sigma) for 30 min. Slides were incubated with primary antibody against RAD18, CD44, YAP, CD163, and TGF-β overnight. Then the slides were detected by the ChemMate EnVision kit (Dako, Carpinteria, CA, USA). The investigators were blinded to the groups allocation during the experiments.

### Xenograft tumor assay

Female BALB/c nude mice (4–5 weeks old) were fed with sterilized chow and water, under pathogen-free conditions. Mice were randomly divided into 4 groups (*n* = 5/group), shNC-MDA-MB-231, shRAD18-MDA-MB-231, shNC-HCC1806, shRAD18-HCC1806. A total of 6 × 10^6^ cells for each group in 100 μl PBS were injected subcutaneously into the right axilla of the BALB/c nude mice, respectively. Tumor size was measured every 4 days by calipers and tumor volumes were calculated by the formula (maximum diameter × minimum diameter^2^)/2. Tumors were collected for immunohistochemistry analysis. The animal experiments were approved by the Scientific and Ethical Committee of the Institute of Nanjing Medical University.

### Statistical analysis

Data were presented as mean ± SD from more than three independent experiments. Student’s *t* test was used for pairwise comparisons between groups, and one-way ANOVA was used for experiments including three or more groups. Chi-squared test was used to clinical samples analysis. All experiments were performed independently at least three times. The statistical analysis were all performed by GraphPad Prism software or SPSS software. *P* < 0.05 was considered statistical significance.

## Results

### RAD18 is highly expressed in TNBC and positively correlates with poor prognosis

**Fig. 1:**
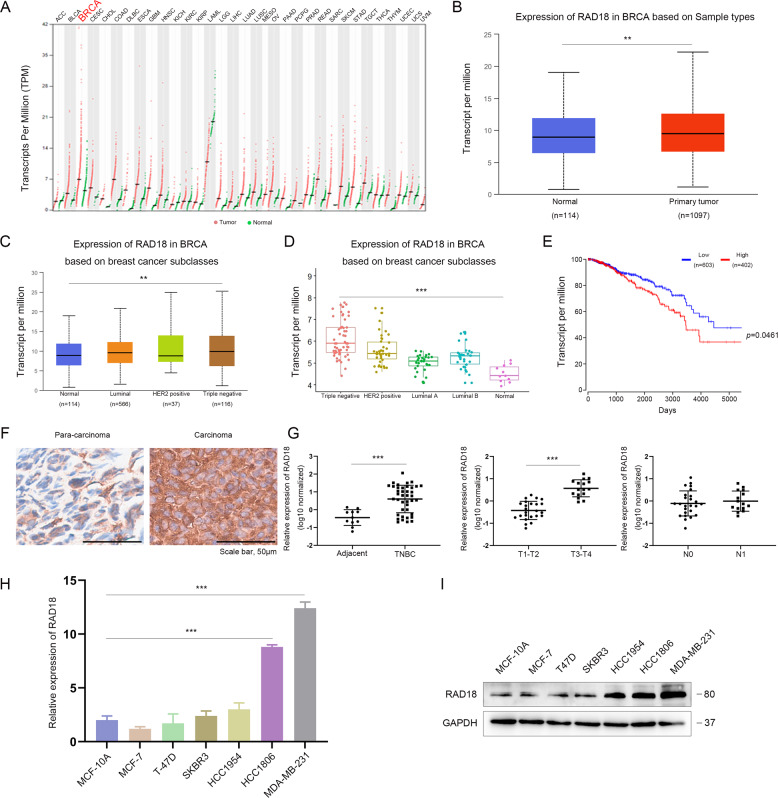
RAD18 is high expression in TNBC and positively correlates with poor prognosis. RAD18 expression is frequently elevated in TNBC and associated with poor prognosis of TNBC patients. **A** RAD18 was relatively high expressed in breast cancer than any other cancer types. **B** The expression level of RAD18 was higher in TNBC tissues (*n* = 1097) compared to the surrounding normal tissues (*n* = 114). **C**, **D** RAD18 was especially high expression in TNBC according to TCGA database and GSE65194. **E** Kaplan–Meier survival analysis revealed that high RAD18 expression associated with a shorter overall survival (OS) compared to low RAD18 expression in TNBC patients (*p* = 0.0461). **F** Immunohistochemical analysis of RAD18 in 40 TNBC tissues samples (400×). **G** The expression of RAD18 was significantly higher in TNBC tissues (*n* = 40) than adjacent normal tissues (*n* = 10). The RAD18 expression level correlated with T tumor stages (*n*_T1-T2=24,_ *n*_T1-T2=16_), but not with lymph node metastasis (*n*_N0=25,_ *n*_N1=15_). **H**, **I** qRT-PCR and western blot showed that MDA-MB-231 and HCC-1806, as the TNBC cell lines, high expressed RAD18 compared to normal mammary epithelial cells (MCF-10A) and other breast cancer subtypes cell lines. *p < 0.05, **p < 0.01, ***p < 0.001.

To explore the role of RAD18 in breast cancer, we detected the expression level of RAD18 in multiple cancer subtypes from The Cancer Genome Atlas (TCGA) breast cancer database. RAD18 was especially highly expressed in breast cancer (Fig. 1A), and the average expression level of RAD18 was significantly higher in tumor samples than in normal samples (*p* < 0.01, Fig. 1B). Specifically, TNBC, as the most aggressive subtype of breast cancer, showed the highest expression of RAD18 in both the TCGA database (*p* < 0.01, Fig. 1C) and GSE65194 (*p* < 0.001, Fig. 1D). RAD18 expression in 1005 BRCA samples was analyzed in combination with Kaplan–Meier survival, and high RAD18 expression was closely associated with shorter overall survival (*p* < 0.05, Fig. 1E).

To confirm this conclusion, RAD18 expression was detected in another cohort of 40 TNBC samples and 10 normal matched adjacent samples. The results showed that RAD18 was overexpressed in TNBC tissues and localized mainly in the cytoplasm (Fig. 1F). Besides, TNBC patients with T3-T4 stages showed significantly higher expression of RAD18 than those with T1-T2 stages (*p* < 0.001, Fig. 1G), whereas RAD18 levels were not correlated with N staging (*p* > 0.05, Fig. 1G). qRT-PCR and western blotting showed consistent results in breast cancer cell lines and human normal mammary epithelial cells MCF-10A (Fig. 1H–I).

Based on these results, we concluded that RAD18 is highly expressed in TNBC and might play a vital role in the development and progression of TNBC.

### RAD18 promotes TNBC cell proliferation and reduces apoptosis in vitro

**Fig. 2:**
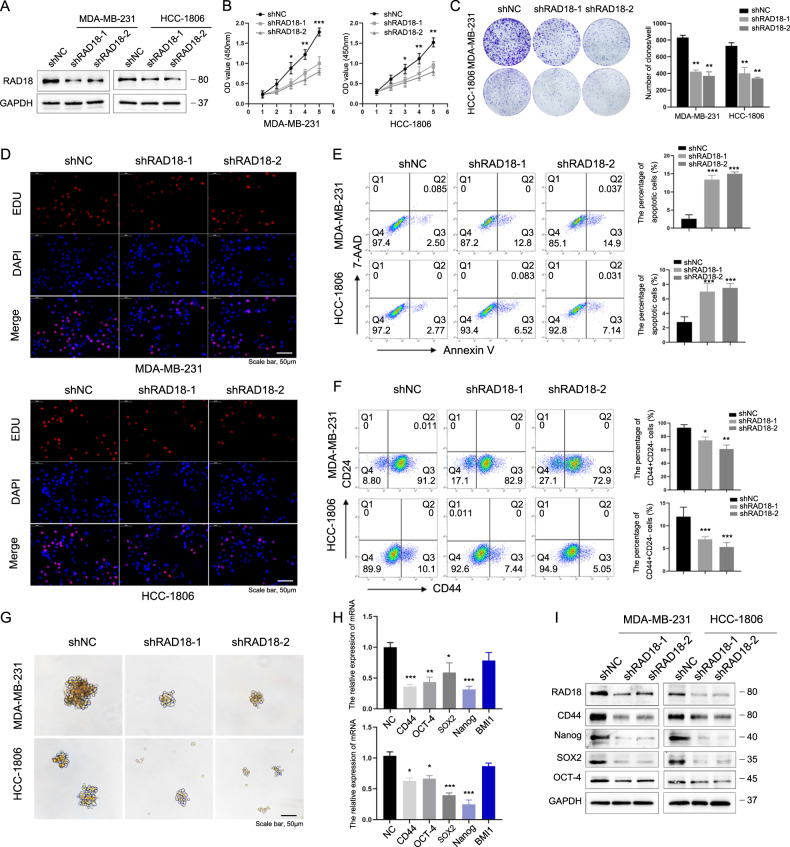
RAD18 promotes TNBC cell proliferation and maintains the stemness of TNBC in vitro. RAD18 increases TNBC cell proliferation and maintains the CSC stemness in vitro. **A** RAD18 protein levels in TNBC cells transfected with RAD18 shRNA lentivirus were determined by western blotting. **B** Cells were seeded in a 96-well plate and CCK-8 assay was used to detect cell proliferation at indicated hours (24, 48, 72, 96, and 120 h). **C** colony formation assay was performed to examine cell proliferation. Cells were seeded in six-well plates at a density of 2000 cells per well and cultivated for 2w. **D** An EdU assay was performed to determine the proliferation of different groups of TNBC cells. **E** Cell apoptosis in shNC and shRAD18 TNBC cells were assessed by Annexin V/7‐AAD flow cytometric analysis. **F** Representative flow cytometric analysis of CD44+/CD24− BCSC population percentages in shNC and shRAD18 TNBC cells. **G** The tumor sphere formation in TNBC cells treated as indicated (200×). The quantification of mammospheres were counted using a microscope with size ≥50 µm. **H**, **I** mRNA and protein levels of stemness-related signature genes (CD44, OCT4, SOX2, Nanog). GAPDH was analyzed as a loading control. *p < 0.05, **p < 0.01, ***p < 0.001. All experiments were performed independently at least three times.

To investigate how RAD18 affects TNBC progression, RAD18 shRNA was transfected into adopted TNBC cells, MDA-MB-231 and HCC1806. The silencing efficiency of RAD18 was verified by western blotting (Fig. 2A). The proliferative ability of these cell models was assessed by CCK-8 and colony formation assays. RAD18 knockdown dramatically suppressed the proliferation of TNBC cells (Fig. 2B, C). Concordant with these results, 5-ethynyl-2-deoxyuridine (EdU) assays indicated that knockdown of RAD18 in TNBC cells significantly reduced the rates of EdU incorporation (Fig. 2D). Furthermore, the cell apoptotic rates in the shRAD18 group were markedly increased compared to shNC group (Fig. 2E).

In conclusion, these data show that RAD18 promotes proliferation and reduces apoptosis in TNBC cells.

### RAD18 maintains the CSCs stemness of TNBC in vitro

Tumors often exhibit a hierarchical organization, whose apex is occupied by a subpopulation of cancer cells known as cancer stem cells (CSCs) because of their ability to self-renew and generate progeny with different degrees of differentiation. CSCs have demonstrated tolerated constitutive replication stress and survived [19]. Therefore, we suspected that the DDR-related protein RAD18-boosted proliferation and reduced apoptosis of TNBC cells was associated with CSC enrichment.

**Fig. 3:**
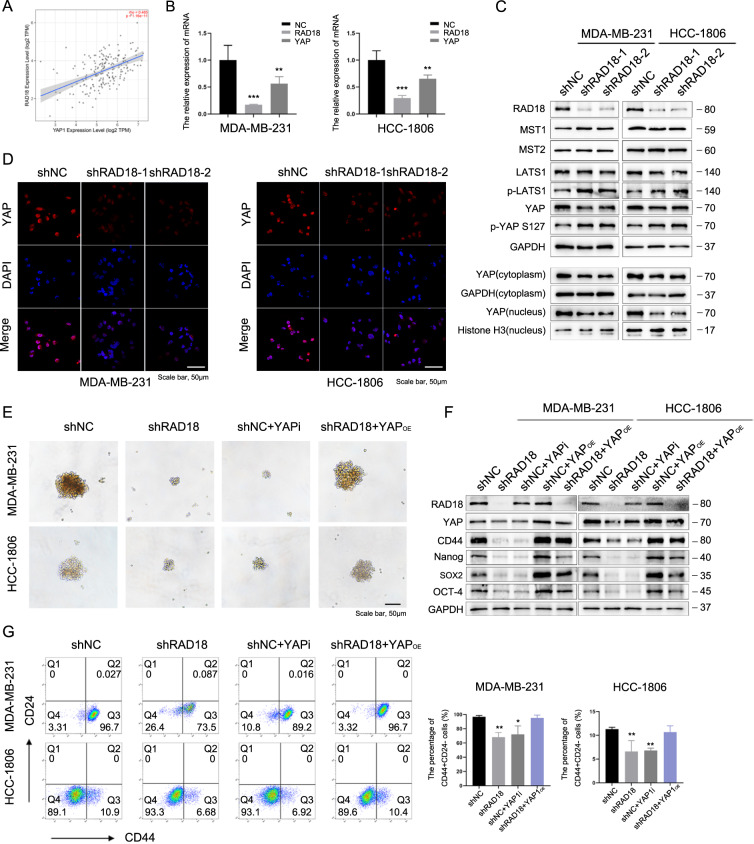
RAD18-induced stemness in TNBC are mediated by Hippo-YAP pathway. RAD18 enhances TNBC cell stemness through the Hippo-YAP pathway. **A** RAD18 was significantly positive correlated with YAP in TNBC according to Timer software (rho = 0.465, *p* = 1.16e-11). **B** Knockdown of RAD18 reduced the mRNA level of YAP through qRT-PCR. **C** Western blot analysis of the expression levels of Hippo-YAP pathway proteins including MST1/2, LATS1, p-LATS1, p-YAP S127, YAP (in total, cytoplasm and nucleus) in shNC and shRAD18 TNBC cell lines. GAPDH levels served as a loading control. **D** Representative fluorescence confocal microscopy images analyzing the YAP (red) expression in shNC and shRAD18 groups as indicated. Nuclei are stained with DAPI (blue). **E**–**G** The tumor sphere forming (**E**), stemness-related proteins levels (**F**), and CD44+/CD24− population percentages (**G**) in the shNC groups treated with verteporfin for 24 h and the shNC/shRAD18 groups treated with YAP overexpression plasmid were performed to determine cell stemness. *p < 0.05, **p < 0.01, ***p < 0.001. All experiments were performed independently at least three times.

To explore whether RAD18 expression affects the stemness of TNBC cells, we measured the percentage of CD44^+^CD24^−^cell population in TNBC cells. The results showed that silencing RAD18 reduced the percentage of the CD44^+^CD24^−^ population from 91.2% to 72.9% in MDA-MB-231 and from 10.1% to 5.05% in HCC1806 cells (*p* < 0.05, Fig. 2F). Moreover, tumor sphere-forming assays were performed to determine the self-renewal properties of CSCs. As shown in Fig. 3G, silencing RAD18 in TNBC cells reduced the number and size of tumor spheres. The influence of RAD18 levels on stemness-associated genes in TNBC was also investigated. The qRT-PCR and WB results proved that silencing RAD18 reduced the expression of stemness-related genes such as CD44, Nanog, SOX2, and OCT4 in TNBC cells (Fig. 2H–I).

Overall, the results demonstrated that RAD18 plays an important role in the maintenance of the stem cell characteristics of TNBC.

### RAD18-induced stemness in TNBC are mediated by Hippo-YAP pathway

The Hippo-YAP pathway is a key regulator of organ size, tissue growth, and stem cell maintenance [20, 21]. YAP signaling has been found to play a fundamental role in favoring the acquisition of CSC features and contributing to the aggressiveness of TNBC [22–24].

The association between the expression of RAD18 and YAP in TNBC was detected using Timer software. RAD18 was significantly positively correlated with YAP (rho = 0.465, *p* < 0.05, Fig. 3A). Knockdown of RAD18 markedly decreased YAP expression in TNBC cells (Fig. 3B). To further uncover the potential molecular mechanism, we analyzed the specific alterations along the Hippo-YAP signaling pathway under the impact of RAD18. As shown in Fig. 3C, the expression of p-LATS1, which can phosphorylate downstream YAP into p-YAP, thus degrading YAP, was inhibited by RAD18.YAP-S127 phosphorylation is a direct readout of p-LATS kinase activity [25]. In compliance with the regulation of p-LATS1 kinase expression, we found that p-YAP-S127 increased in RAD18-silenced cells. Accordingly, the knockdown of RAD18 markedly reduced the level of YAP in the nucleus. IF staining results also verified that the knockdown of RAD18 in TNBC cells significantly reduced YAP expression in comparison to control cells (Fig. 3D).

To further confirm that RAD18-induced Hippo-YAP activation was a potent driving force for TNBC stemness maintenance, the shNC group was treated with a YAP-specific inhibitor verteporfin [26], and tumor sphere formation, stemness-related gene levels, and CD44^+^CD24^−^ population percentage were then detected. The results showed that verteporfin greatly reduced stemness-related protein expression, reduced CSC percentage, and prevented tumor sphere-forming capacity. Consistent with these observations, overexpression of YAP by plasmid in the shRAD18 group significantly enhanced RAD18-inhibited TNBC cell stemness and sphere-forming capacity (Fig. 3E–G).

Overall, these findings confirmed that RAD18 may activate the Hippo-YAP pathway, followed by an increased expression of YAP, especially in the nucleus, thus enhancing tumor stemness and proliferation in TNBC cells.

### RAD18-mediated YAP activation in TNBC cells promotes M2-like TAM polarization

It was recently demonstrated that a high-stemness signature correlates with a poor immunogenic response across 21 solid malignancies, highlighting a potential cross-talk between these two pro-tumorigenic pathways [27]. Based on this association, RAD18 overexpression may not only impact cancer cell stemness, but also extend to play a role in the TME.

**Fig. 4:**
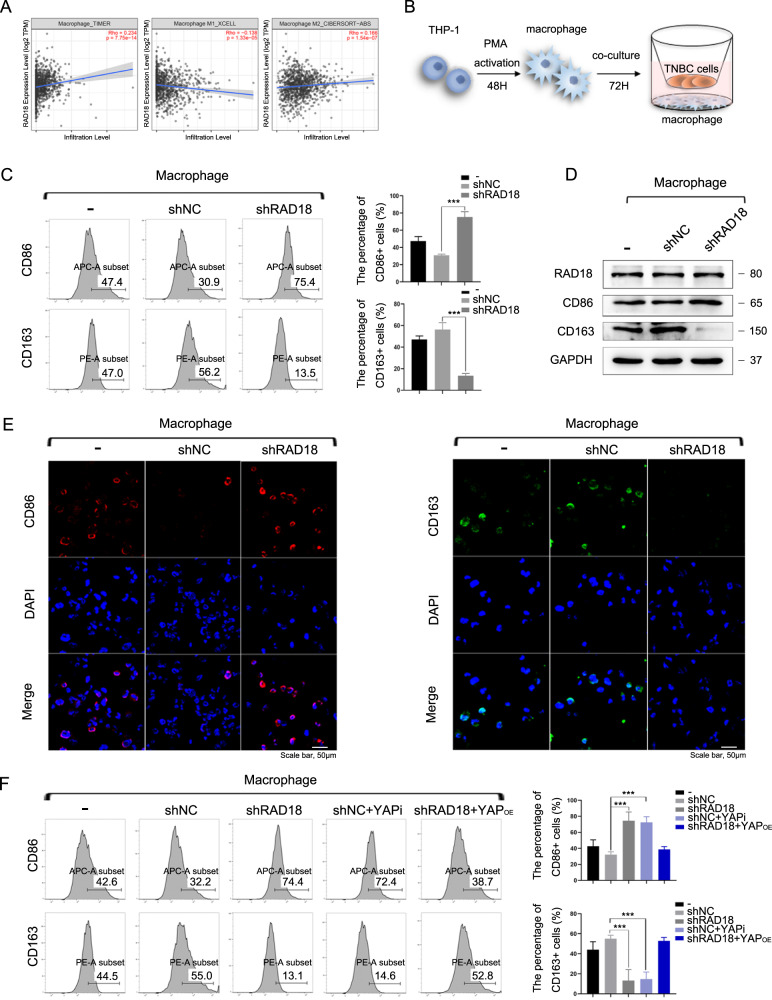
RAD18-driven YAP activation in TNBC cells promotes M2-like TAM polarization. **A** The Timer software showed that high-expressed RAD18 was correlated with high-level macrophage infiltration in BRCA. RAD18 expression level was proportional to M2-like TAM infiltration, whereas inversely proportional to M1-like TAM infiltration. **B** Schematic model of the co-culture system of macrophages and TNBC cells; THP-1 cells were stimulated by PMA for 48 h to form M0 macrophages, then MDA-MB-231 cells stably expressing shNC or shRAD18 were co-cultured with M0 macrophages for 72 h. The M0 alone were used as a negative control. **C** Detection of the expression of CD86 (M1-like macrophage marker) and CD163 (M2-like macrophage marker) in co-cultured macrophages by Flow cytometry analysis. **D** WB detection of protein expression of CD86 and CD163 in macrophages after co-culture. **E** The expressions of CD86 (red) and CD163 (green) in macrophages were detected using immunofluorescence staining (400×). Cell nuclei were stained with DAPI (blue). **F** The expression of CD86 and CD163 in macrophages co-cultured with the shNC TNBC treated with verteporfin or the shRAD18 TNBC treated with YAP^OE^ plasmid were detected by flow cytometry analysis. *p < 0.05, **p < 0.01, ***p < 0.001. All experiments were performed independently at least three times.

Six common cell subsets in TME were screened and are listed in Fig. S1. The association between RAD18 expression and cell infiltration level in BRCA was detected using Timer software. According to the Rh values, high expression of RAD18 was correlated with high-level macrophage infiltration in BRCA (rho = 0.234, *p* < 0.05, Fig. 4A). More specifically, the expression level of RAD18 was proportional to M2-type macrophage infiltration and inversely proportional to M1 type macrophage infiltration (Fig. 4A).

**Fig. 5:**
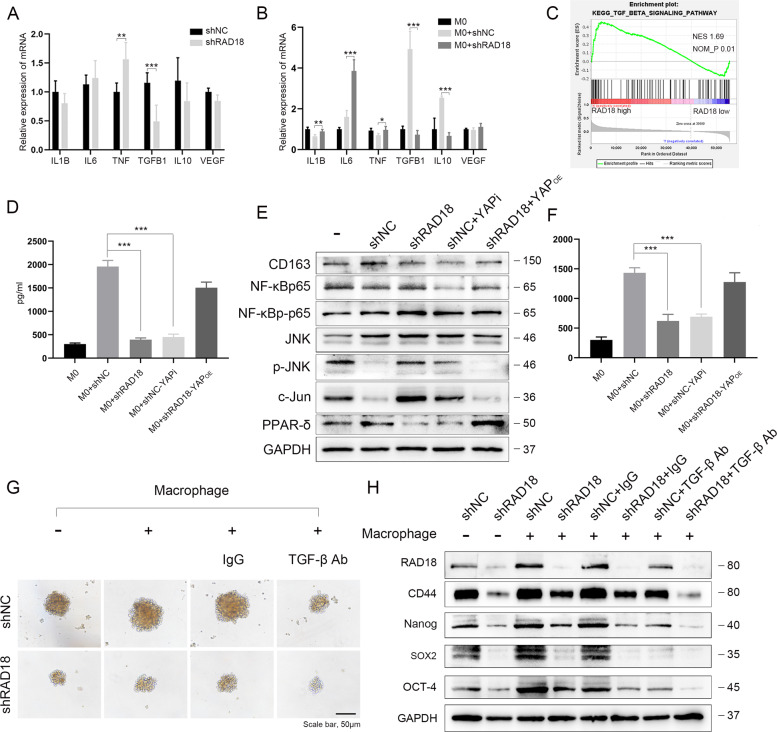
RAD18^High^ TNBC promotes M2-like TAM polarization via TGF-β secretion and JNK pathway inhibition, inducing more TGF-β secreted from M2-like TAM reciprocally upregulates RAD18 to form a positive regulatory loop in the TNBC-TAM interaction. **A**, **B** qRT-PCR was used to detect the expression of cytokines in both TNBC cells and macrophages after co-cultivation. The THP1- derived Mφ co-cultured with shNC and shRAD18 MDA-MB-231 for 3 d. The primary THP-1-derived Mφ (M0) was used as control (**B**). **C** Gene set enrichment analysis plot showing enriched TGF-β signaling pathway in the TCGA cohorts. **D** Detection of TGF-β in co-cultured supernatant by ELISA. **E** WB detection of protein expression levels of CD163, PPAR-δ, NF-κBp65, NF-κBp-p65, JNK, p-JNK, and c-Jun in macrophages. **F** ELISAs were used to determine the TGF-β concentration in supernatant secreted by macrophage alone after co-cultivation. **G**, **H** The tumor sphere formation and protein levels of stemness-related signature genes (CD44, OCT4, SOX2, Nanog) were detected to verify the stemness of shNC/shRAD18 MDA-MB-231 co-cultured with or without macrophages in the absence or presence of a neutralizing antibody specific for TGF-β are presented. *p < 0.05, **p < 0.01, ***p < 0.001. All experiments were performed independently at least three times.

To explore the potential impact of RAD18 in TNBC cells on the modulation of macrophage differentiation, we adopted the human THP-1 monocyte–macrophage differentiation model. A schematic is shown in Fig. 4B. First, THP-1 cells were treated with PMA for 48 h to differentiate into macrophage-like cells displaying an adherent phenotype with extended pseudopods (Fig. S2). THP-1 macrophages were co-cultured with shNC/shRAD18 MDA-MB-231 cells for another 72 h. Flow cytometry assay revealed a relative high expression of CD163 (M2 marker) in the Mφ macrophages co-cultured with shNC MDA-MB-231 (56.2%) cells and a notably low level in the shRAD18 group (13.5%). Meanwhile, the ratio of CD86 + Mφ (M1-like phenotype) increased significantly from 30.9% in the shNC group to 75.4% in the shRAD18 group (Fig. 4C). The results of WB and IF staining also verified that CD86 was upregulated and CD163 was downregulated after co-cultivation with RAD18-inhibition TNBC cell (Fig. 4D, E). Moreover, the shNC+YAPi and shRAD18+YAP^OE^ groups were used to verify whether RAD18-mediated YAP activation might be involved in the M2 polarization process (Fig. 5F).

The results described above indicated that inhibition of the RAD18-YAP axis in TNBC might reduce the M2-like TAM polarization and reprogram Mφ to the M1 phenotype.

### M2 polarization by RAD18-mediated YAP activation might be involved in TGF-β secretion and JNK pathway inhibition

In a previous study [28], 10 TAM polarization factors were screened, as shown in Fig. S3. The relationship between these factors and RAD18 expression in BRCA was detected using the Timer software. TNF, TGF-β1, IL1B, IL10, and VEGFA were highly correlated with RAD18 (rho > 0.1, *p* < 0.05). We then tested the expression levels of these genes in MDA-MB-231 shNC/shRAD18 groups and three groups of macrophages with or without co-cultivation. According to the comparison, only TGF-β showed significant expression changes in both TNBC cells and macrophages. TGF-β was markedly decreased after RAD18 inhibition (Fig. 5A, B). Gene set enrichment analysis (GSEA) also confirmed the high correlation between RAD18 and TGF-β. Furthermore, the high-expression RAD18 group showed TGF-β signaling pathway enrichment (Fig. 5C). In addition, TNF, IL1B, IL6, and IL10 also showed relatively significant expression changes.

TGF-β levels in the culture supernatant were determined using ELISA. TGF-β levels in the shNC group were significantly higher than those in the shRAD18 group (Fig. 5D). The results of WB showed that compared to macrophage-alone, co-cultivation with shNC MDA-MB-231 promoted the expression of PPAR-δ and NF-κBp-p65, and inhibited p-JNK and c-Jun expression in macrophages, inducing M2-type macrophage polarization. In contrast, RAD18 intervention in MDA-MB-231 cells promoted p-JNK and c-Jun expression, accompanied by PPAR-δ inhibition in macrophages, inducing M1 type macrophage polarization. However, RAD18 silencing had no obvious effect on the expression of total NF-κBp65 and JNK (Fig. 5E).

Moreover, the YAP inhibitor verteporfin treated in the shNC group and the YAP overexpressed plasmid transfected in the shRAD18 group were used to verify whether the RAD18-mediated YAP activation might be involved in TGF-β secretion and M2 polarization (Fig. 5D, E).

Our study indicated that RAD18-overexpressed TNBC could promote M2 polarization of TAMs, and that YAP activation might play a key role by increasing TGF-β and PPAR-δ and decreasing the JNK pathway (including c-Jun and p-JNK) in macrophages. RAD18 silencing reversed the transformation of M1 to M2 TAMs Hence, RAD18 regulation may represent a promising strategy for TNBC microenvironment remodeling.

### M2-like TAM feedback regulation of RAD18 expression and stemness of TNBC cells via TGF-β

Paracrine signaling in the TME is not limited to tumor cell-derived mediators, and M2-like TAMs are known to secrete pro-tumor growth factors [29]. Therefore, we detected TGF-β released by macrophages in the co-culture system. As expected, the secretion of TGF-β from macrophages was significantly increased in the M0 + shNC MDA-MB-231 co-culture system when compared with the macrophage alone group. However, its levels were inhibited when M0 was co-cultured with shRAD18 MDA-MB-231 cells (Fig. 5F). The results in the shNC + YAPi and shRAD18 YAP^OE^ groups verified that RAD18-mediated YAP activation in TNBC patients affected TGF-β secretion from M2-like TAMs (Fig. 5F).

TGF-β has been reported to be closely related to tumor stemness. For example, TGF-β activates noncanonical SHH signaling by increasing the expression of hedgehog transcription factor GLI2 in CSCs, thus leading to increased stemness by reducing apoptosis and enhancing chemoresistance in CSCs [30]. TGF-β may also maintain the quiescent state of CSCs by phosphorylating SMAD2 and SMAD3 in squamous cell carcinoma. Genetic or pharmacological blocking of TGF-β secretin increases the sensitivity of CSCs to therapeutic agents [31]. To evaluate whether the increased TGF-β in the co-culture system is responsible for promoting the stemness of TNBC cells, MDA-MB-231 cells in the co-culture system were collected. As shown in Fig. 5G, H, using non-cocultivated MDA-MB-231 cells as references, the tumor sphere formation, RAD18 levels, and stemness-related protein levels in the shNC co-cultivation group increased. Moreover, the shRAD18 co-cultivation group, similar to MDA-MB-231 cells alone group, had lower RAD18 levels and stemness than the shNC co-cultivation group. TGF-β in the co-culture system was then blocked using anti-TGF-β-neutralizing antibodies (5 μg/ml) or control IgG antibodies (5 μg/ml) for 48 h. The TGF-β-neutralizing antibody significantly attenuated the expression levels of RAD18 and stemness markers, as well as tumor sphere formation in MDA-MB-231 cells in the co-cultivation system (Fig. 5G).

Therefore, polarized M2-like TAMs in the tumor microenvironment may function as a feedback regulator enhancing RAD18 expression and TNBC stemness via TGF-β.

### In nude mice xenografts, RAD18 promoted tumor progress and M2 polarization

**Fig. 6:**
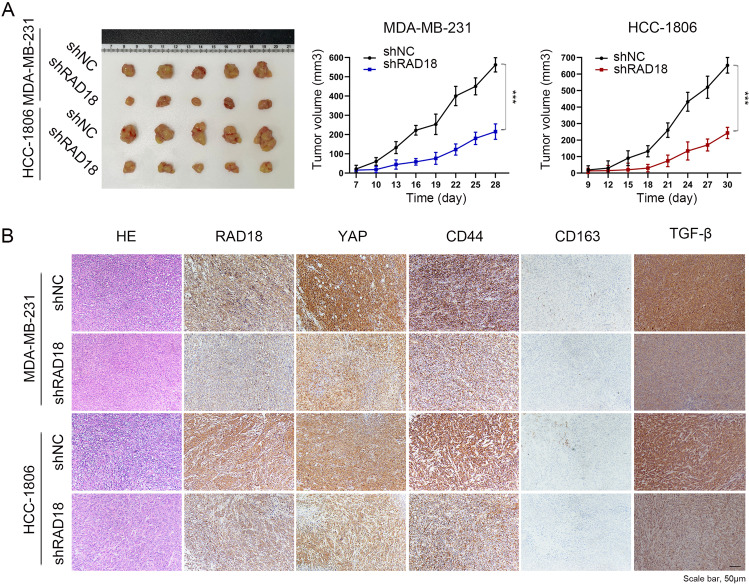
In nude mice xenografts, RAD18 promoted tumor progress and M2 polarization. **A** After 4 weeks, the tumors of nude mice were removed and evaluated by HE staining. Images of dissected xenografted tumors implanted subcutaneously with TNBC cells (shNC/shRAD18 MDA-MB-231 and shNC/shRAD18 HCC-1806) were showed. Tumor growth curves determined by caliper measurements shown as mean (mm^3^) ± SD. *n* = 5/group. **B** H&E and IHC staining of RAD18, YAP, CD44, CD163, and TGFβ in nude mice xenografts tumor samples.

For the purpose of examining the effect of high RAD18 expression on the tumor-promoting effects of TNBC cells in vivo, shNC/shRAD18 TNBC cells were subcutaneously injected into nude mice. Approximately four weeks later, the mice were sacrificed. Notably, the tumor growth in the shNC tumors was rapid and presented with large tumor volumes compared to shRAD18 tumors, which were ~3–4 times the shRAD18 tumor size (*p* < 0.05, Fig. 6A). Hematoxylin and eosin (HE) staining was performed on the tumor tissues. In the shNC group, the morphology of the nucleus was megakaryocytes, binuclear, or polynuclear. A brownish-black nucleus indicates positive Ki67 expression.

**Fig. 7:**
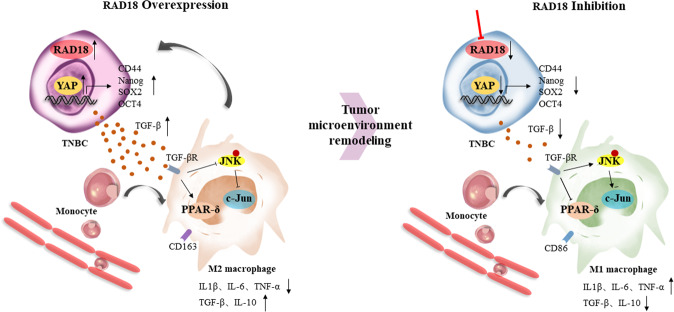
The schematic diagram of the underlying mechanisms described in our study. A positive feedback loop: RAD18-YAP-TGF-β between triple-negative breast cancer and macrophages regulates cancer stemness and progression.

To verify the regulation of RAD18 on the Hippo-YAP pathway in vivo, immunohistochemistry was used to determine the activity of the YAP signaling pathway. A concomitant decrease in YAP and CD44 expression with RAD18 inhibition was observed (Fig. 6B), confirming the close correlation between RAD18-induced Hippo-YAP pathway activation and tumor stemness maintenance. In addition, tumors in the shNC group showed a notable increase in CD163 and TGF-β expression, which was significantly inhibited in the shRAD18 group (Fig. 6B). This means that the proportion of M2 in the shRAD18 group was significantly lower than that in the shNC group. The schematic diagram of the underlying mechanisms described in our study could be seen in Fig. 7.

Overall, these results suggest that RAD18 plays a key role in enhancing the proliferation potential of TNBC cells in vivo by activating the Hippo-YAP pathway, which also mediates the polarization of M2 TAMs and secretion of TGF-β.

## Discussion

RAD18 was first observed in 2002 to bind conjugated ubiquitin to eukaryotic PCNA in response to DNA-damaging agents that cause replication-blocking lesions, thus activating both translesion DNA synthesis (TLS) and template switching [5, 32, 33]. In the ensuing years, extensive efforts were made to uncover the complexity of RAD18. It has also been implicated that RAD18 relays the DNA damage signal to orchestrate DSB repair by homologous recombination through direct interaction with RAD51C. It plays a fundamental role in coordinating DNA damage checkpoint responses during DNA repair [4]. DNA damage response (DDR) and DNA repair are vital for preserving genomic integrity during a normal cell cycle and after genotoxic stress [34]. However, the role of RAD18 in tumorigenesis and progression remains unclear.

In this study, we first discovered that the expression of RAD18 was significantly higher in TNBC than in any other subtype of breast cancer, and the increased RAD18 expression is associated with higher levels of T stage and poor prognosis. Based on these observations, we found a vital role of RAD18 is promoting TNBC cell proliferation and maintenance of stemness. This was consistent with a previous study showing that DNA repair–cancer stem cell crosstalk promotes tumor progression. For example, overexpression of the redox effector factor (Ref-1), an enzyme activated in the base excision repair (BER) pathway, decreases ROS levels in cancer cells, resulting in enhanced cancer cell stemness [35]. Oct4, a stemness-associated transcription factor encoded by the POU5F1 gene, regulates the CSC phenotype and radioresistance by contributing to the HR-mediated DNA repair response [36]. Therefore, specific inhibition of DNA repair pathways could improve tumor response and clinical outcome in cancer patients by interfering with the signal transduction in CSCs.

Yes-associated protein (YAP), a downstream effector of the Hippo pathway, plays a crucial role in regulating cell proliferation, tumorigenesis, stem cell renewal [37, 38]. YAP has been shown to promote breast cancer stemness by upregulating CSC-associated genes and IL-6 through serum response factors [39]. In this study, we found that RAD18 expression positively correlated with that of YAP in vivo and that silencing of RAD18 inhibited the expression of YAP in TNBC cells. Overexpression of YAP by plasmid reversed the low stemness in the shRAD18 group, whereas silencing YAP using verteporfin eliminated the RAD18-induced stemness of TNBC. Collectively, these results provide a potential mechanism underlying RAD18-induced tumor stemness maintenance, which could be driven by enhanced levels of YAP.

Besides its crucial functions, numerous studies have shown that YAP participates in cancer immune regulation. It not only affects immune cell recruitment, but also induces cancer immune evasion [40, 41]. Macrophages are the most abundant cells in the TME and play a crucial role in tumor progression by transforming the M1-like antitumor phenotype to the M2-like pro-tumor phenotype [29, 42]. The M2-like phenotype promotes angiogenesis, migration, and invasion, and is also related to poor prognosis in breast cancer patients [43]. Guo et al. found that YAP activation enhanced M2-like macrophage recruitment during cancer progression [44]. Moreover, it was recently demonstrated that a high-stemness signature is associated with a poor immunogenic response among multiple solid malignancies [27]. Thus, we hypothesized that RAD18-induced YAP activation in TNBC regulates macrophage polarization, thereby inducing the M2-like phenotype and promoting tumor progression. Our results showed a significant increase in M2-like TAM polarization when M0 cells were co-cultured with shNC cells. As expected, the proportion of M2-like TAMs was markedly inhibited when RAD18 was knocked down. RAD18 silencing promotes macrophage transformation from a pro-tumor to an antitumor phenotype. Furthermore, the M2 polarization factors were investigated. TGF-β is a cytokine that promotes the differentiation of non-activated macrophages into M2 TAMs [45–47]. The expression of TGF-β in the co-culture supernatants was related to RAD18. Consistent with previous results, interference of RAD18 resulted in a decrease in TGF-β levels. Moreover, c-Jun and PPAR-δ are activators of the interconversion of M1 and M2 macrophages. Previous studies have shown that the activation of NF-κB and JNK pathways could increase c-Jun levels, thus promoting M1-like phenotype polarization. Conversely, inhibition of the JNK pathway proceeds to M2-like phenotype polarization [48, 49]. PPAR-δ is activated in DDR and acts as an immunosuppressive agent by inhibiting the production of pro-inflammatory factors [50]. In this study, co-cultivation with shNC TNBC cells induced the conversion of macrophages to M2-like phenotype TAMs and was mainly mediated by promoting PPAR-δ and NF-κBp-p65 levels and decreasing c-Jun and p-JNK levels in macrophages. However, the interference of RAD18 inhibited PPAR-δ and promoted c-Jun and p-JNK expression, suggesting RAD18-induced M2-macrophages polarization mainly through inhibition of the JNK pathway, rather than the NF-κB pathway.

The role of M2-like TAMs in promoting tumor growth has been widely investigated [29, 51]. M2-like TAMs release a panel of pro-tumor cytokines, including TGF-β, to promote tumor progression [52]. In this study, we found that the level of TGF-β was significantly increased in the M0 cell medium after coculturing with shNC MDA-MB-231 cells, which could be functionally related to the high level of RAD18 in TNBC cells. The results of our study suggest the positive feedback loop between RAD18 and TAMs plays a vital role in cancer progression, and that it operates through a mutual action between DDR and TAMs. Consistent with previous studies, DDR can lead to the production of cancer-related inflammation, which induces TME-secreting factors such as TNF, IL-1, CCL2, and CXCL8; and activates transcription factors of NF-Κb, Stat-3, thus promoting tumor proliferation [53]. TME can also regulate the DDR pathway. For example, TGF-β is the most common cytokine secreted in the TME, which controls the DDR pathway by regulating ATM-CHK2 [54].

In summary, this study suggests that a novel vicious cycle exists between RAD18, stemness maintenance and M2-like TAM polarization. Here, we demonstrate that high RAD18 expression not only facilitates a highly stem-cell phenotype, which in its turn facilitates the proliferation of TNBC, but also that its cytokine byproduct, TGF-β, promotes M2-like TAM polarization and feedback activation of RAD18 in tumor cells to enhance tumor stemness. The RAD18-YAP-TGF-β loop is essential for cancer cells to maintain or promote their CSC phenotype and could be a potential therapeutic target for TNBC.

## Supplementary information

Supplementary legends

Figure S1

Figure S2

Figure S3

Figure S4

Figure S5

Figure S6

Figure S7

Figure S8

Figure S9

Supplementary Figure 1

Supplementary Figure 2

Supplementary Figure 3

Supplementary Fugure 4

Supplementary Figure 5

Original Data File

Original Data File

Original Data File

## Data Availability

All data generated or analyzed during this study are included in this article and its Supplementary Information files.
