# Supplementary material for: A positive feedback loop: RAD18-YAP-TGF-β between triple-negative breast cancer and macrophages regulates cancer stemness and progression
Source: Cell Death Discov. 2022 Apr 12;8:196. doi: 10.1038/s41420-022-00968-9 (PMC9005530; doi:10.1038/s41420-022-00968-9)
Supplement: Supplementary file 1 — Supplementary legends [file 41420_2022_968_MOESM1_ESM.docx]

**Fig.S1.** The association between RAD18 expression and six common cell subsets infiltration level in BRCA TME was detected by Timer software.

**Fig.S2.** The treatment of PMA to THP-1 cells for 48 h results in differentiated Mφ macrophage-like cells displaying an adherent phenotype with extended pseudopods.

**Fig.S3.** The correlation between ten TAMs polarization factors and RAD18 expression in BRCA was detected by Timer software.

**Fig.S4.** The effect of RAD18 shRNA on cell cycle distributions in TNBC cells.

**Fig.S5.** The role of RAD18 in several CSC-associated pathways (NOTCH, Wnt/β-catenin, Hedgehog and Hippo/YAP pathway).

**Fig.S6.** The mRNA and protein expression of YAP target genes (CYR61 and CTGF) in genetic and pharmacological perturbations of YAP.

**Fig.S7.** Exogenous overexpression of RAD18 in the RAD18 knockdown cells can rescue the reduction in tumor-sphere formation.

**Fig.S8.** The viability of macrophages in the co-culture system was not affected by RAD18 knockdown in the TNBC cells.

**Fig.S9.** The densitometric analysis of all the WB results in our study.
