# Supplementary figures and images for: A positive feedback loop: RAD18-YAP-TGF-β between triple-negative breast cancer and macrophages regulates cancer stemness and progression
Source: Cell Death Discov. 2022 Apr 12;8:196. doi: 10.1038/s41420-022-00968-9 (PMC9005530; doi:10.1038/s41420-022-00968-9)

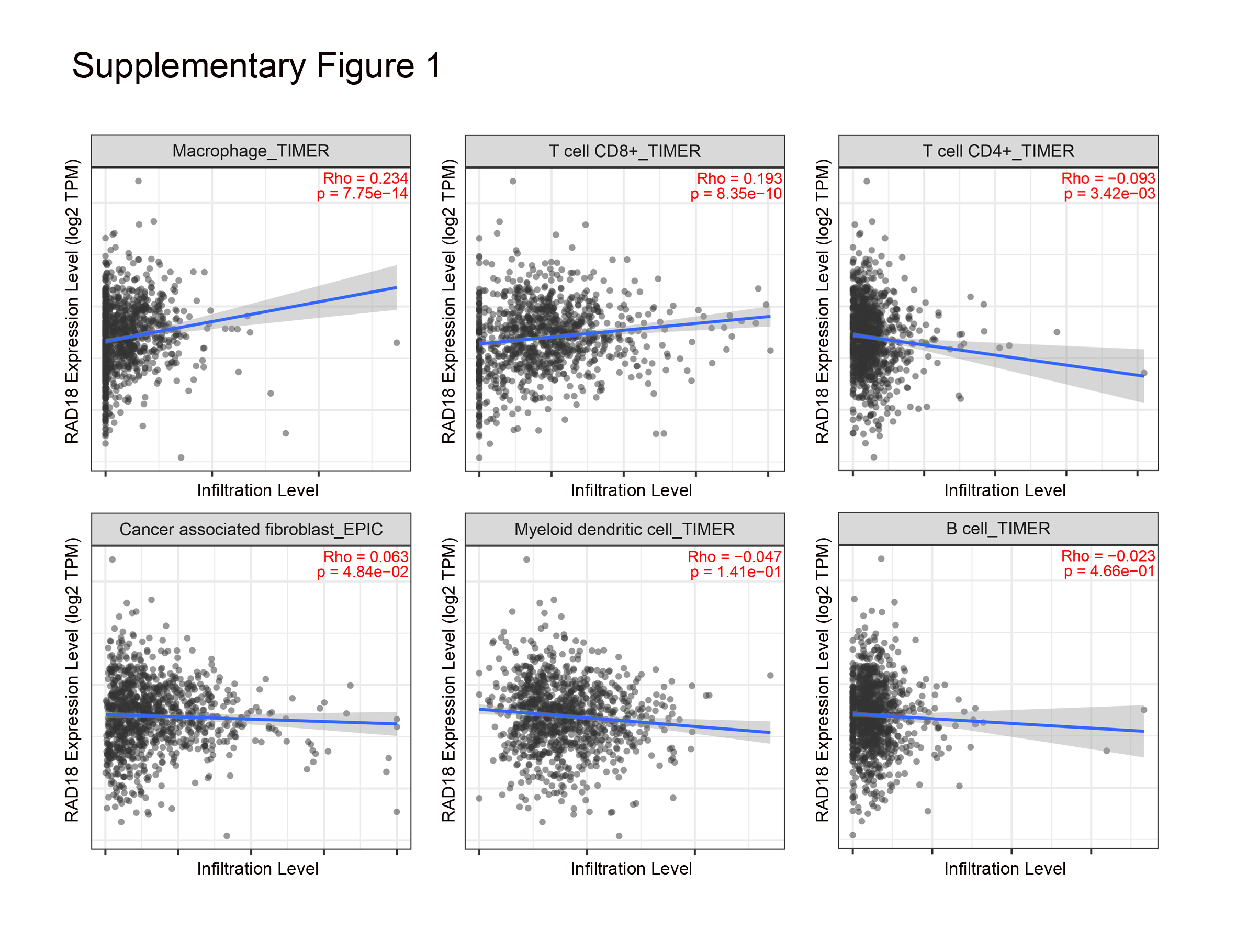

Supplement: Supplementary file 2 — Figure S1 [file 41420_2022_968_MOESM2_ESM.tif]

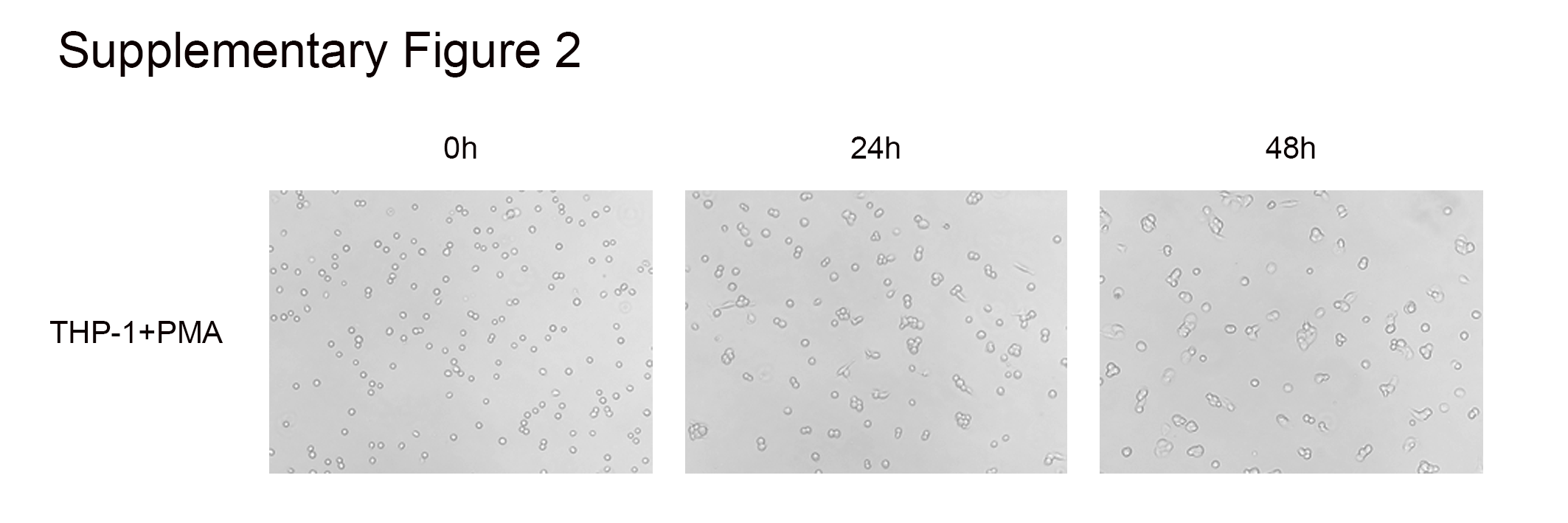

Supplement: Supplementary file 3 — Figure S2 [file 41420_2022_968_MOESM3_ESM.tif]

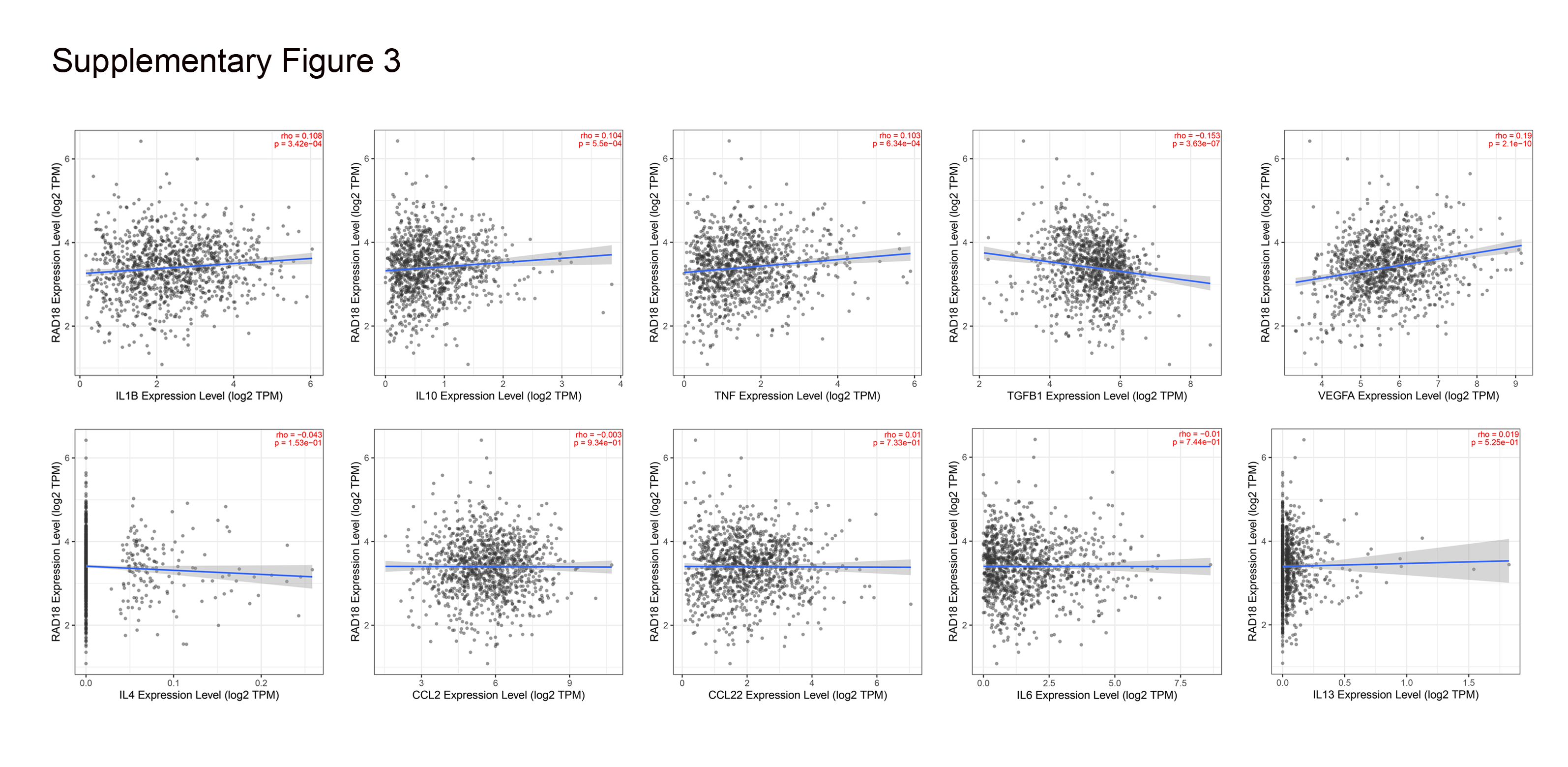

Supplement: Supplementary file 4 — Figure S3 [file 41420_2022_968_MOESM4_ESM.tif]

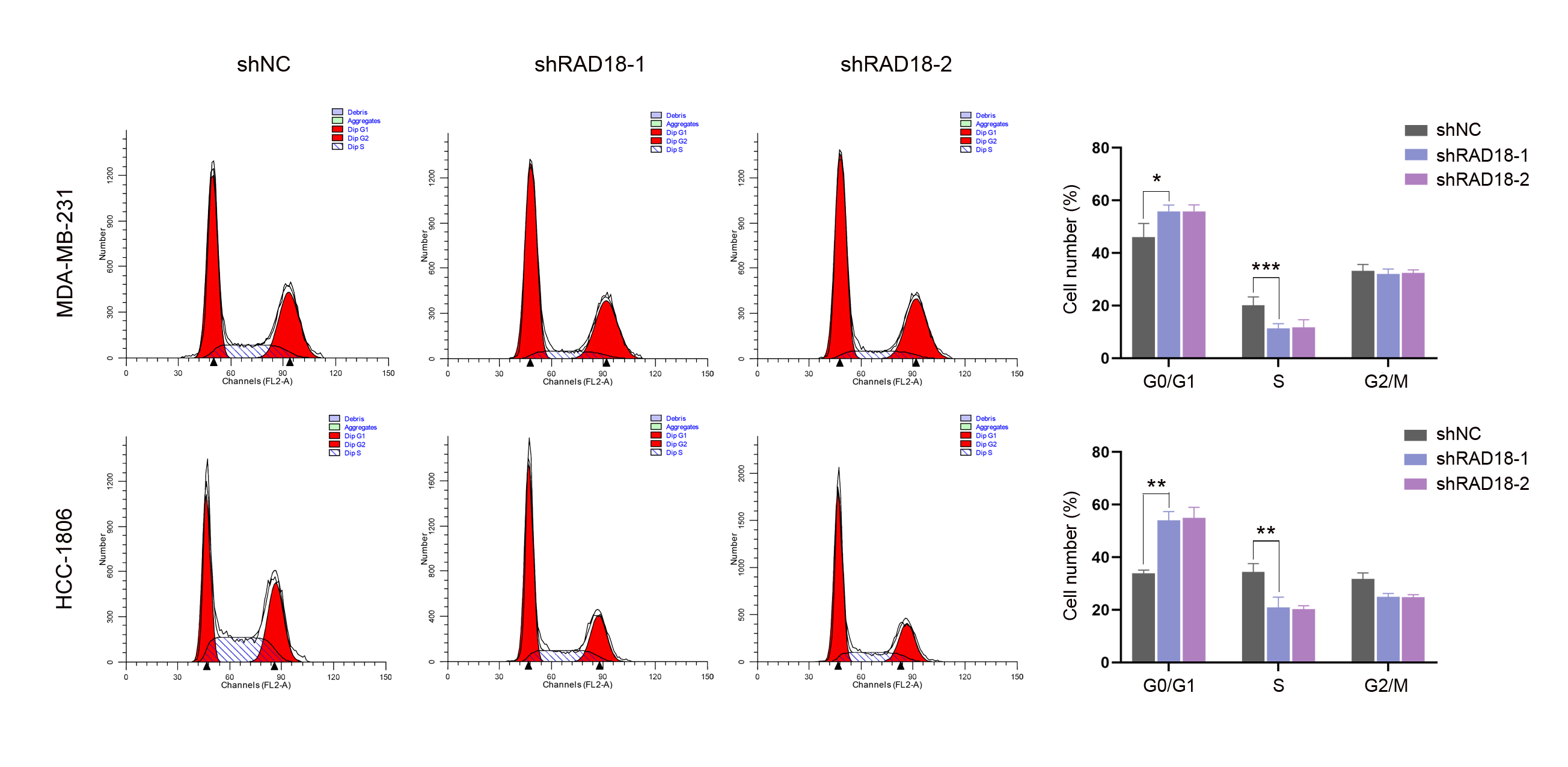

Supplement: Supplementary file 5 — Figure S4 [file 41420_2022_968_MOESM5_ESM.tif]

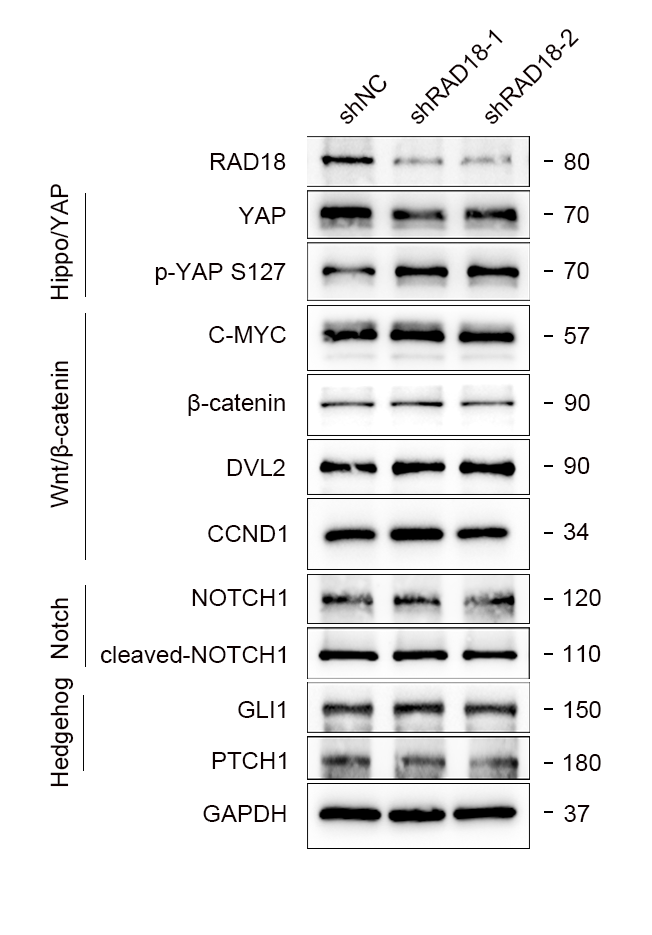

Supplement: Supplementary file 6 — Figure S5 [file 41420_2022_968_MOESM6_ESM.tif]

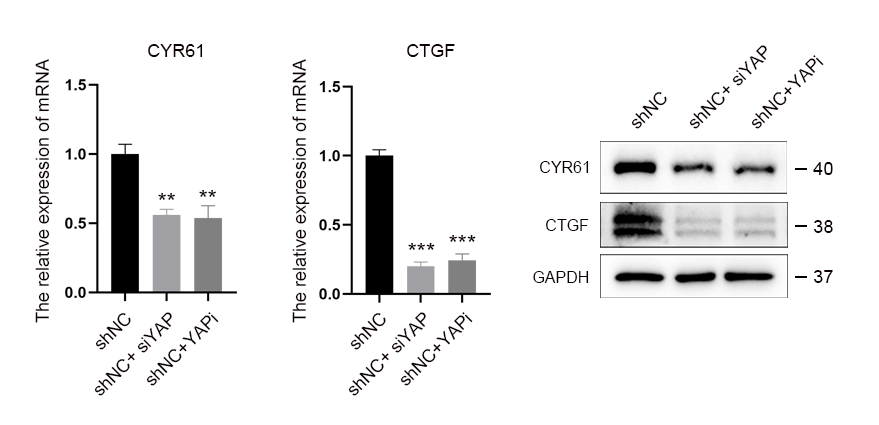

Supplement: Supplementary file 7 — Figure S6 [file 41420_2022_968_MOESM7_ESM.tif]

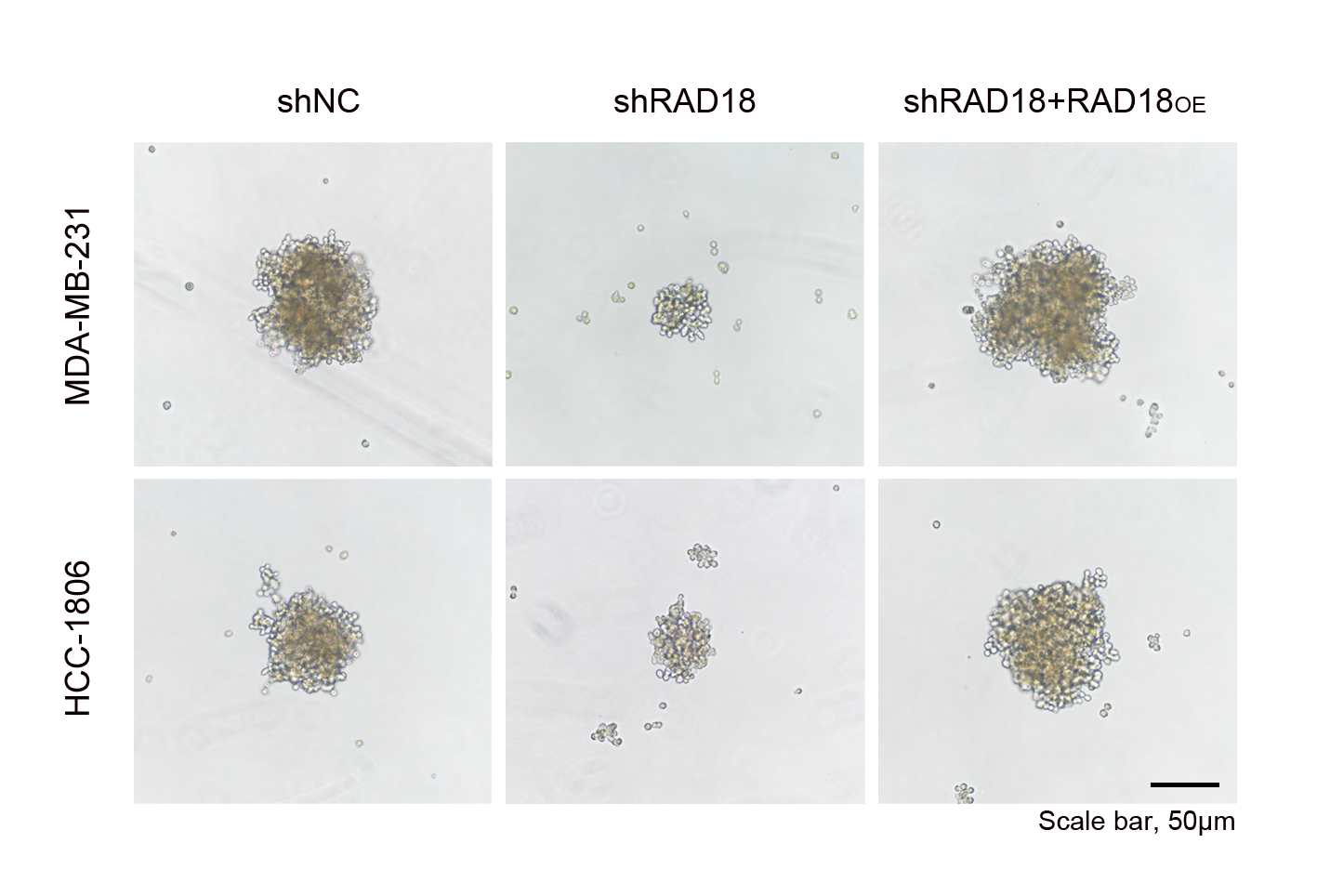

Supplement: Supplementary file 8 — Figure S7 [file 41420_2022_968_MOESM8_ESM.tif]

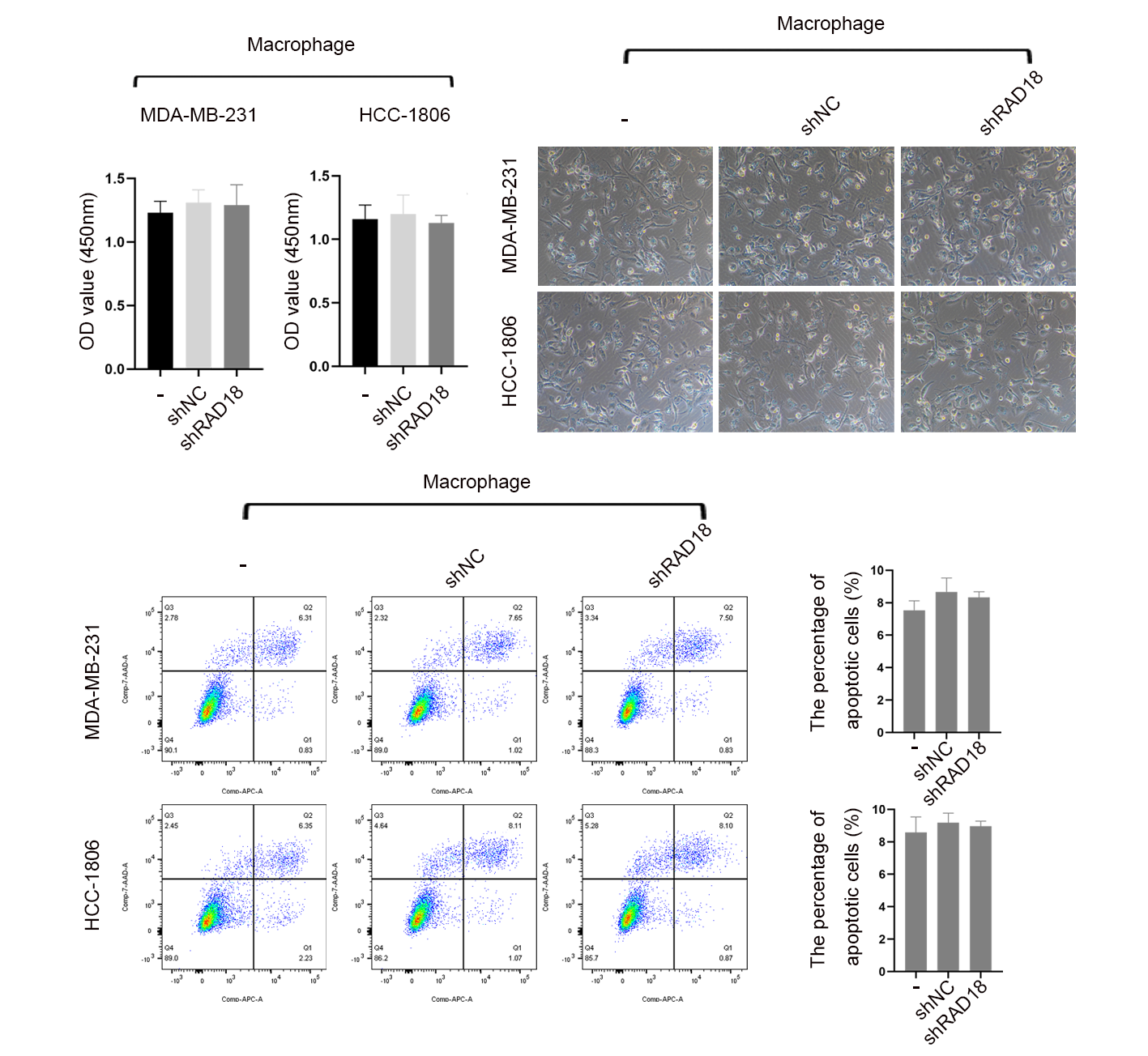

Supplement: Supplementary file 9 — Figure S8 [file 41420_2022_968_MOESM9_ESM.tif]

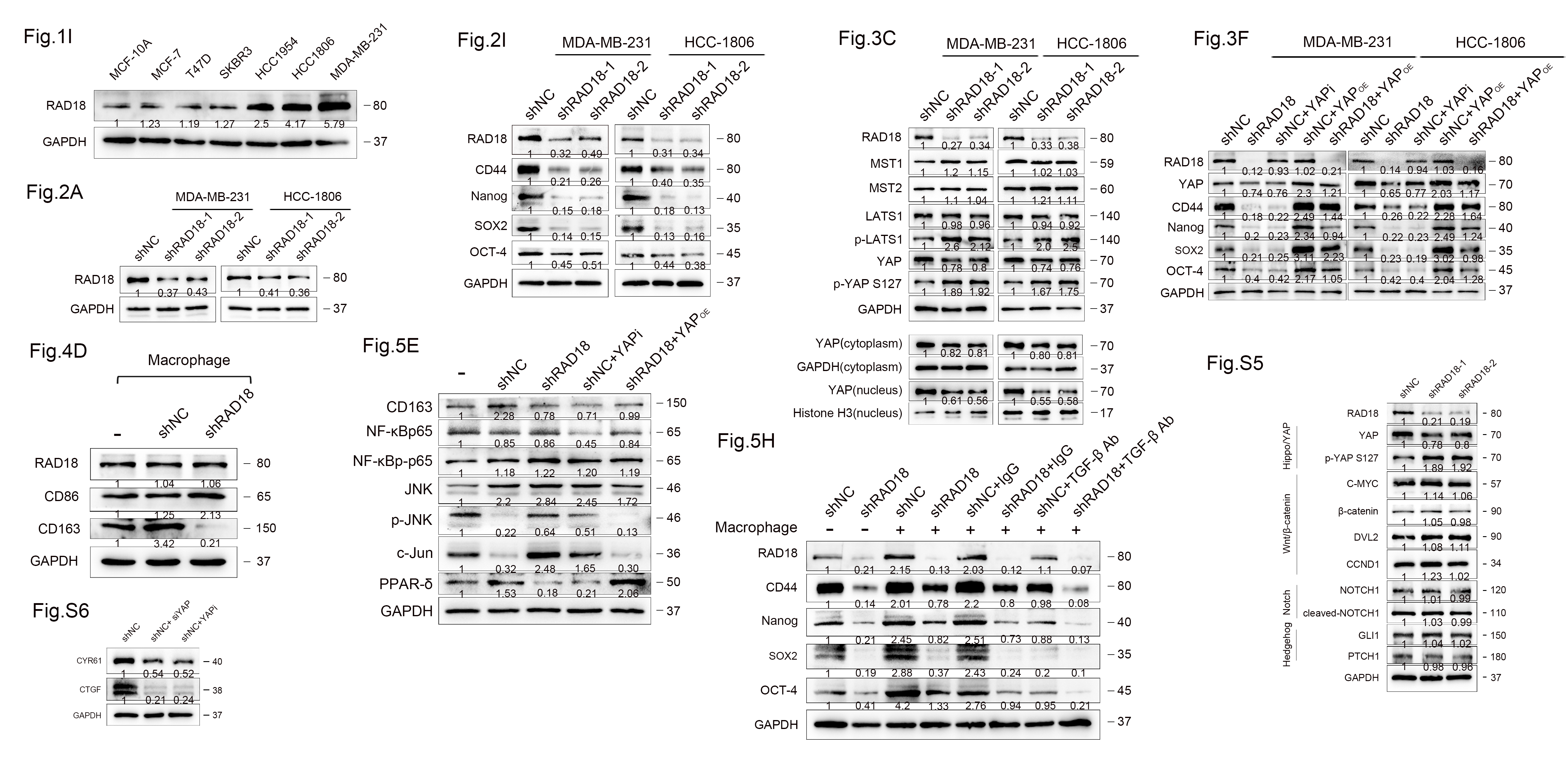

Supplement: Supplementary file 10 — Figure S9 [file 41420_2022_968_MOESM10_ESM.tif]

Figure. 1I-GAPDH

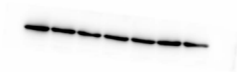

Figure. 1I-RAD18

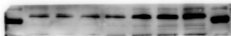

Supplement: Supplementary file 11 — Supplementary Figure 1 [file 41420_2022_968_MOESM11_ESM.pdf]

Figure.4D-CD86

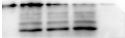

Figure.4D-CD163

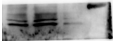

Figure.4D-GAPDH

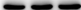

Figure.4D-RAD18

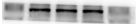

Supplement: Supplementary file 14 — Supplementary Fugure 4 [file 41420_2022_968_MOESM14_ESM.pdf]

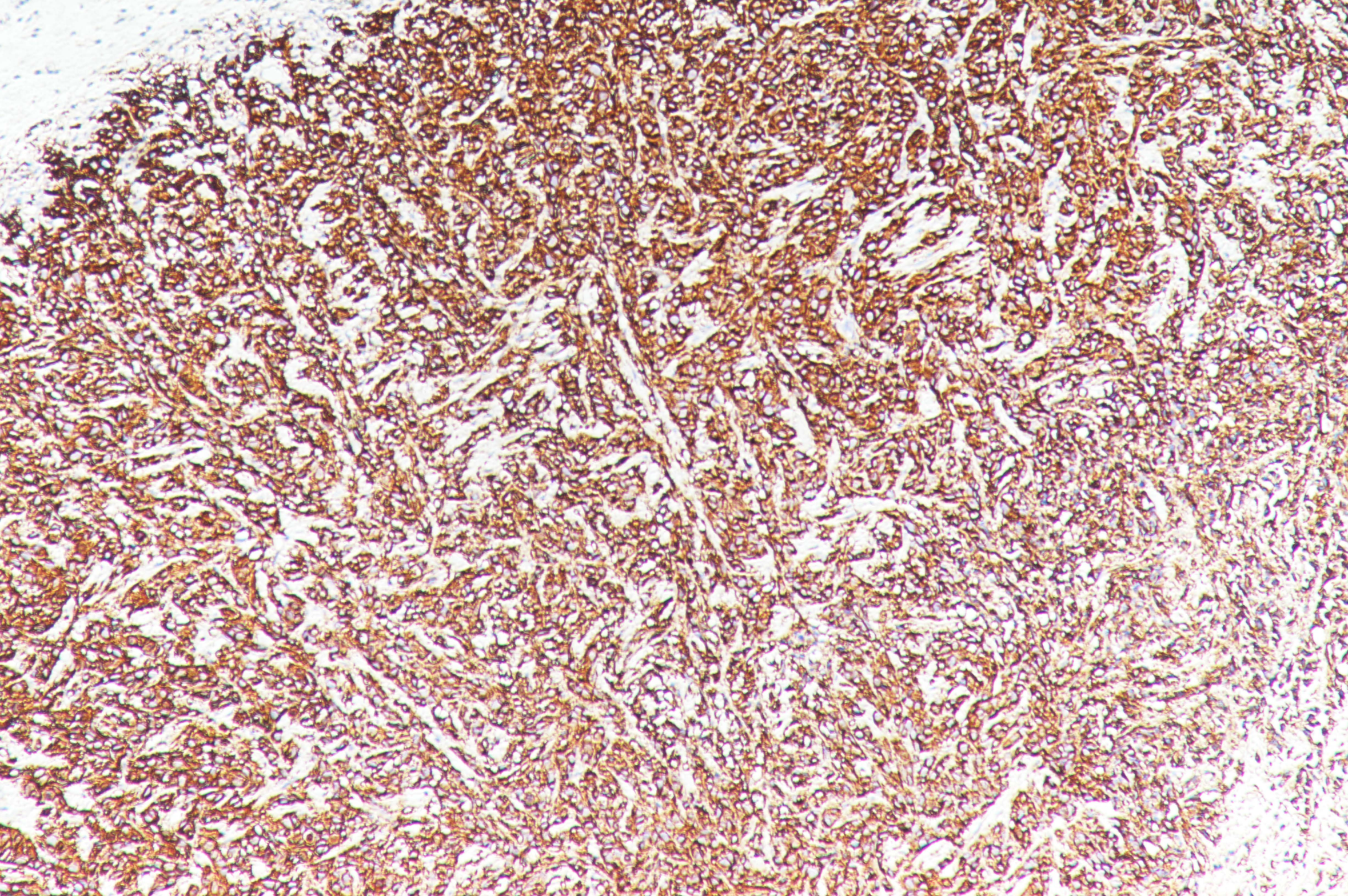

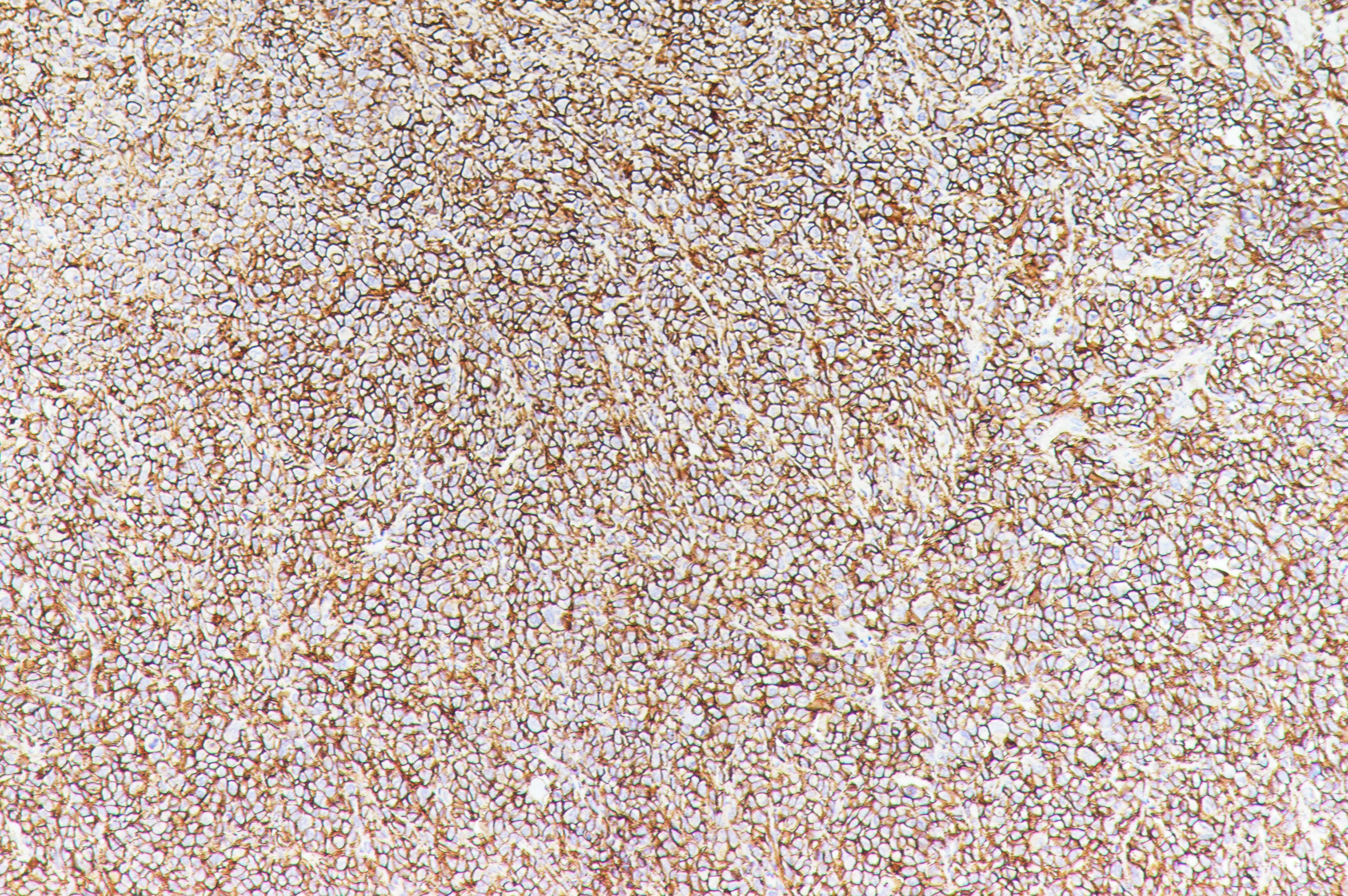

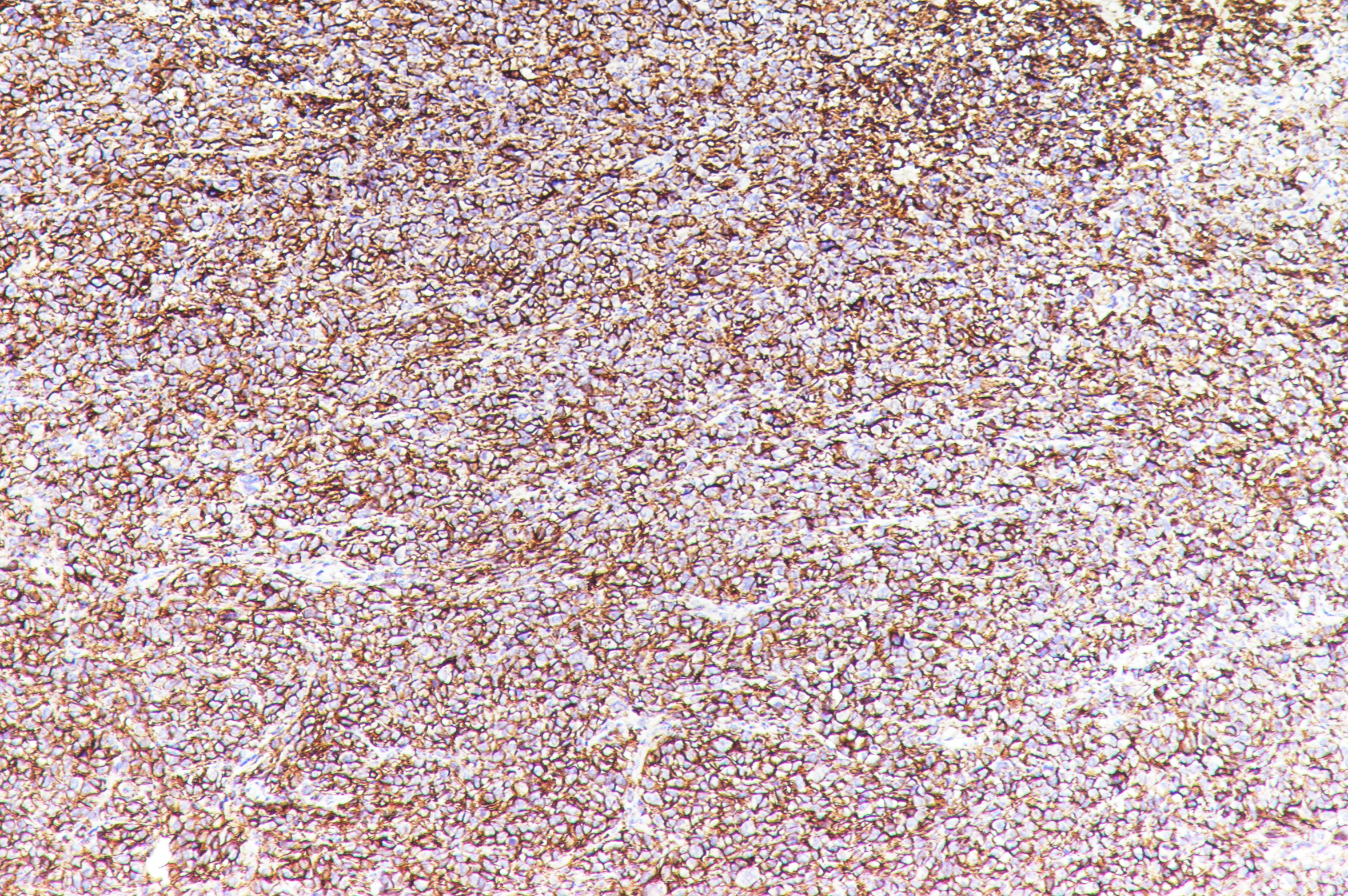

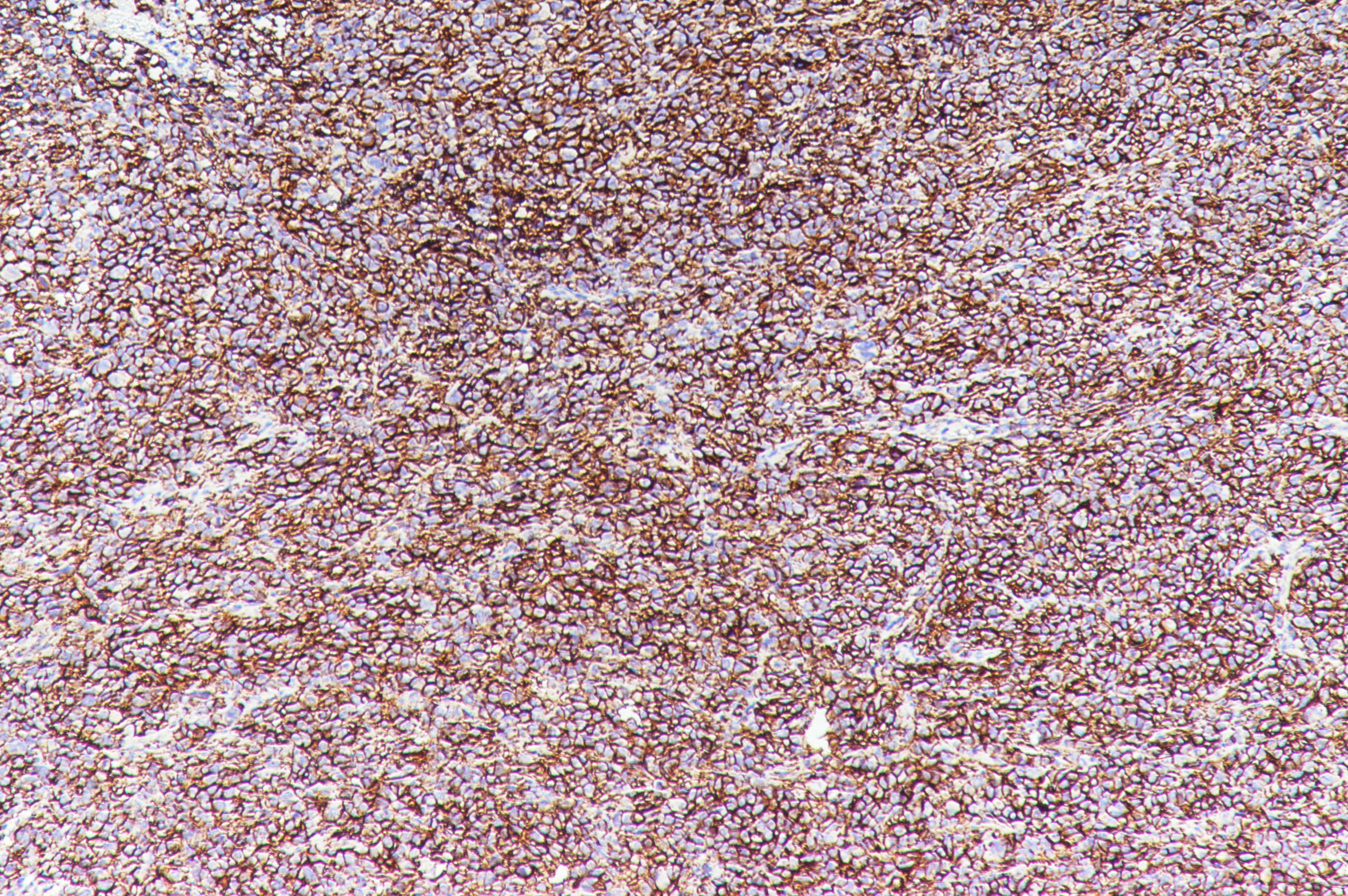









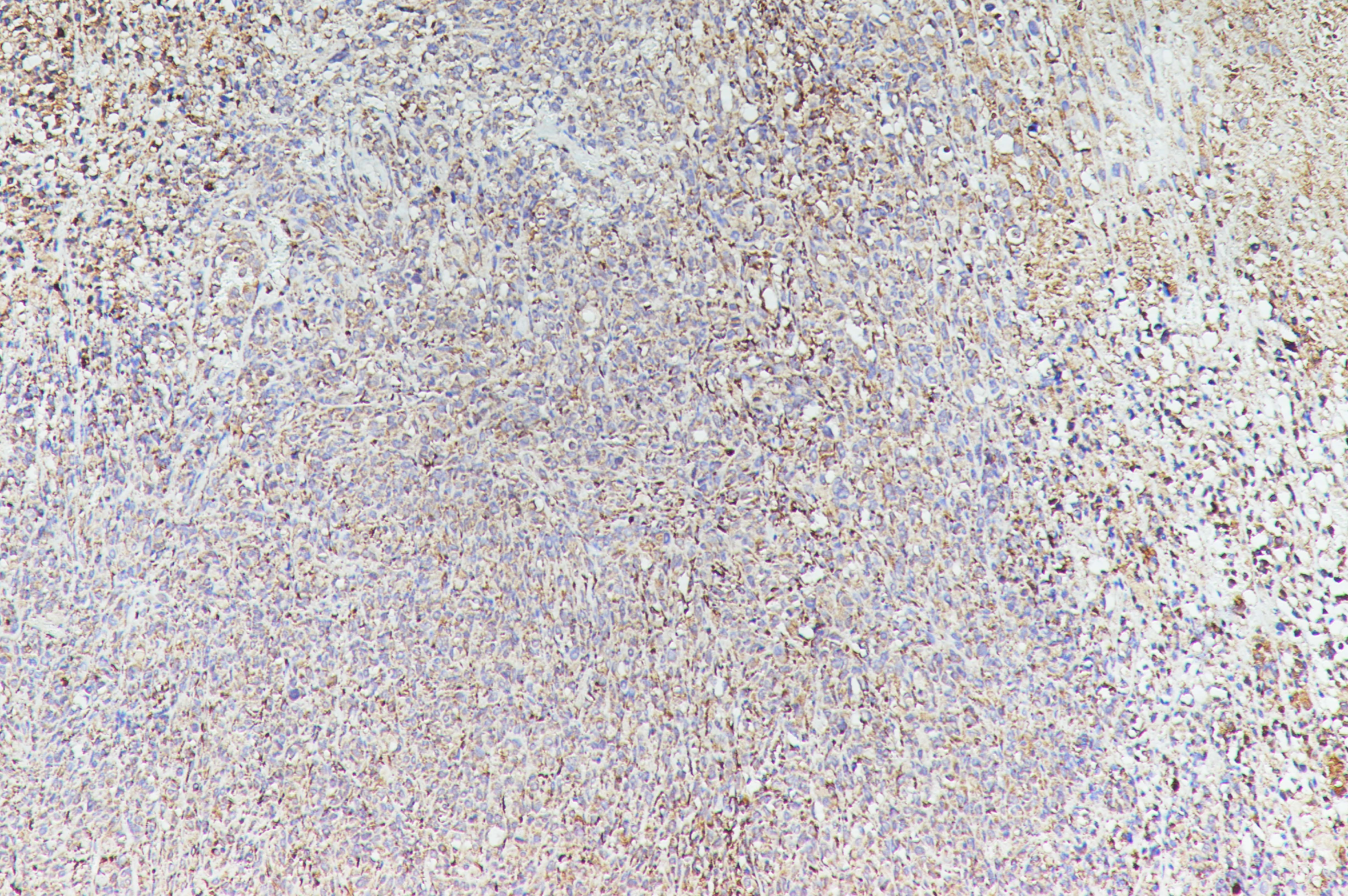

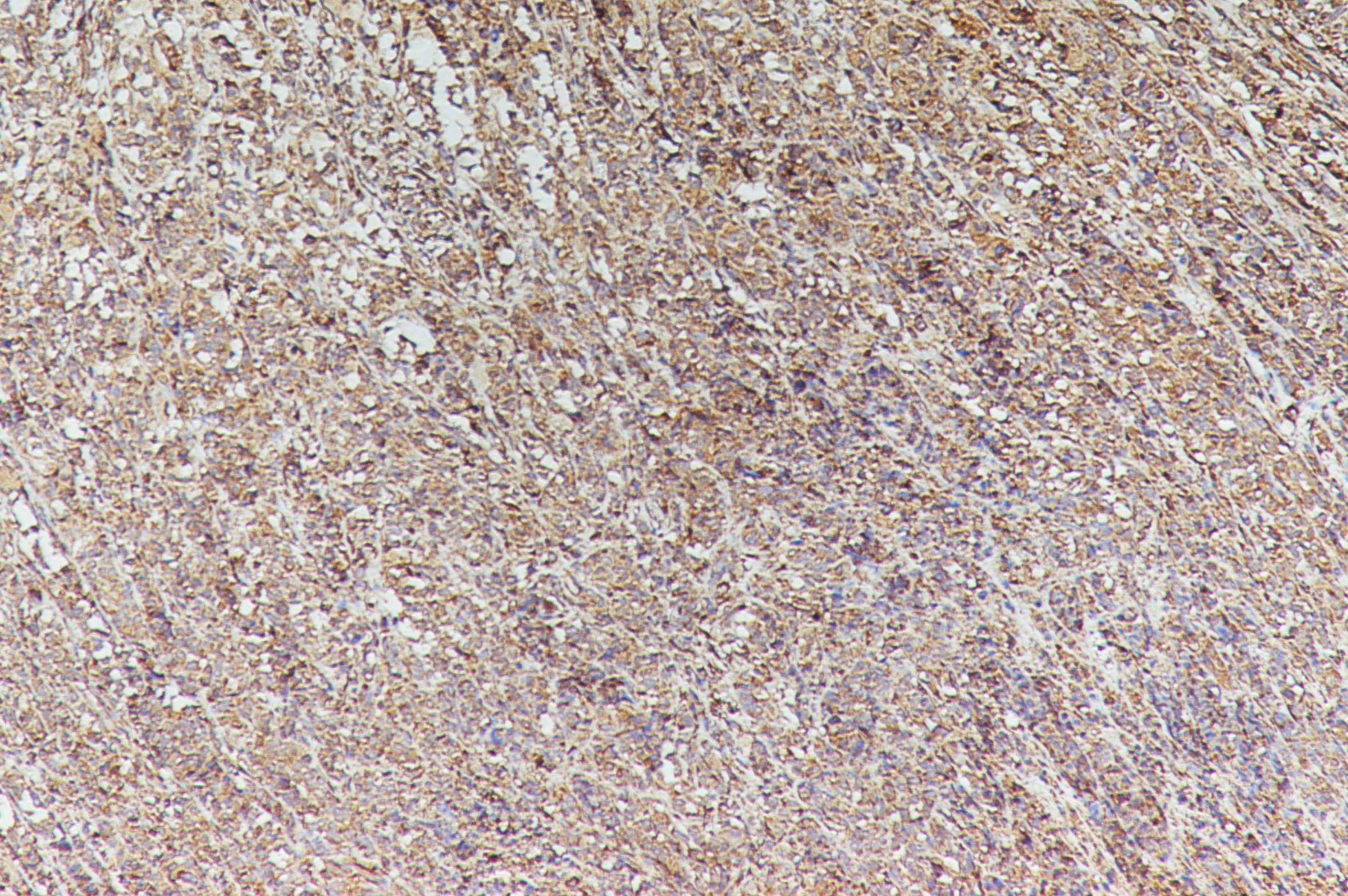

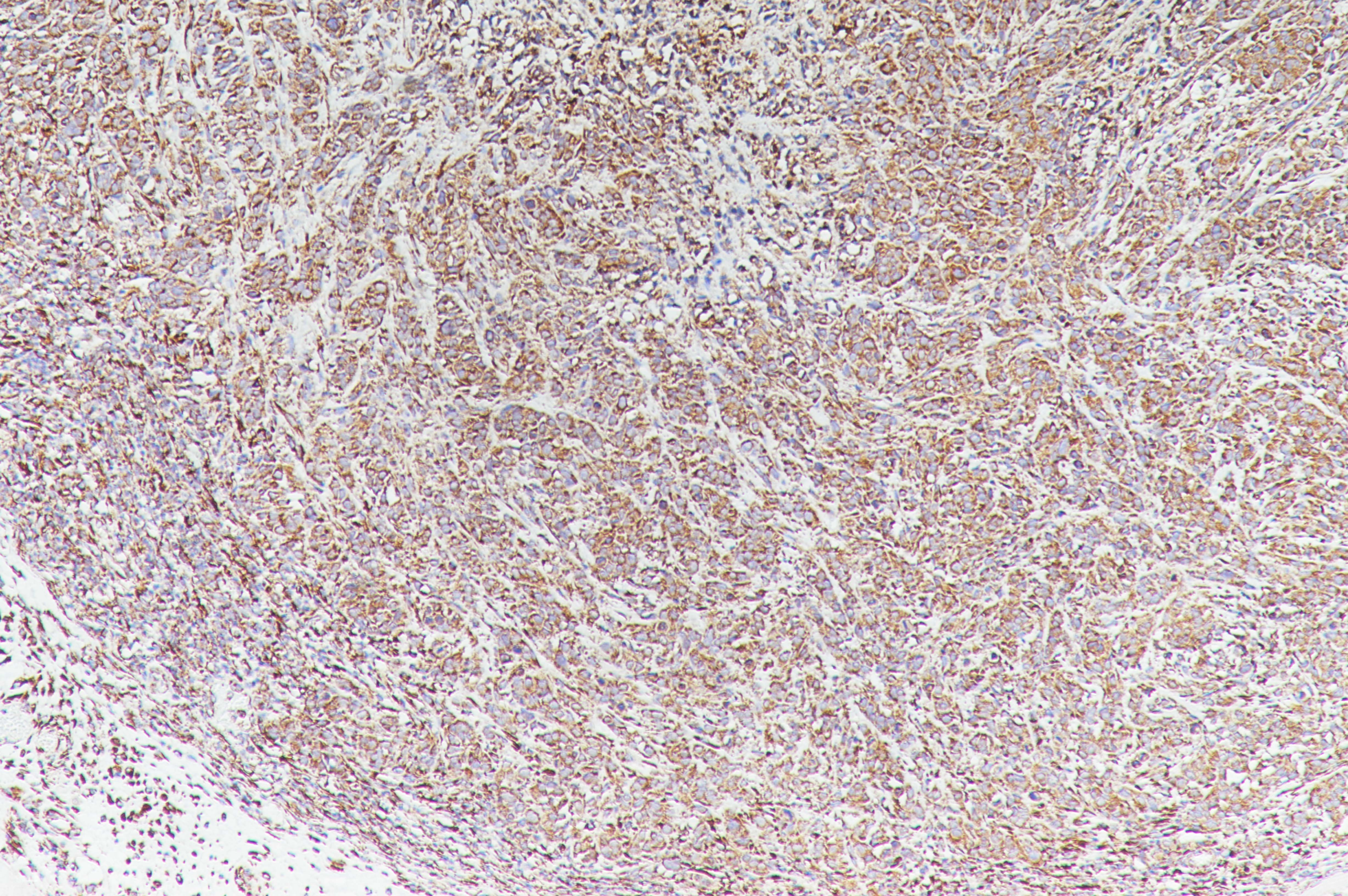

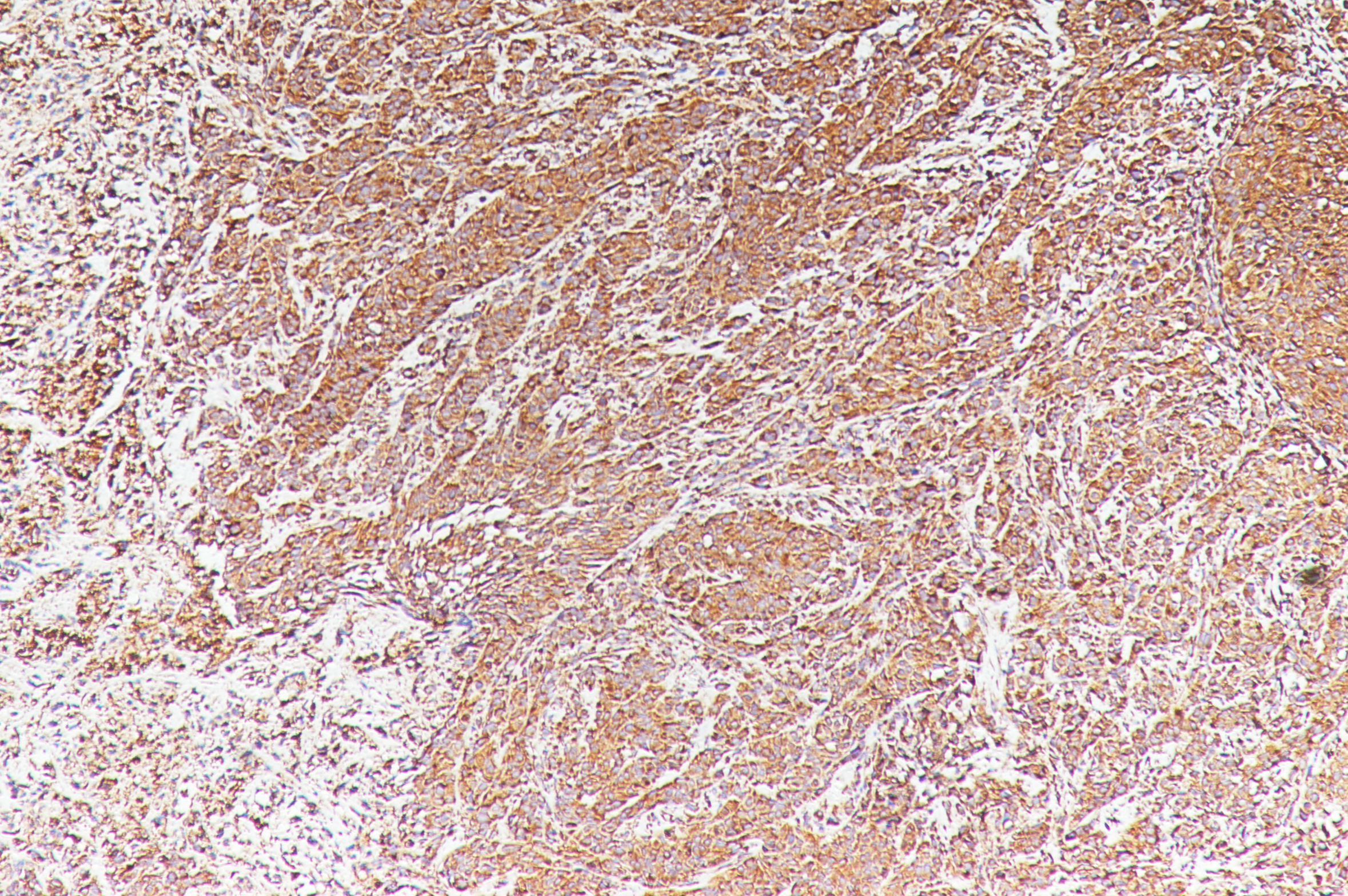

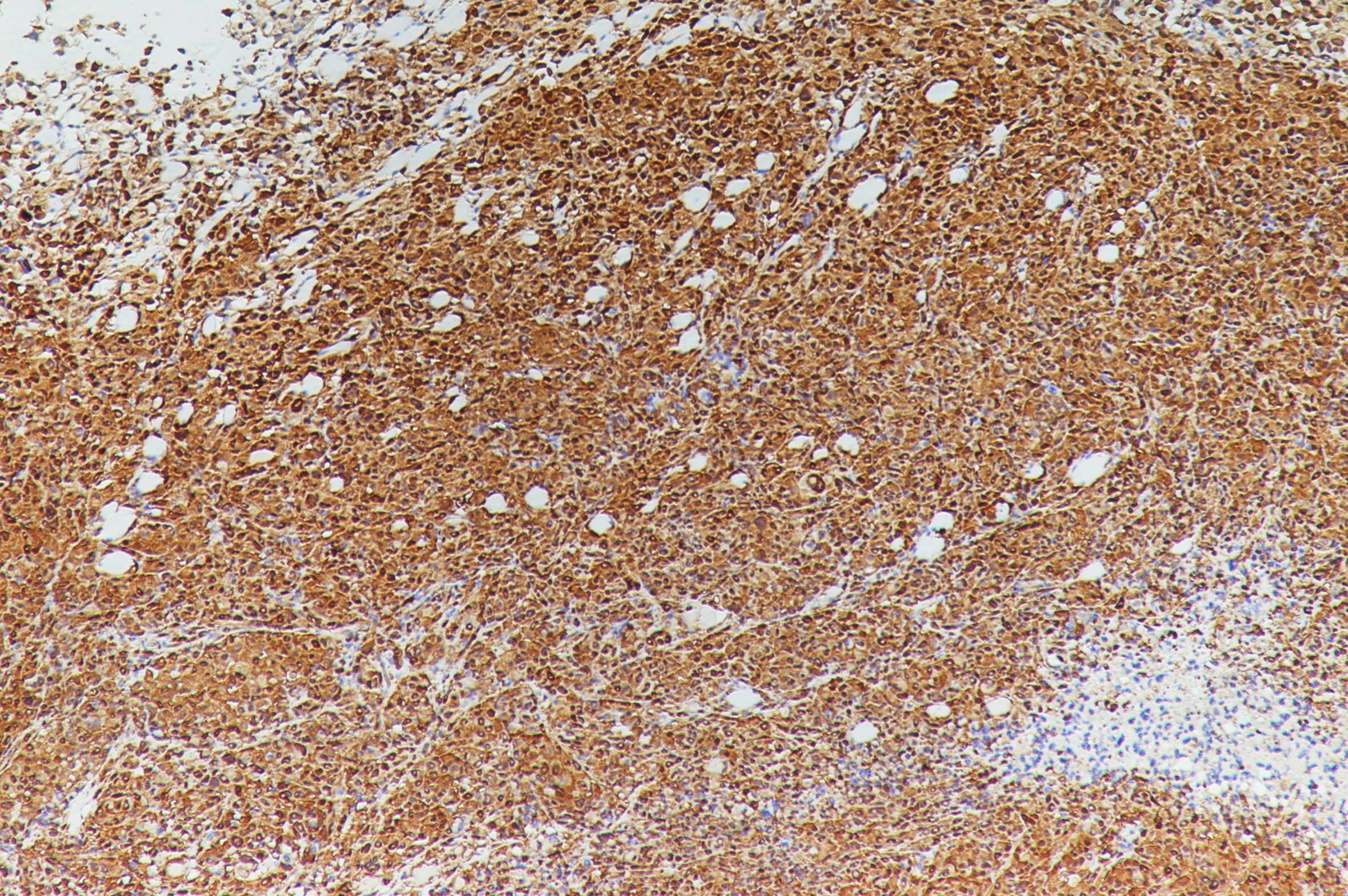

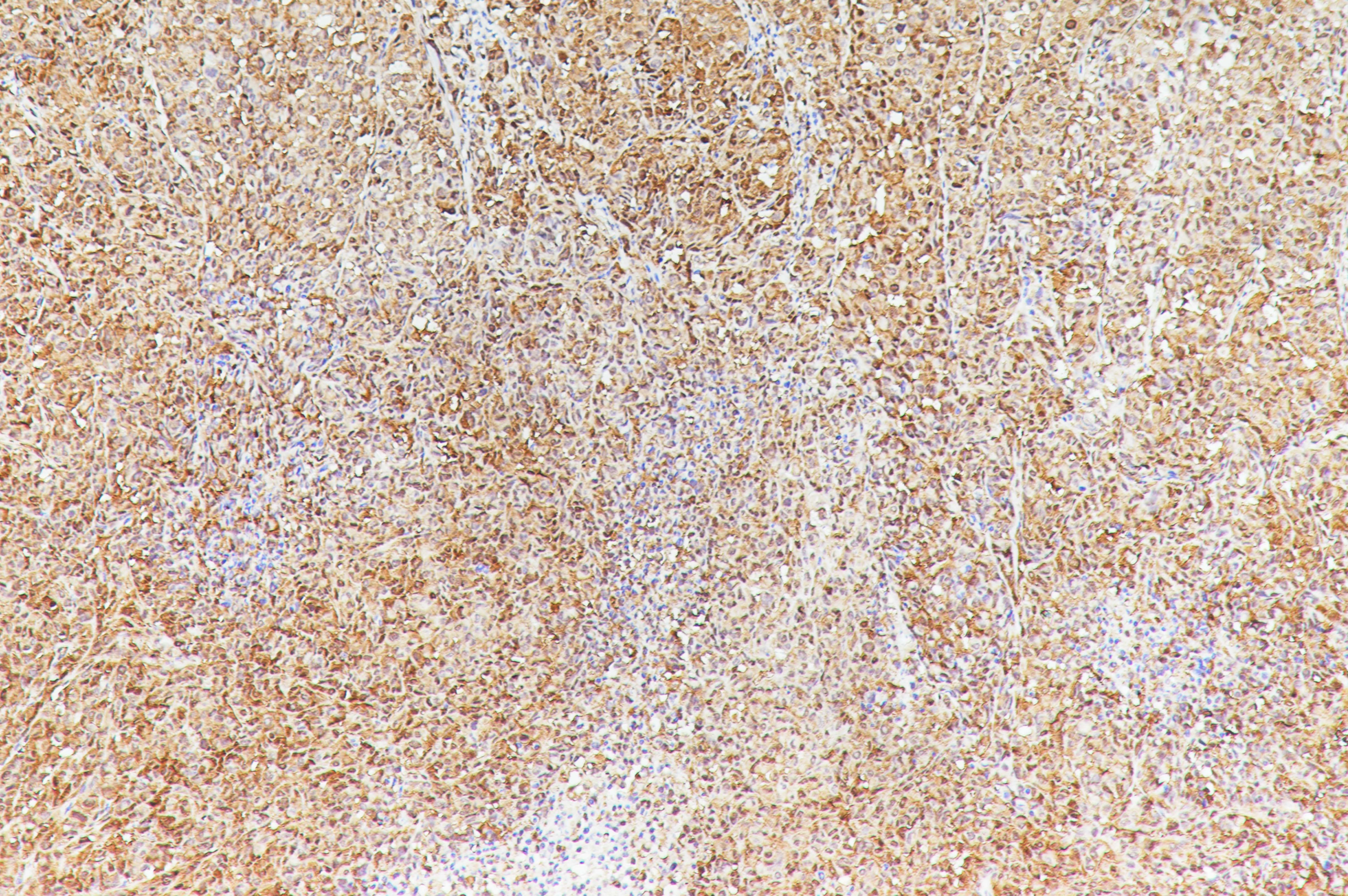

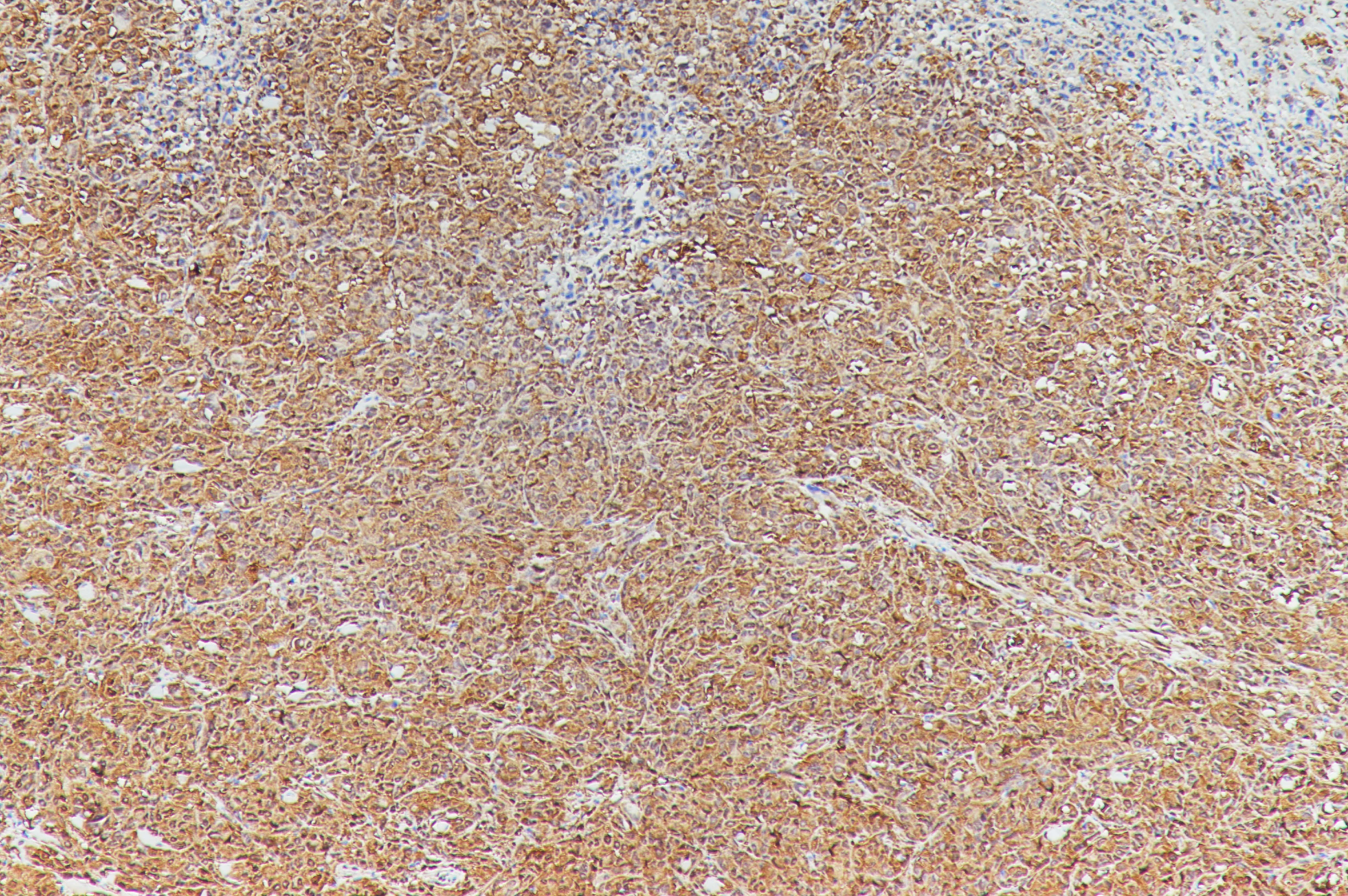

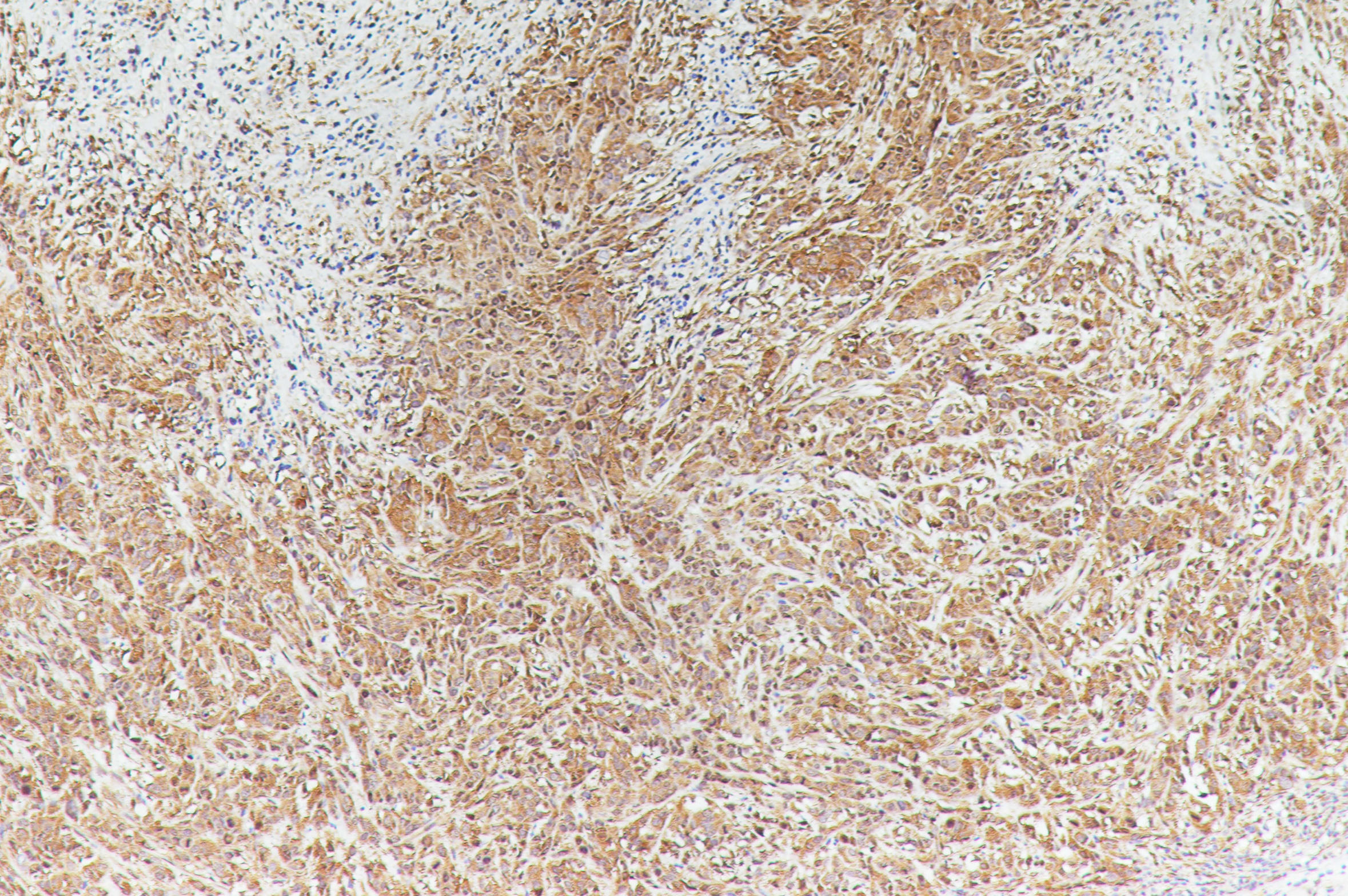

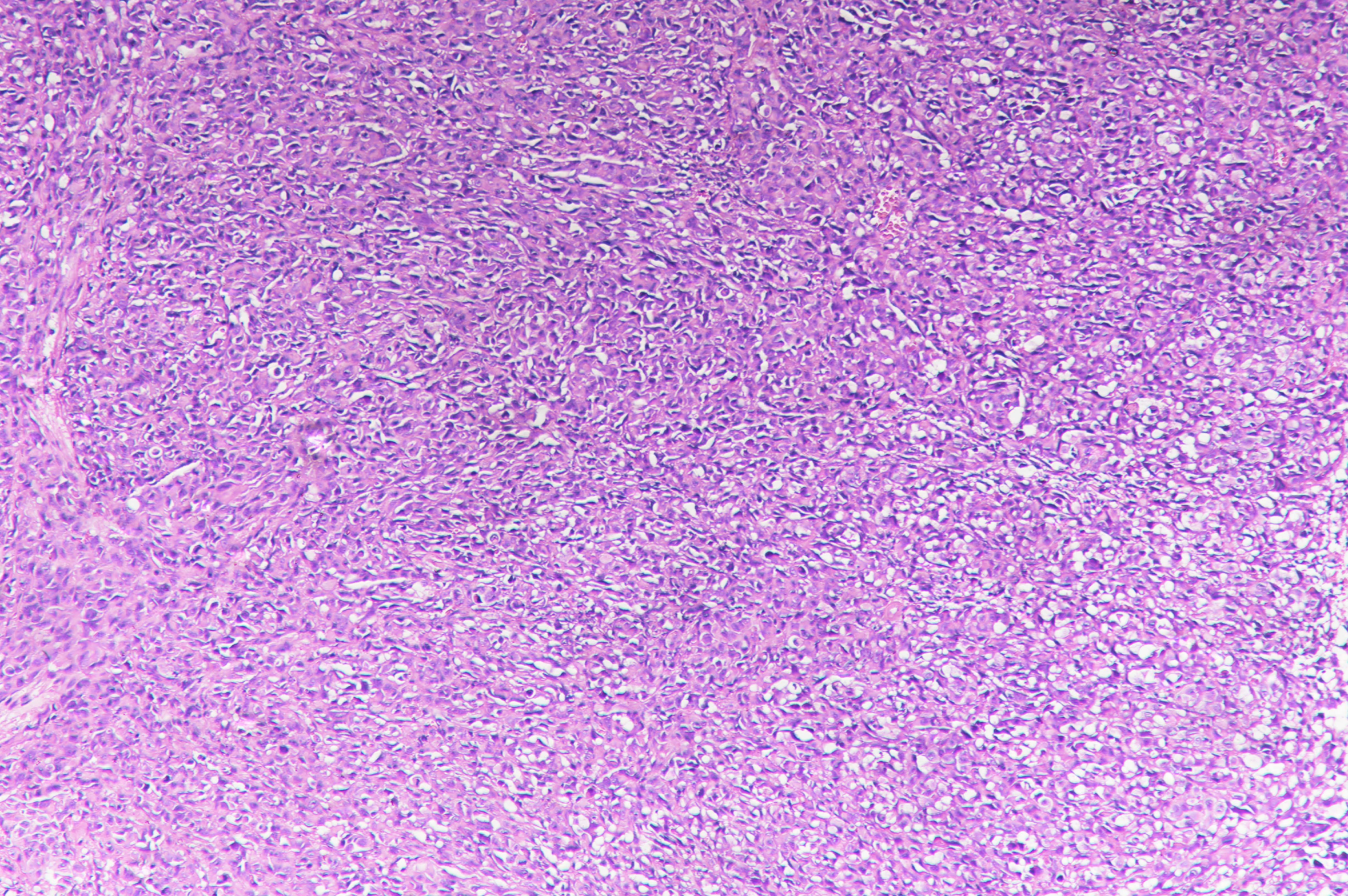

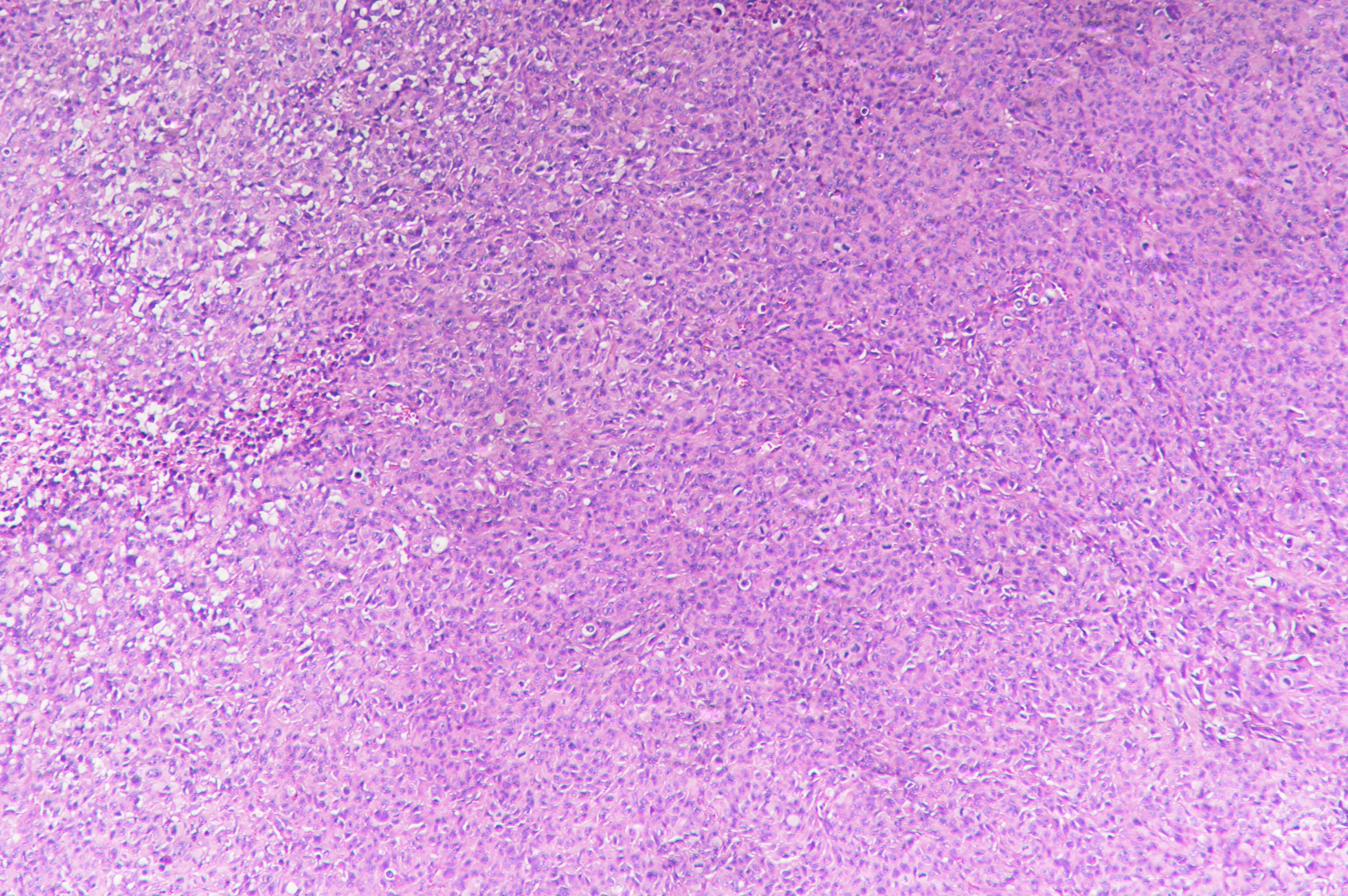

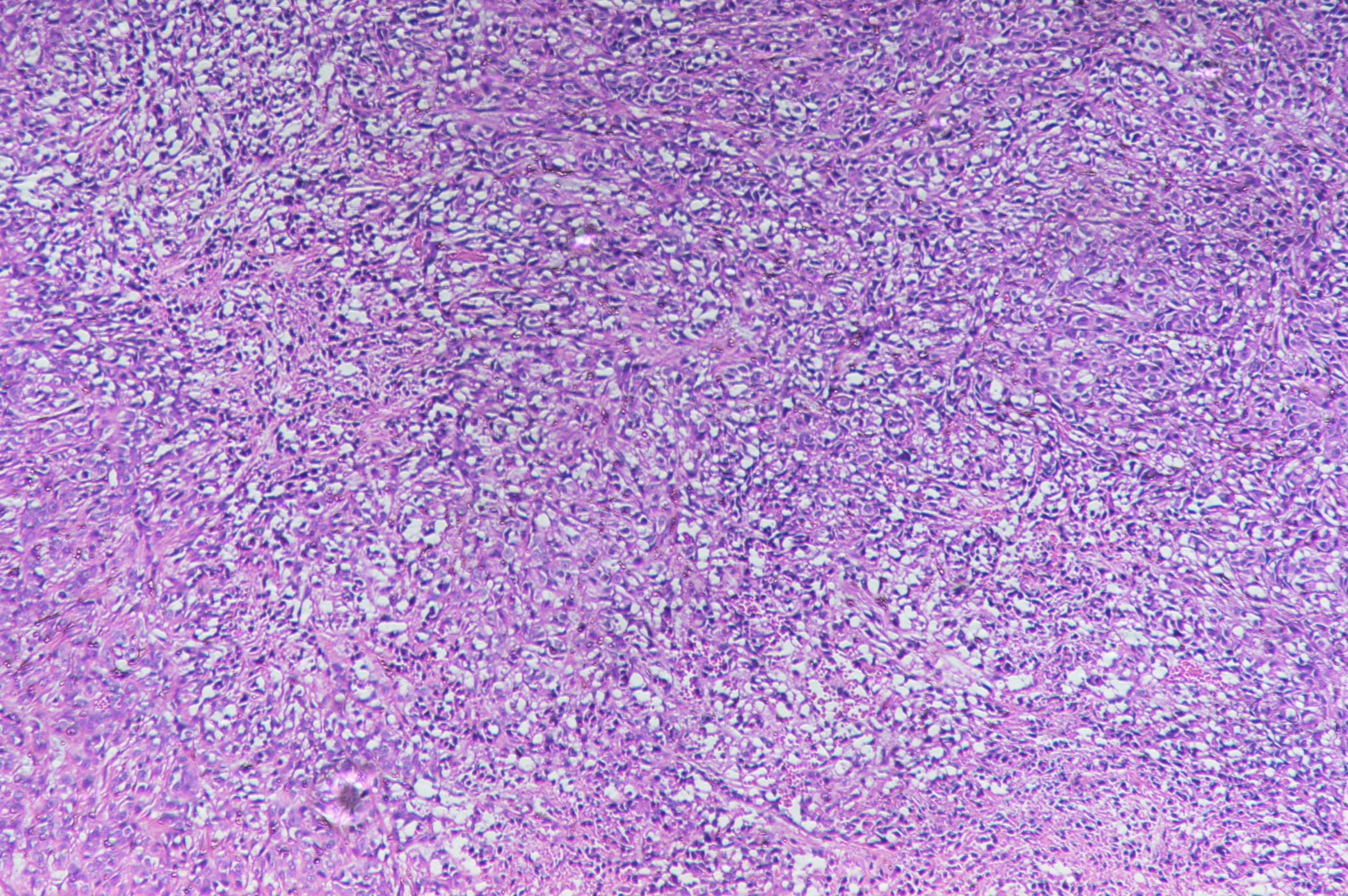

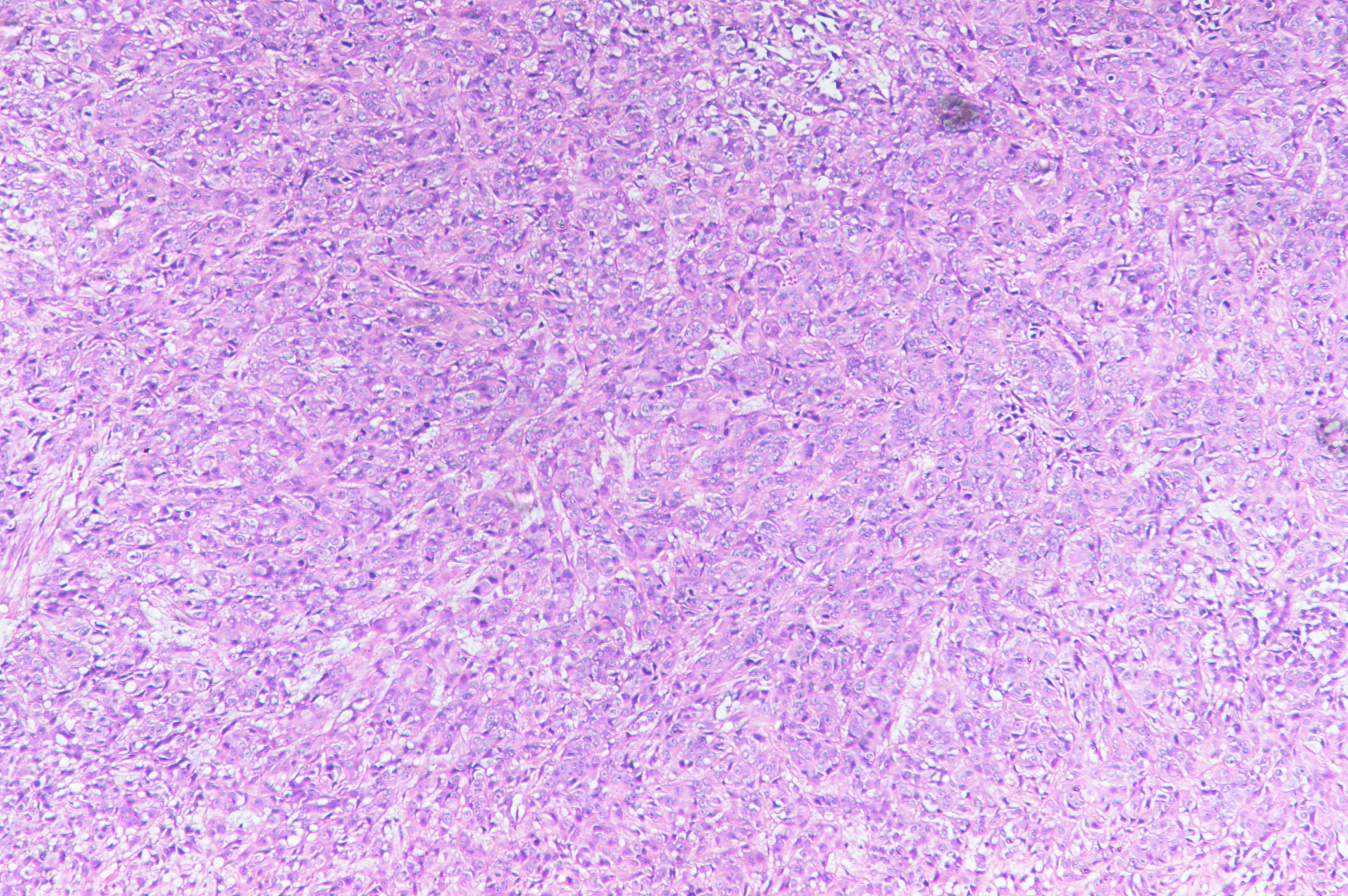





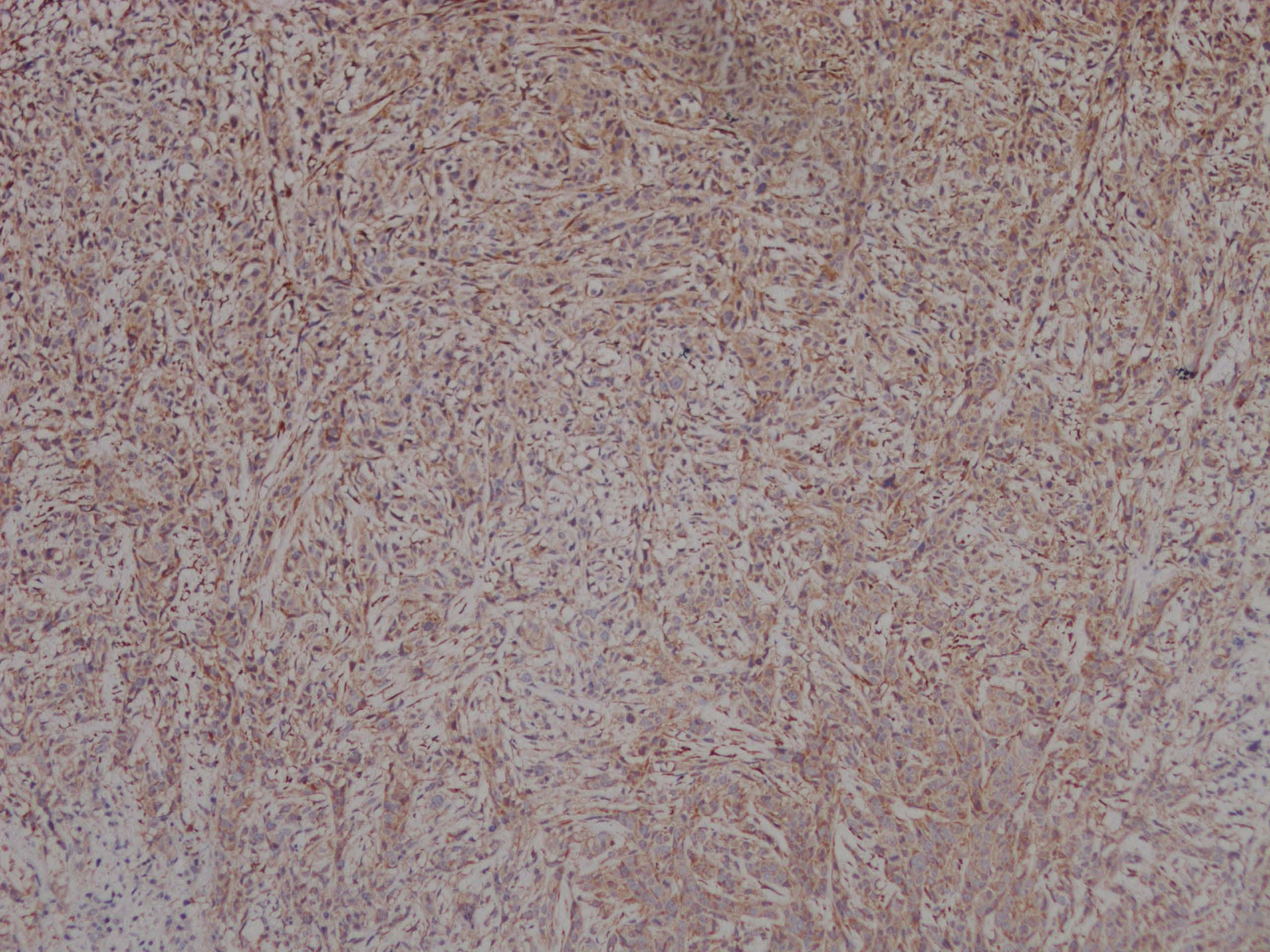

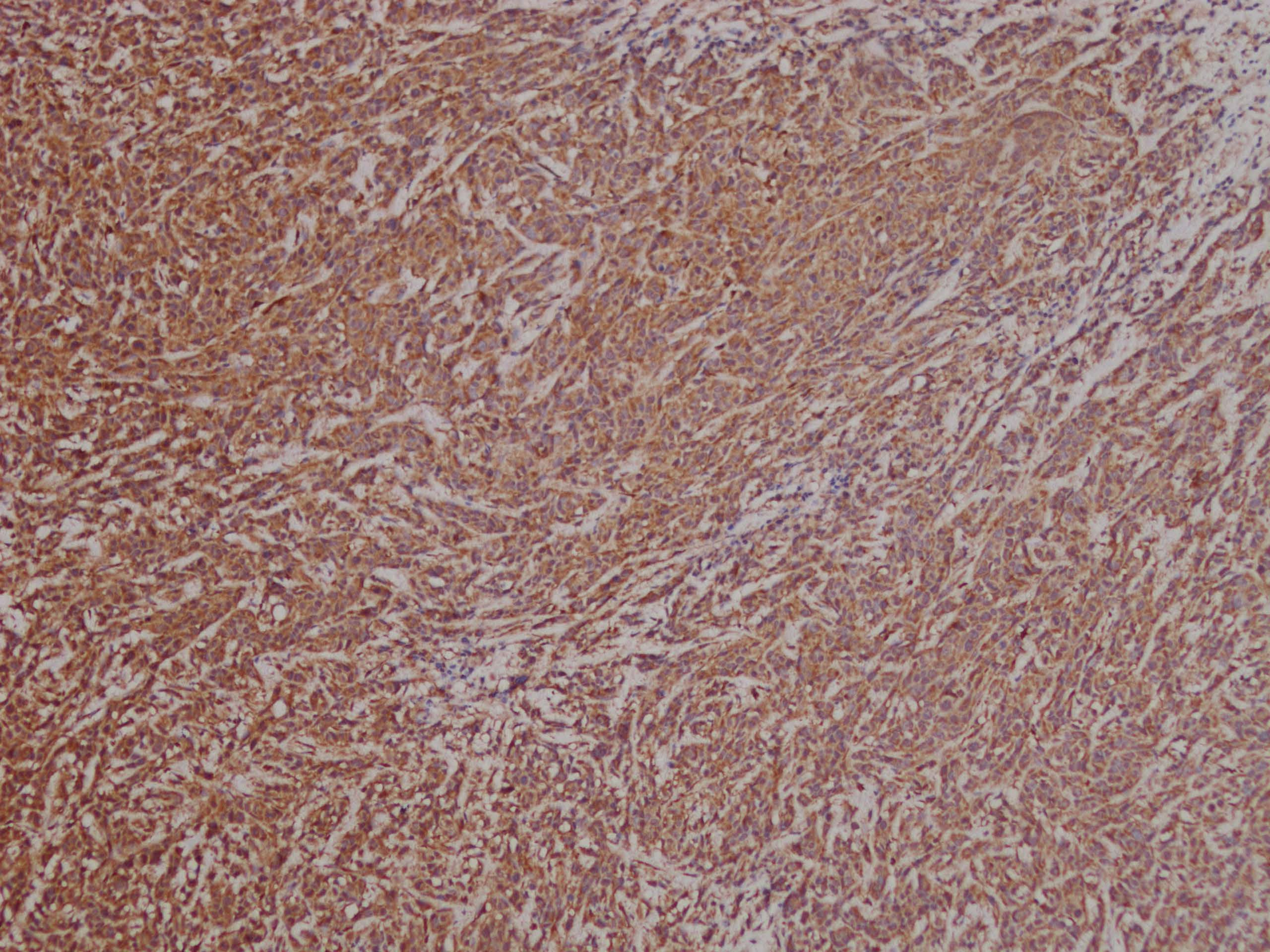

Supplement: Supplementary file 16 — Original Data File [file 41420_2022_968_MOESM16_ESM.pdf]
