# Supplementary material for: A positive feedback loop: RAD18-YAP-TGF-β between triple-negative breast cancer and macrophages regulates cancer stemness and progression
Source: Cell Death Discov. 2022 Apr 12;8:196. doi: 10.1038/s41420-022-00968-9 (PMC9005530; doi:10.1038/s41420-022-00968-9)

Figure.2I-CD44 (MDA-MB-231)

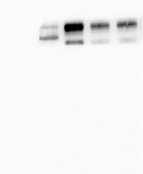

Figure.2I-CD44 (HCC-1806)

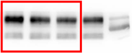

Figure.2A-GAPDH (MDA-MB-231)

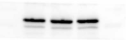

Figure.2A-GAPDH (HCC-1806)

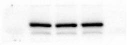

Figure.2I-GAPDH (MDA-MB-231)

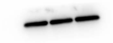

Figure.2I-GAPDH (HCC-1806)

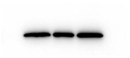

Figure.2I-Nanog (MDA-MB-231)

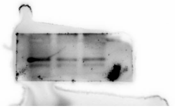

Figure.2I-Nanog (HCC-1806)

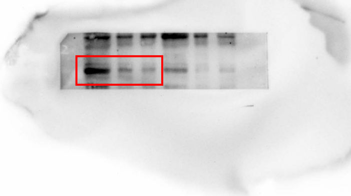

Figure.2I-OCT-4 (MDA-MB-231)

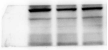

Figure.2I-OCT-4 (HCC-1806)

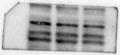

Figure.2A-RAD18 (MDA-MB-231)

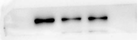

Figure.2A-RAD18 (HCC-1806)

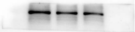

Figure.2I-RAD18 (MDA-MB-231)

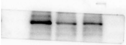

Figure.2I-RAD18 (HCC-1806)

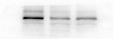

Figure.2I-SOX2 (MDA-MB-231)

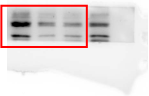

Figure.2I-SOX2 (HCC-1806)

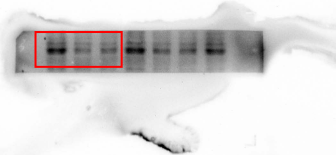

Supplement: Supplementary file 12 — Supplementary Figure 2 [file 41420_2022_968_MOESM12_ESM.pdf]
