# Supplementary material for: A positive feedback loop: RAD18-YAP-TGF-β between triple-negative breast cancer and macrophages regulates cancer stemness and progression
Source: Cell Death Discov. 2022 Apr 12;8:196. doi: 10.1038/s41420-022-00968-9 (PMC9005530; doi:10.1038/s41420-022-00968-9)

Figure.3F-CD44 (MDA-MB-231)

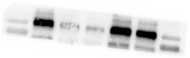

Figure.3F-CD44 (HCC-1806)

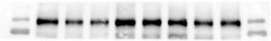

Figure.3C-GAPDH (MDA-MB-231)

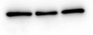

Figure.3C-GAPDH (HCC-1806)

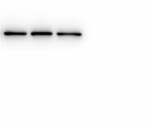

Figure.3C-GAPDH

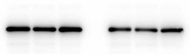

Figure.3F-GAPDH (MDA-MB-231)

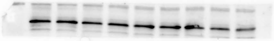

Figure.3F-GAPDH (HCC-1806)

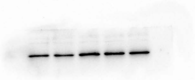

Figure.3C- Histone H3

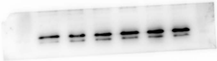

Figure.3C-LATS1

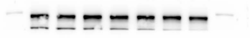

Figure.3C-MST1 (MDA-MB-231)

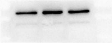

Figure.3C-MST1 (HCC-1806)

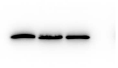

Figure.3C-MST2 (MDA-MB-231)

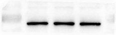

Figure.3C-MST2 (HCC-1806)

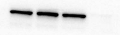

Figure.3F-NANOG (MDA-MB-231)

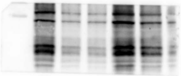

Figure.3F-NANOG (HCC-1806)

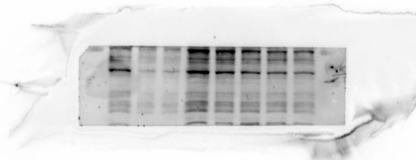

Figure.3F-OCT4 (MDA-MB-231)

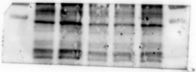

Figure.3F-OCT4 (HCC-1806)

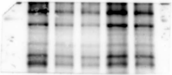

Figure.3C-p-LATS1 (MDA-MB-231)

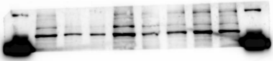

Figure.3C-p-LATS1 (HCC-1806)

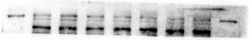

Figure.3C-p-YAP

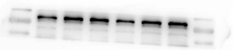

Figure.3C-RAD18 (MDA-MB-231)

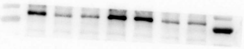

Figure.3C-RAD18 (HCC-1806)

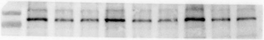

Figure.3F-RAD18 (MDA-MB-231)

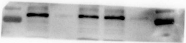

Figure.3F-RAD18 (HCC-1806)

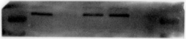

Figure.3F-SOX2 (MDA-MB-231)

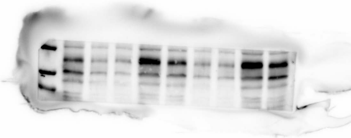

Figure.3F-SOX2 (HCC-1806)

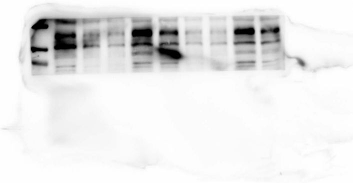

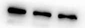

Figure.3C-YAP(cytoplasm) (MDA-MB-231)

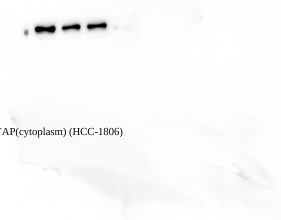

Figure.3C-YAP(cytoplasm) (HCC-1806)

Figure.3C-YAP(nucleus) (MDA-MB-231)

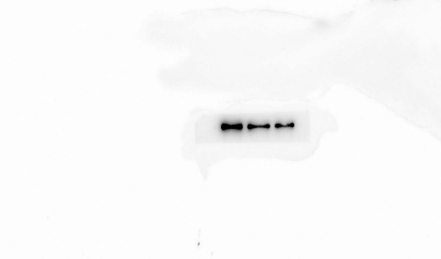

Figure.3C-YAP(nucleus) (HCC-1806)

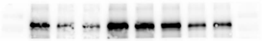

Figure.3C-YAP (MDA-MB-231)

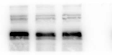

Figure.3C-YAP (HCC-1806)

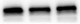

Figure.3F-YAP (MDA-MB-231)

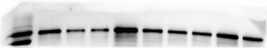

Figure.3F-YAP (HCC-1806)

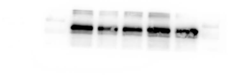

Supplement: Supplementary file 13 — Supplementary Figure 3 [file 41420_2022_968_MOESM13_ESM.pdf]
