# Supplementary material for: A positive feedback loop: RAD18-YAP-TGF-β between triple-negative breast cancer and macrophages regulates cancer stemness and progression
Source: Cell Death Discov. 2022 Apr 12;8:196. doi: 10.1038/s41420-022-00968-9 (PMC9005530; doi:10.1038/s41420-022-00968-9)

Figure.5E-CD163

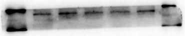

Figure.5E-c-Jun

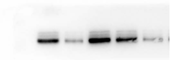

Figure.5E-GAPDH

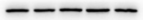

Figure.5E-JNK

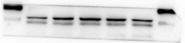

Figure.5E-NF-kBp65

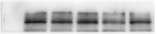

Figure.5E-NF-kBp-p65

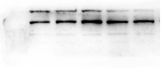

Figure.5E-p-JNK

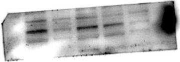

Figure.5E-PPARD

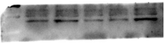

Figure.5H-CD44

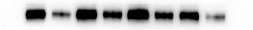

Figure.5H-GAPDH

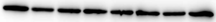

Figure.5H-Nanog

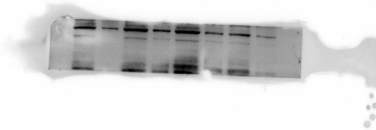

Figure.5H-OCT-4

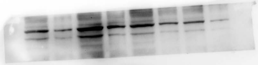

Figure.5H-RAD18

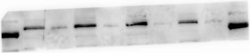

Figure.5H-SOX2

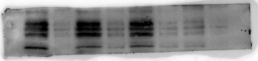

Supplement: Supplementary file 15 — Supplementary Figure 5 [file 41420_2022_968_MOESM15_ESM.pdf]
