# Supplementary material for: A positive feedback loop: RAD18-YAP-TGF-β between triple-negative breast cancer and macrophages regulates cancer stemness and progression
Source: Cell Death Discov. 2022 Apr 12;8:196. doi: 10.1038/s41420-022-00968-9 (PMC9005530; doi:10.1038/s41420-022-00968-9)
Supplement: Supplementary file 17 — Original Data File [file 41420_2022_968_MOESM17_ESM.pdf]

BD FACSDiva 8.0.2

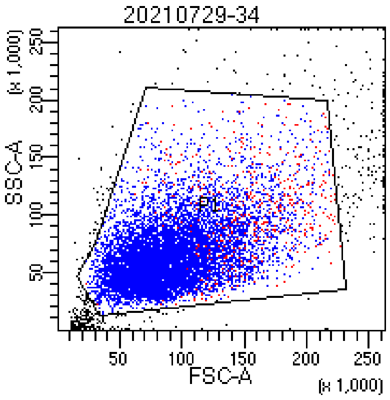

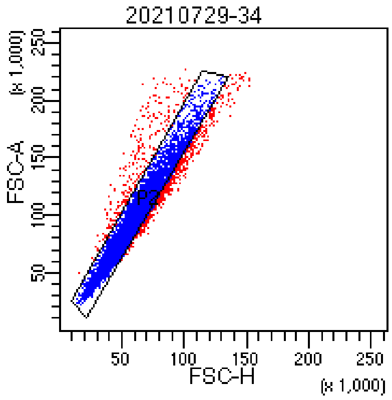

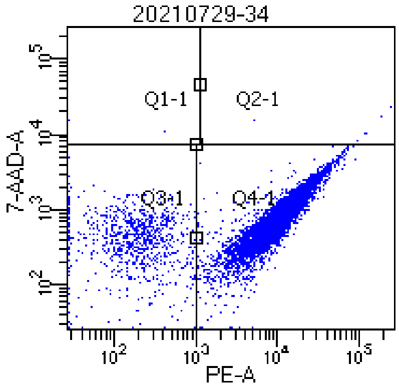

Tube: 34

Population	#Events	%Parent	%Total
All Events	10,000	####	100.0
P1	9,458	94.6	94.6
P2	8,607	91.0	86.1
Q1	5	0.1	0.0
Q2	276	3.2	2.8
Q3	859	10.0	8.6
Q4	7,467	86.8	74.7
Q1-1	2	0.0	0.0
Q2-1	8	0.1	0.1
Q3-1	797	9.3	8.0
Q4-1	7,800	90.6	78.0

BD FACSDiva 8.0.2

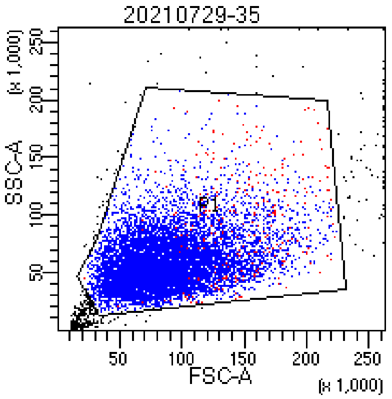

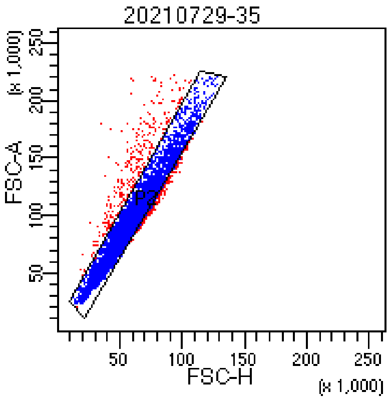

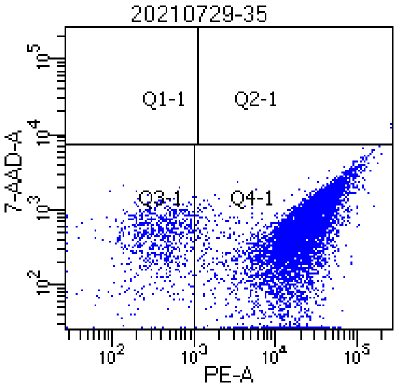

Tube: 35

Population	#Events	%Parent	%Total
All Events	10,000	####	100.0
P1	9,568	95.7	95.7
P2	9,076	94.9	90.8
Q1	0	0.0	0.0
Q2	130	1.4	1.3
Q3	787	8.7	7.9
Q4	8,159	89.9	81.6
Q1-1	0	0.0	0.0
Q2-1	2	0.0	0.0
Q3-1	708	7.8	7.1
Q4-1	8,366	92.2	83.7

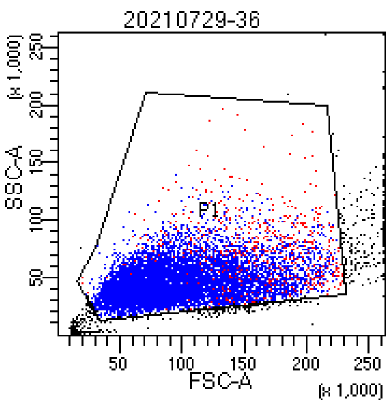

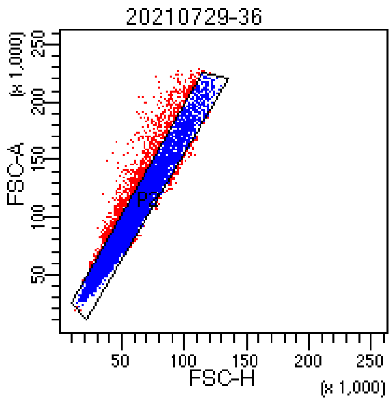

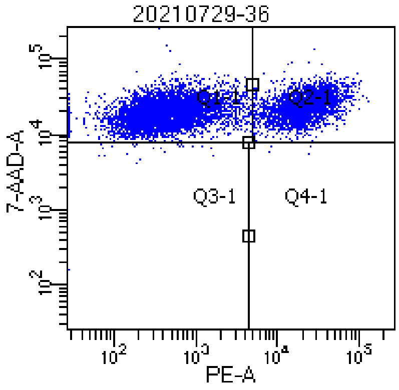

Tube: 36

Population	#Events	%Parent	%Total
All Events	10,000	####	100.0
P1	9,335	93.4	93.4
P2	8,075	86.5	80.8
Q1	4,845	60.0	48.4
Q2	3,229	40.0	32.3
Q3	1	0.0	0.0
Q4	0	0.0	0.0
Q1-1	5,242	64.9	52.4
Q2-1	2,803	34.7	28.0
Q3-1	26	0.3	0.3
Q4-1	4	0.0	0.0

BD FACSDiva 8.0.2

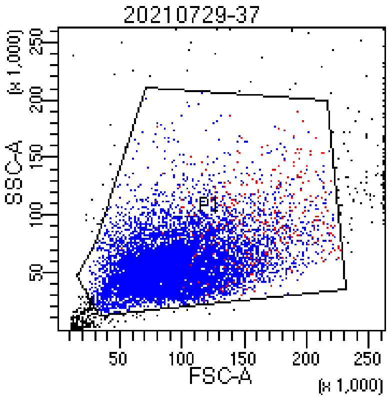

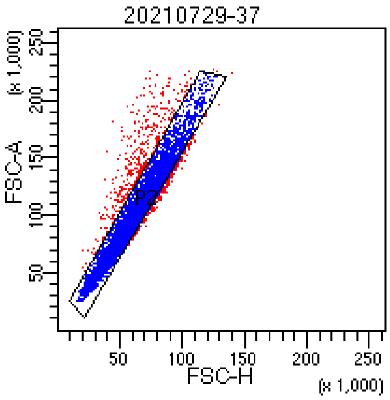

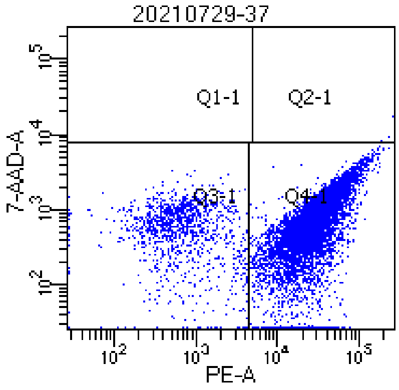

Tube: 37

Population	#Events	%Parent	%Total
All Events	10,000	####	100.0
P1	9,526	95.3	95.3
P2	8,849	92.9	88.5
Q1	4	0.0	0.0
Q2	319	3.6	3.2
Q3	959	10.8	9.6
Q4	7,567	85.5	75.7
Q1-1	0	0.0	0.0
Q2-1	4	0.0	0.0
Q3-1	1,155	13.1	11.6
Q4-1	7,690	86.9	76.9

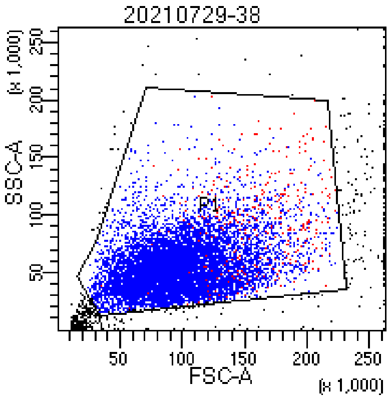

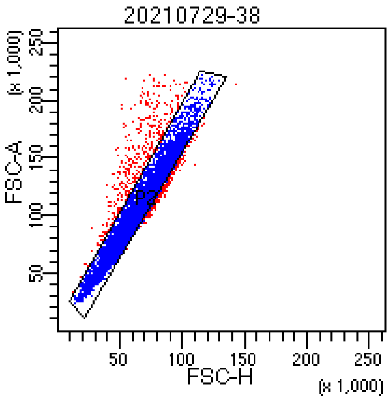

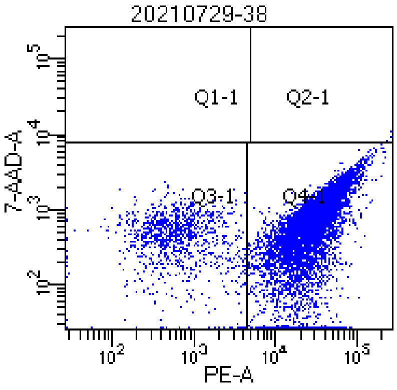

Tube: 38

Population	#Events	%Parent	%Total
All Events	10,000	####	100.0
P1	9,350	93.5	93.5
P2	8,700	93.0	87.0
Q1	0	0.0	0.0
Q2	265	3.0	2.6
Q3	893	10.3	8.9
Q4	7,542	86.7	75.4
Q1-1	0	0.0	0.0
Q2-1	4	0.0	0.0
Q3-1	1,056	12.1	10.6
Q4-1	7,640	87.8	76.4

BD FACSDiva 8.0.2

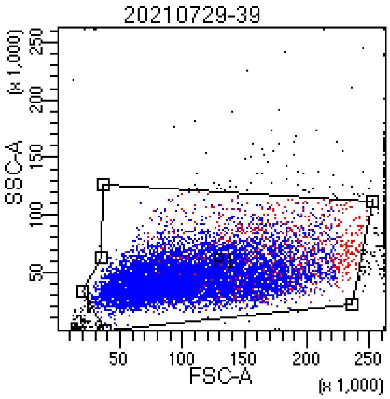

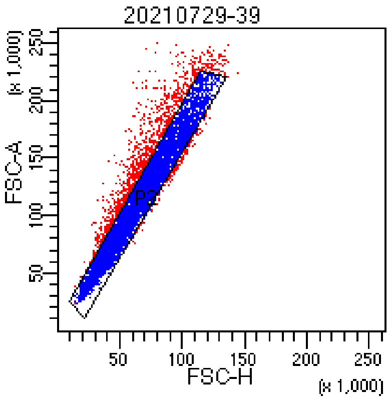

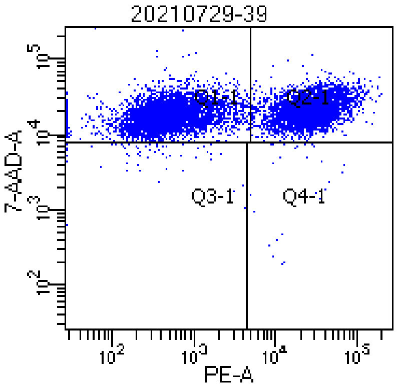

Tube: 39

Population	#Events	%Parent	%Total
All Events	10,000	####	100.0
P1	9,529	95.3	95.3
P2	8,176	85.8	81.8
Q1	3,867	47.3	38.7
Q2	4,292	52.5	42.9
Q3	2	0.0	0.0
Q4	15	0.2	0.2
Q1-1	4,218	51.6	42.2
Q2-1	3,870	47.3	38.7
Q3-1	60	0.7	0.6
Q4-1	28	0.3	0.3

BD FACSDiva 8.0.2

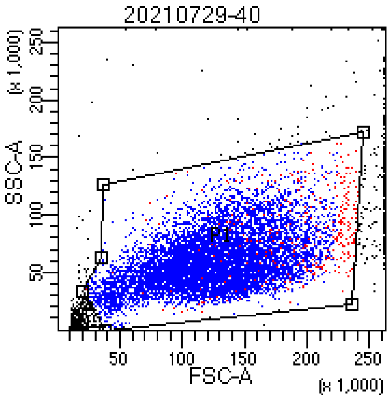

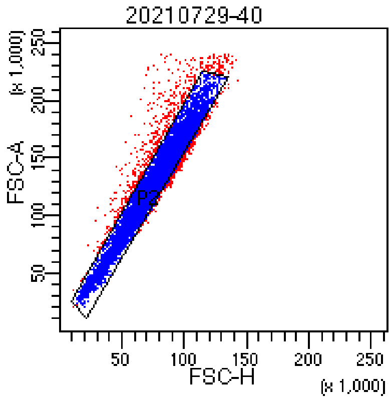

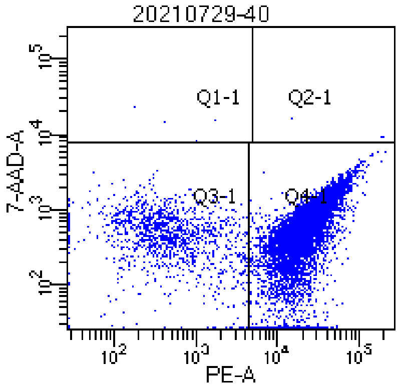

Tube: 40

Population	#Events	%Parent	%Total
All Events	10,000	####	100.0
P1	9,035	90.4	90.4
P2	8,142	90.1	81.4
Q1	6	0.1	0.1
Q2	87	1.1	0.9
Q3	1,023	12.6	10.2
Q4	7,026	86.3	70.3
Q1-1	4	0.0	0.0
Q2-1	3	0.0	0.0
Q3-1	1,218	15.0	12.2
Q4-1	6,917	85.0	69.2

BD FACSDiva 8.0.2

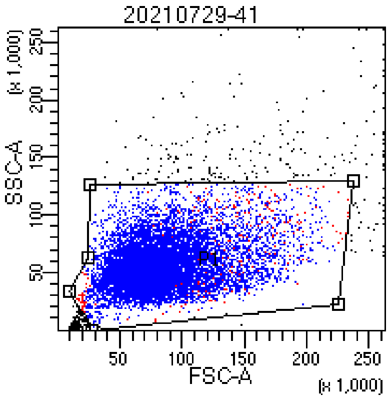

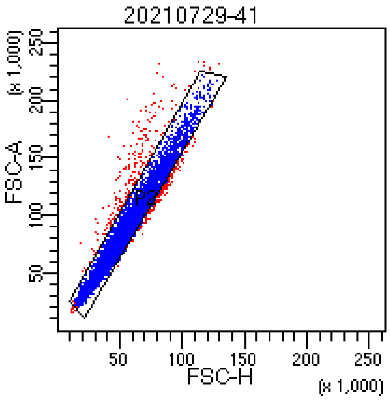

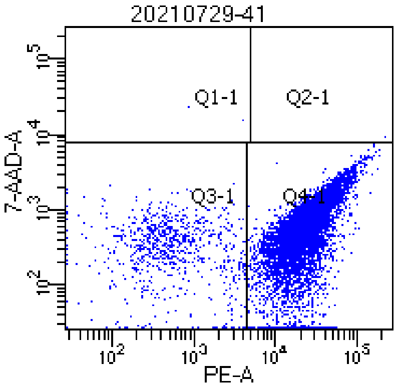

Tube: 41

Population	#Events	%Parent	%Total
All Events	10,000	####	100.0
P1	9,594	95.9	95.9
P2	9,135	95.2	91.4
Q1	1	0.0	0.0
Q2	171	1.9	1.7
Q3	630	6.9	6.3
Q4	8,333	91.2	83.3
Q1-1	2	0.0	0.0
Q2-1	1	0.0	0.0
Q3-1	773	8.5	7.7
Q4-1	8,359	91.5	83.6

BD FACSDiva 8.0.2

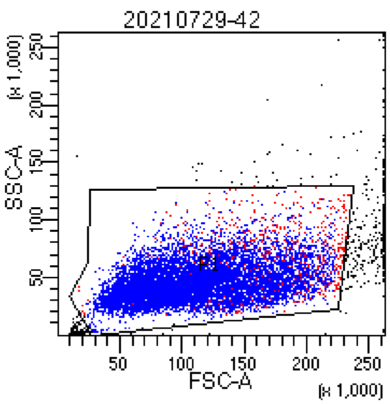

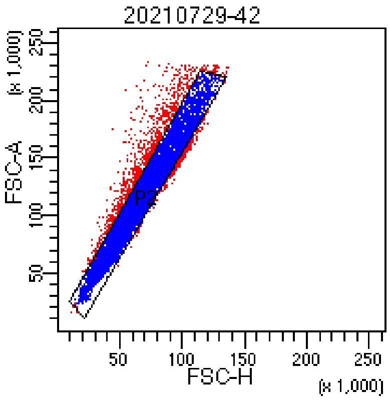

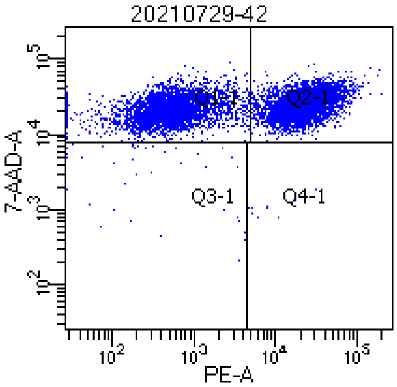

Tube: 42

Population	#Events	%Parent	%Total
All Events	10,000	####	100.0
P1	9,453	94.5	94.5
P2	8,240	87.2	82.4
Q1	3,470	42.1	34.7
Q2	4,745	57.6	47.4
Q3	7	0.1	0.1
Q4	18	0.2	0.2
Q1-1	3,792	46.0	37.9
Q2-1	4,398	53.4	44.0
Q3-1	38	0.5	0.4
Q4-1	12	0.1	0.1

BD FACSDiva 8.0.2

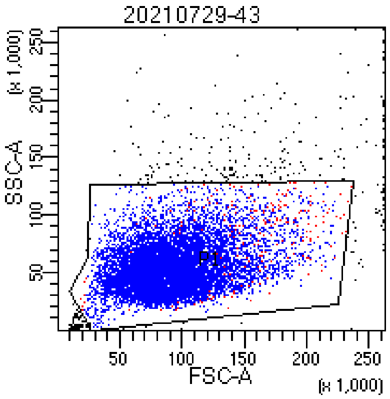

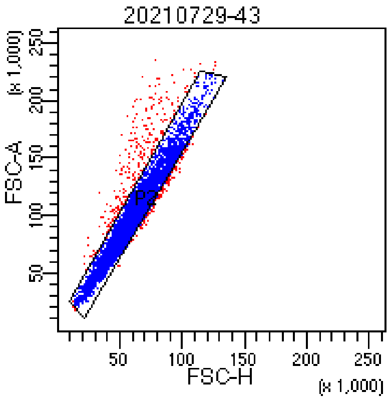

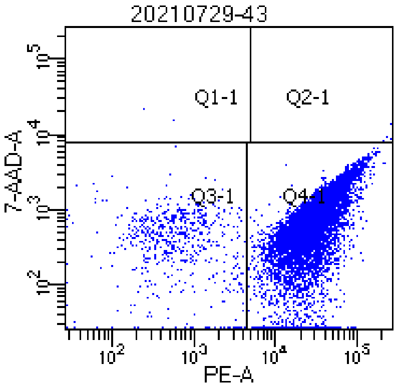

Tube: 43

Population	#Events	%Parent	%Total
All Events	10,000	####	100.0
P1	9,623	96.2	96.2
P2	9,176	95.4	91.8
Q1	5	0.1	0.0
Q2	306	3.3	3.1
Q3	492	5.4	4.9
Q4	8,373	91.2	83.7
Q1-1	2	0.0	0.0
Q2-1	5	0.1	0.0
Q3-1	601	6.5	6.0
Q4-1	8,568	93.4	85.7

BD FACSDiva 8.0.2

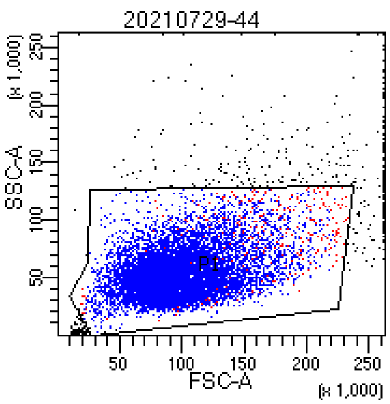

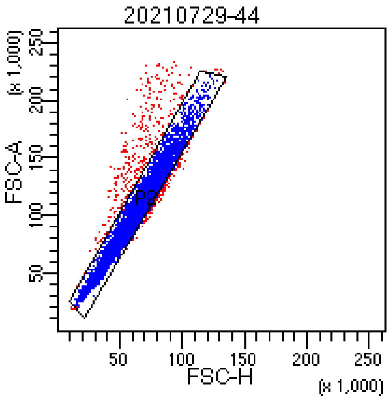

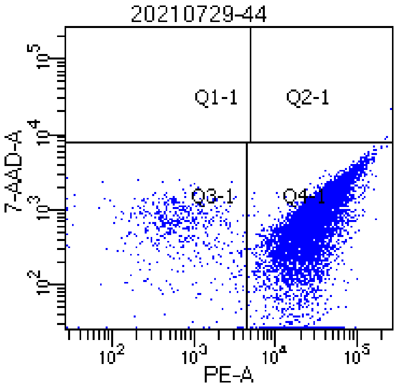

Tube: 44

Population	#Events	%Parent	%Total
All Events	10,000	####	100.0
P1	9,527	95.3	95.3
P2	8,978	94.2	89.8
Q1	0	0.0	0.0
Q2	358	4.0	3.6
Q3	450	5.0	4.5
Q4	8,170	91.0	81.7
Q1-1	0	0.0	0.0
Q2-1	5	0.1	0.0
Q3-1	564	6.3	5.6
Q4-1	8,409	93.7	84.1

BD FACSDiva 8.0.2

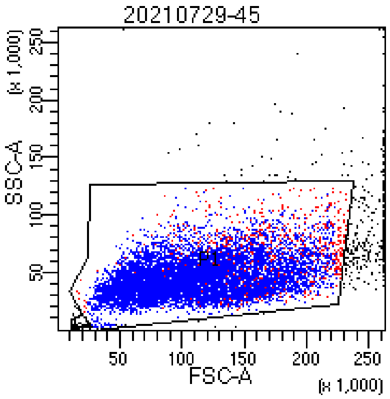

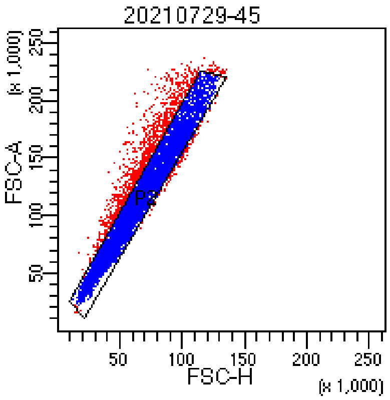

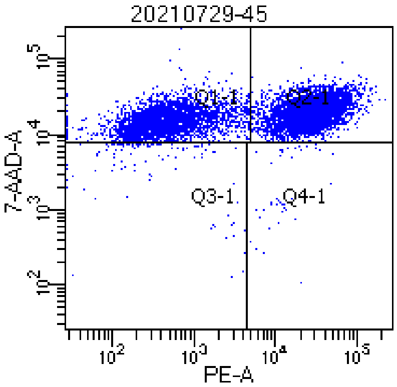

Tube: 45

Population	#Events	%Parent	%Total
All Events	10,000	####	100.0
P1	9,446	94.5	94.5
P2	8,083	85.6	80.8
Q1	3,141	38.9	31.4
Q2	4,897	60.6	49.0
Q3	7	0.1	0.1
Q4	38	0.5	0.4
Q1-1	3,391	42.0	33.9
Q2-1	4,533	56.1	45.3
Q3-1	119	1.5	1.2
Q4-1	40	0.5	0.4
