# Supplementary material for: A positive feedback loop: RAD18-YAP-TGF-β between triple-negative breast cancer and macrophages regulates cancer stemness and progression
Source: Cell Death Discov. 2022 Apr 12;8:196. doi: 10.1038/s41420-022-00968-9 (PMC9005530; doi:10.1038/s41420-022-00968-9)

BD FACSDiva 8.0.2

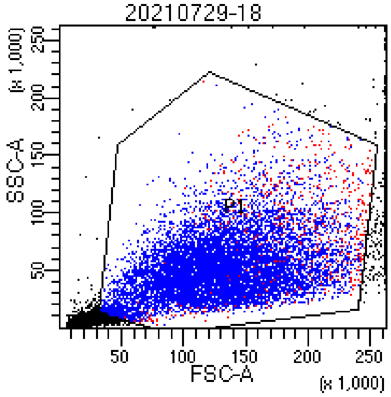

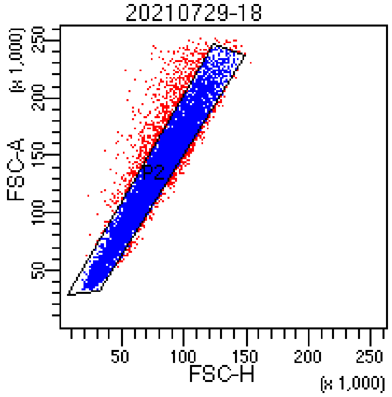

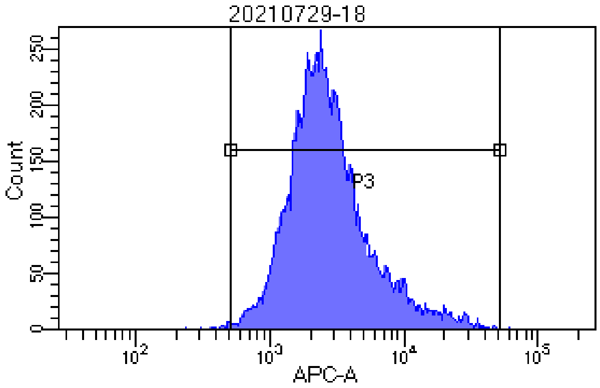

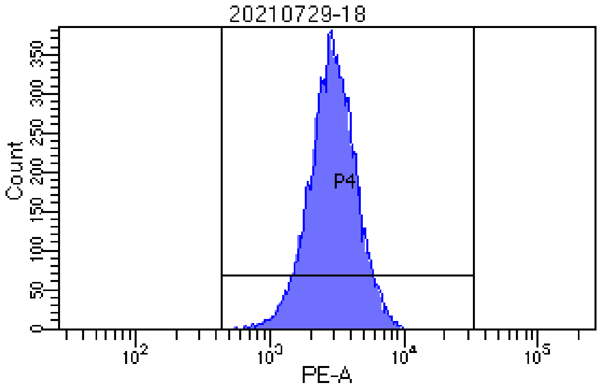

Experiment Name:	YXQ-1
Specimen Name:	20210729
Tube Name:	18
Record Date:	Jul 29, 2021 5:52:52 PM

Population	#Events	%Pare...	APC-A Geo M...	APC-A Mean	APC-A Median
P1	10,716	72.1	####	4,105	2,512
P2	9,622	89.8	2,693	3,940	2,420
P3	9,525	99.0	2,720	3,828	2,433
P4	9,609	99.9	2,695	3,941	2,422
Q1	1	0.0	11,658	11,658	11,658
Q2	568	5.9	18,768	21,192	17,179
Q3	0	0.0	####	####	####
Q4	9,053	94.1	2,384	2,857	2,320

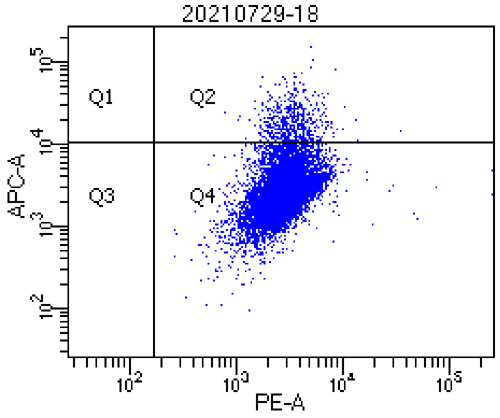

BD FACSDiva 8.0.2

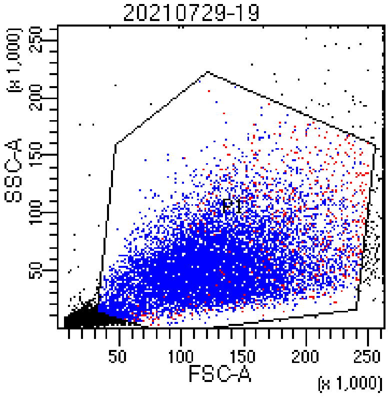

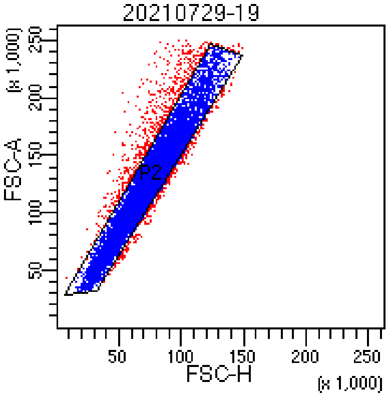

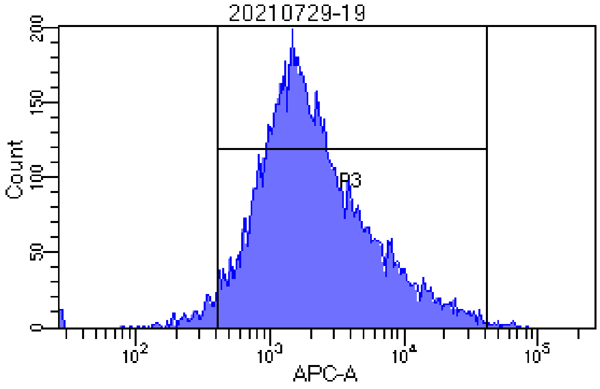

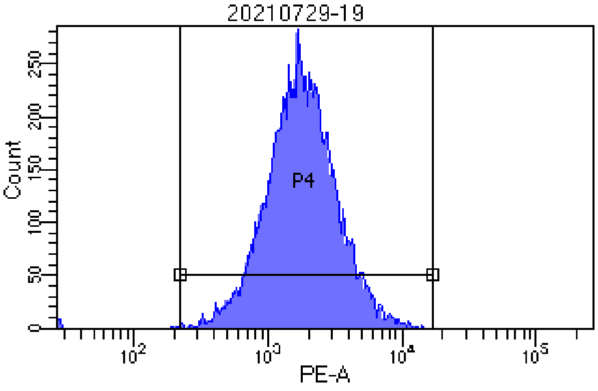

Experiment Name:	YXQ-1
Specimen Name:	20210729
Tube Name:	19
Record Date:	Jul 29, 2021 5:54:14 PM

Population	#Events	%Pare...	APC-A Geo M...	APC-A Mean	APC-A Median
P1	10,781	63.4	####	3,960	1,825
P2	9,811	91.0	####	3,930	1,774
P3	9,355	95.4	2,172	3,617	1,841
P4	9,746	99.3	####	3,944	1,782
Q1	0	0.0	####	####	####
Q2	748	7.6	19,589	22,876	17,247
Q3	37	0.4	####	487	337
Q4	9,026	92.0	####	2,374	1,641

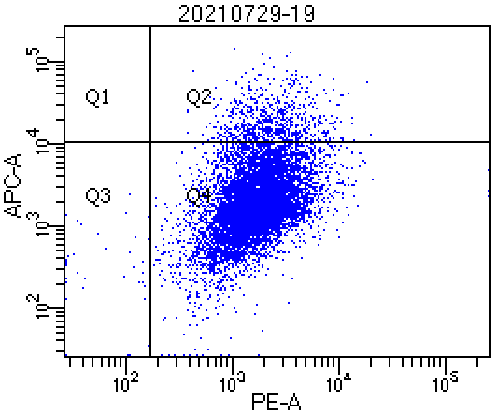

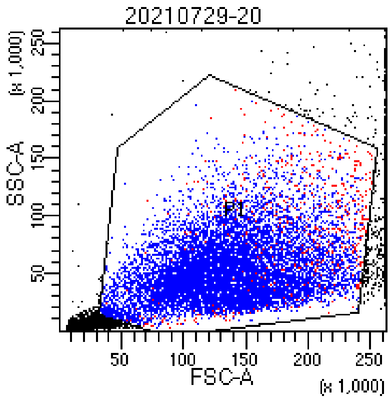

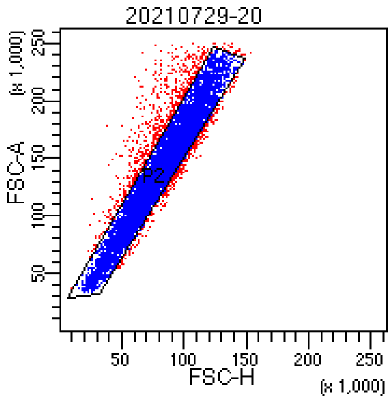

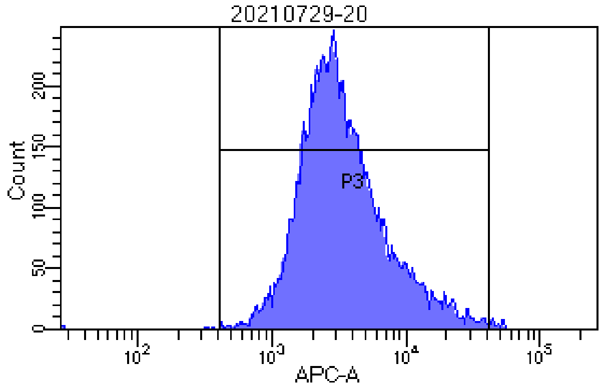

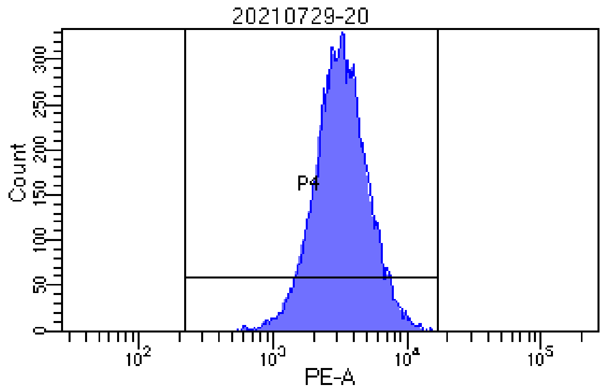

Experiment Name: YXQ-1
Specimen Name: 20210729
Tube Name: 20
Record Date: Jul 29, 2021 5:55:12 PM

Population	#Events	%Pare...	APC-A Geo M...	APC-A Mean	APC-A Median
P1	10,840	66.5	####	5,015	3,038
P2	9,729	89.8	####	4,837	2,921
P3	9,633	99.0	3,278	4,587	2,916
P4	9,701	99.7	####	4,839	2,921
Q1	0	0.0	####	####	####
Q2	889	9.1	17,991	20,068	16,301
Q3	8	0.1	####	2,033	1,258
Q4	8,832	90.8	####	3,306	2,730

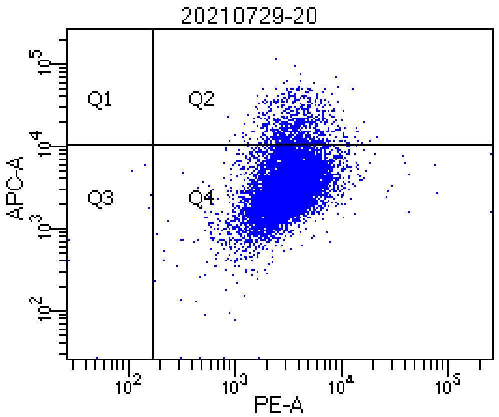

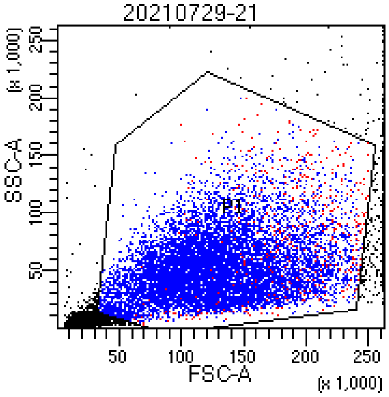

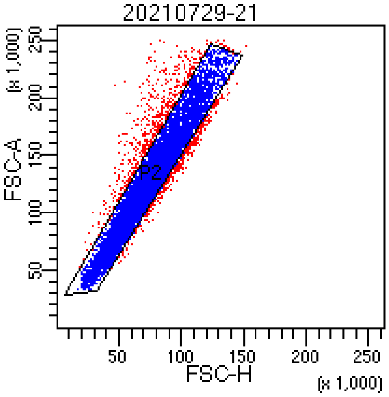

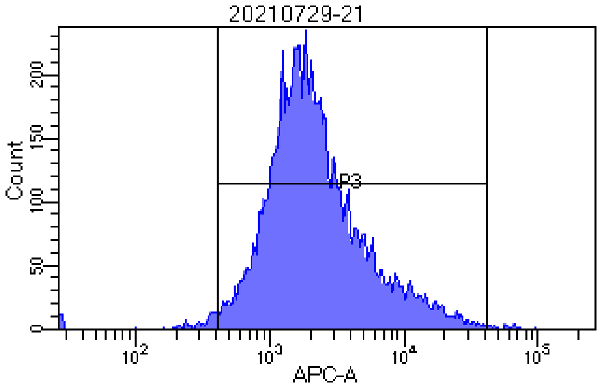

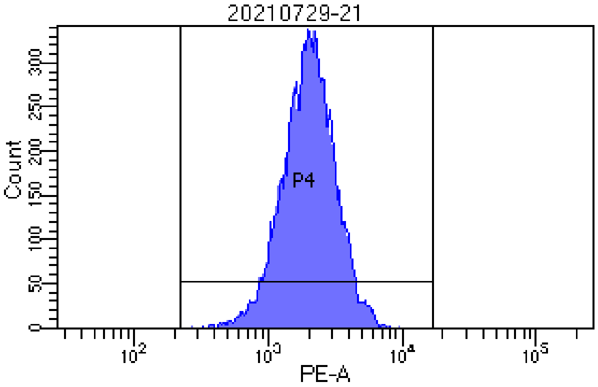

Experiment Name:	YXQ-1
Specimen Name:	20210729
Tube Name:	21
Record Date:	Jul 29, 2021 5:55:54 PM

Population	#Events	%Pare...	APC-A Geo M...	APC-A Mean	APC-A Median
P1	10,824	73.2	####	3,727	1,919
P2	9,812	90.7	####	3,650	1,859
P3	9,557	97.4	2,179	3,356	1,884
P4	9,790	99.8	####	3,654	1,862
Q1	1	0.0	10,928	10,928	10,928
Q2	654	6.7	18,639	21,659	16,460
Q3	9	0.1	####	1,048	639
Q4	9,148	93.2	####	2,364	1,760

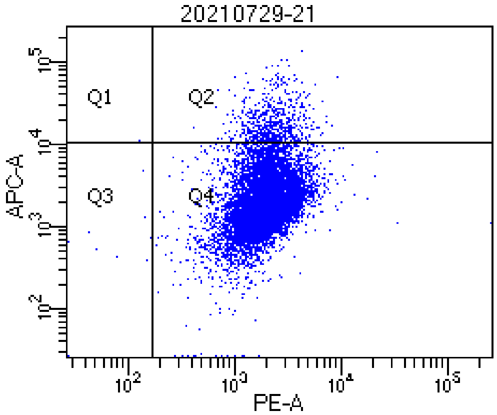

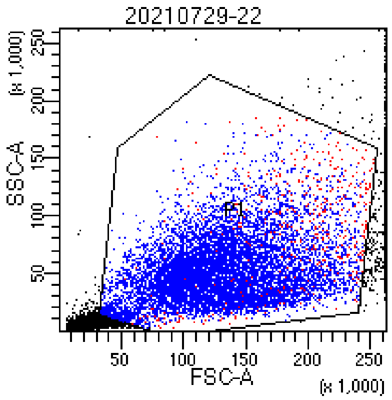

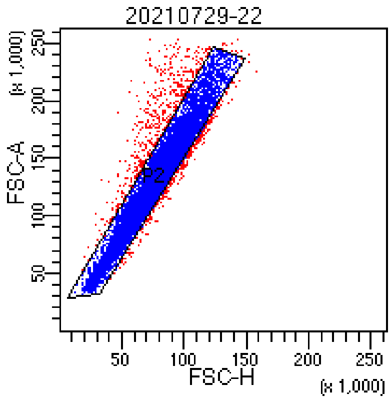

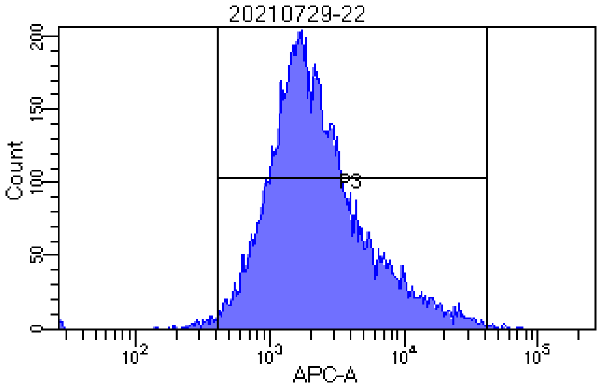

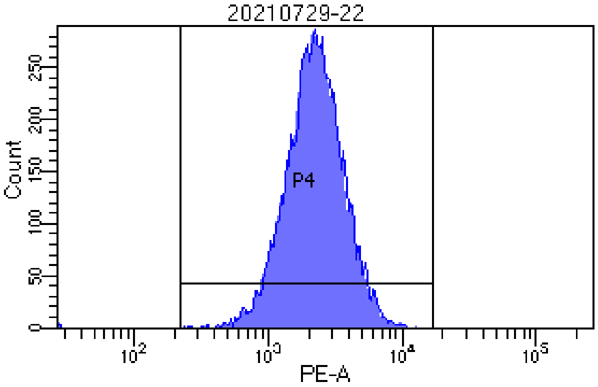

Experiment Name: YXQ-1
Specimen Name: 20210729
Tube Name: 22
Record Date: Jul 29, 2021 5:56:41 PM

Population	#Events	%Pare...	APC-A Geo M...	APC-A Mean	APC-A Median
P1	10,000	68.0	####	3,790	1,962
P2	9,143	91.4	####	3,730	1,913
P3	8,946	97.8	2,228	3,468	1,929
P4	9,122	99.8	####	3,735	1,914
Q1	0	0.0	####	####	####
Q2	604	6.6	19,005	21,989	16,887
Q3	15	0.2	636	944	795
Q4	8,524	93.2	####	2,441	1,790

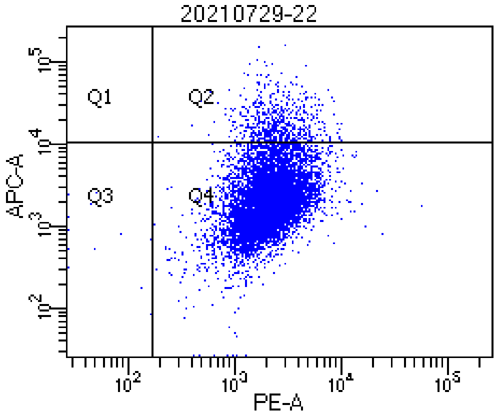

BD FACSDiva 8.0.2

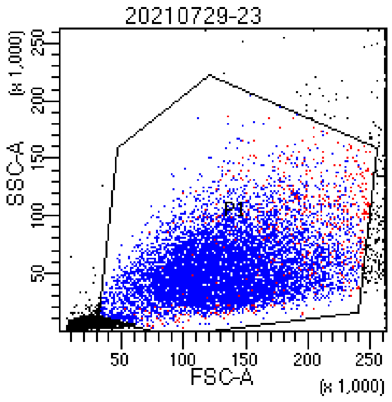

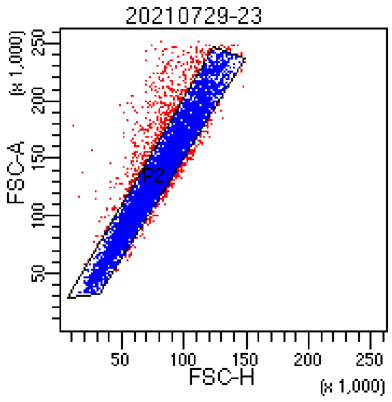

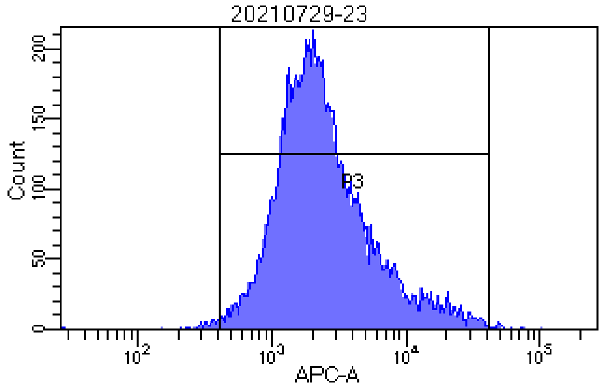

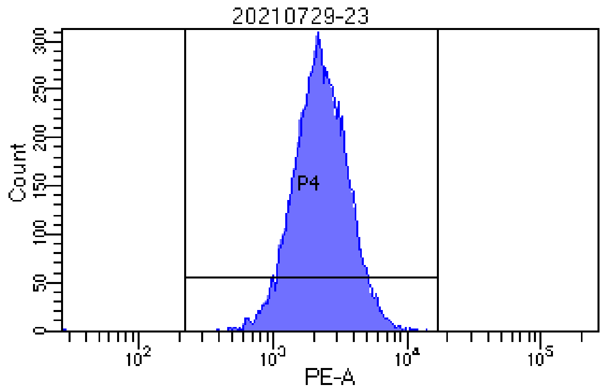

Experiment Name: YXQ-1
Specimen Name: 20210729
Tube Name: 23
Record Date: Jul 29, 2021 5:57:45 PM

Population	#Events	%Pare...	APC-A Geo M...	APC-A Mean	APC-A Median
P1	10,010	73.2	####	3,967	2,136
P2	9,114	91.0	####	3,880	2,069
P3	8,961	98.3	2,389	3,636	2,080
P4	9,105	99.9	####	3,883	2,070
Q1	0	0.0	####	####	####
Q2	639	7.0	19,004	21,647	17,290
Q3	6	0.1	508	723	674
Q4	8,469	92.9	####	2,542	1,953

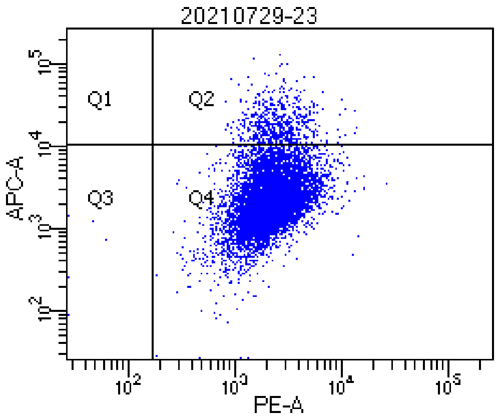

BD FACSDiva 8.0.2

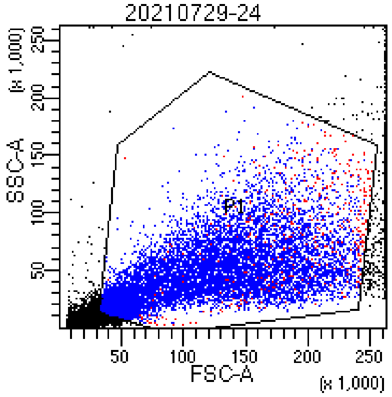

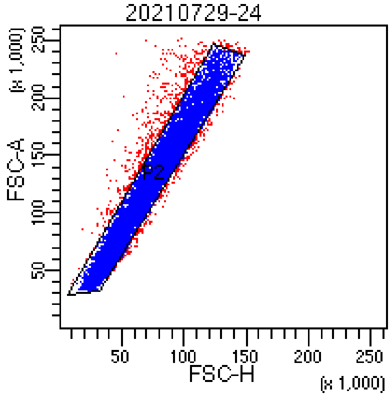

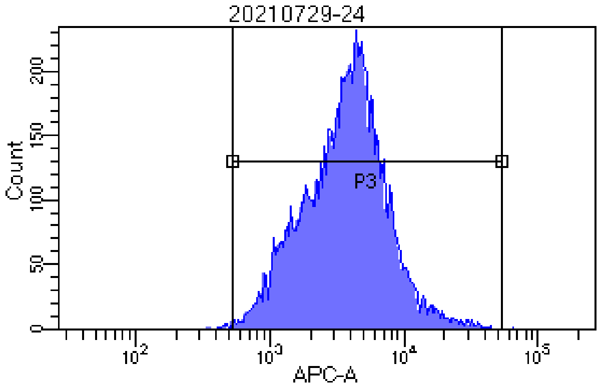

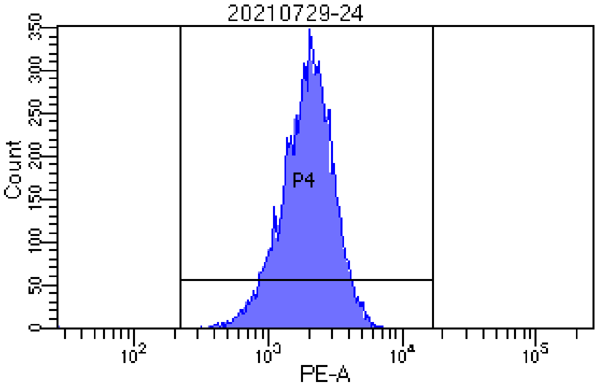

Experiment Name:	YXQ-1
Specimen Name:	20210729
Tube Name:	24
Record Date:	Jul 29, 2021 5:58:52 PM

Population	#Events	%Pare...	APC-A Geo M...	APC-A Mean	APC-A Median
P1	10,000	56.5	####	4,835	3,770
P2	9,164	91.6	####	4,684	3,651
P3	9,098	99.3	3,499	4,597	3,665
P4	9,149	99.8	####	4,690	3,659
Q1	0	0.0	####	####	####
Q2	538	5.9	16,910	19,177	14,884
Q3	10	0.1	758	862	885
Q4	8,616	94.0	####	3,784	3,487

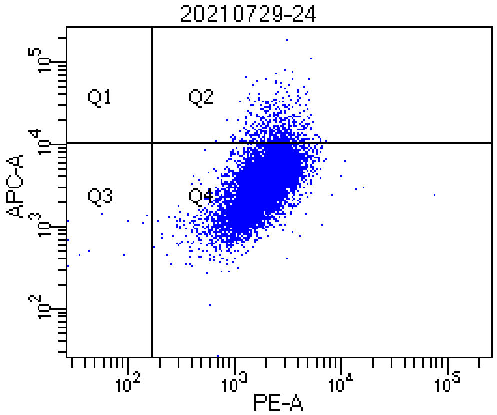

BD FACSDiva 8.0.2

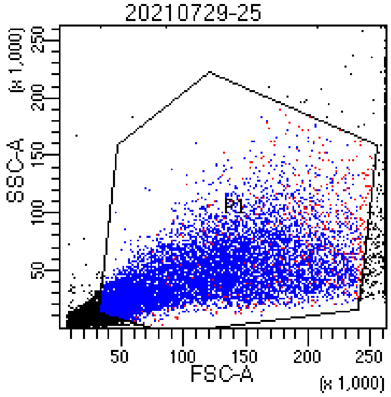

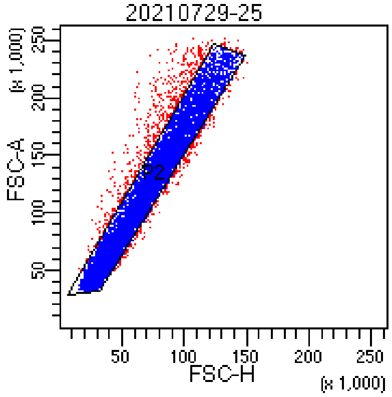

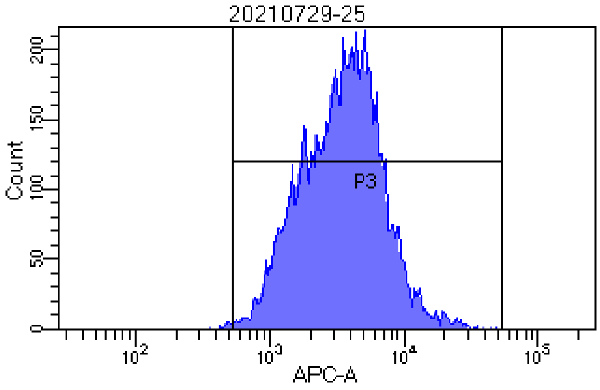

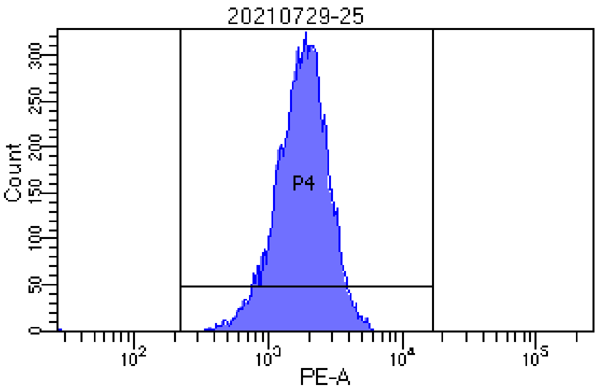

Experiment Name: YXQ-1
Specimen Name: 20210729
Tube Name: 25
Record Date: Jul 29, 2021 5:59:48 PM

Population	#Events	%Pare...	APC-A Geo M...	APC-A Mean	APC-A Median
P1	10,000	54.0	####	4,472	3,550
P2	9,194	91.9	####	4,269	3,405
P3	9,144	99.5	3,302	4,261	3,421
P4	9,172	99.8	3,283	4,276	3,410
Q1	0	0.0	####	####	####
Q2	408	4.4	16,176	17,684	14,324
Q3	15	0.2	835	983	897
Q4	8,771	95.4	####	3,650	3,290

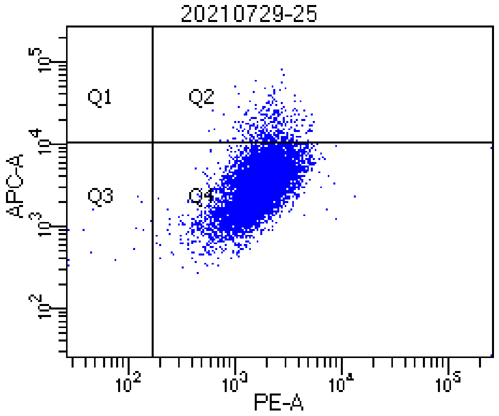

BD FACSDiva 8.0.2

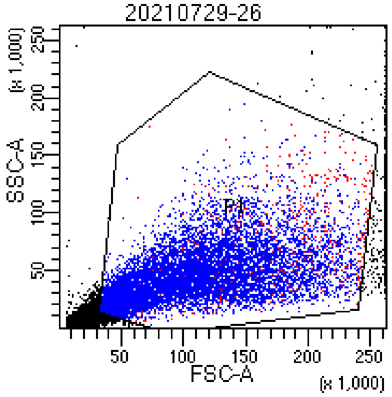

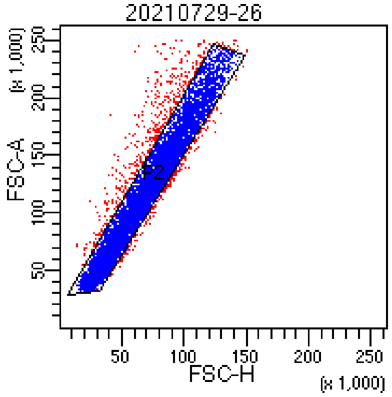

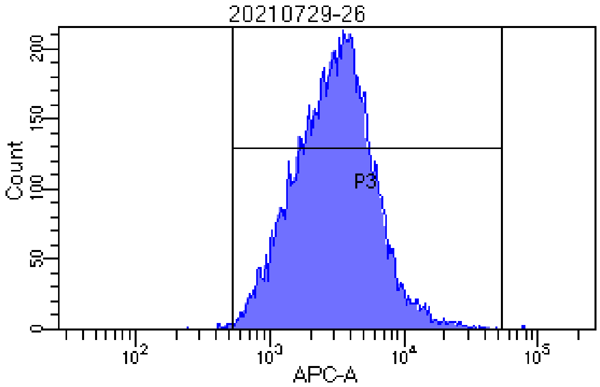

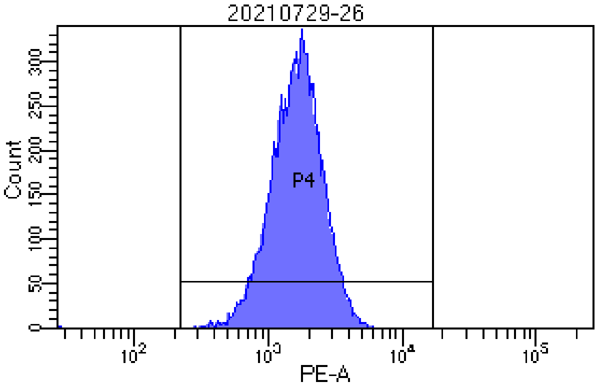

Experiment Name:	YXQ-1
Specimen Name:	20210729
Tube Name:	26
Record Date:	Jul 29, 2021 6:01:32 PM

Population	#Events	%Pare...	APC-A Geo M...	APC-A Mean	APC-A Median
P1	10,000	57.9	2,989	4,019	3,049
P2	9,265	92.7	2,893	3,867	2,942
P3	9,195	99.2	2,916	3,774	2,956
P4	9,242	99.8	2,902	3,874	2,948
Q1	0	0.0	####	####	####
Q2	345	3.7	17,351	20,148	14,674
Q3	18	0.2	761	1,042	751
Q4	8,902	96.1	2,706	3,242	2,854

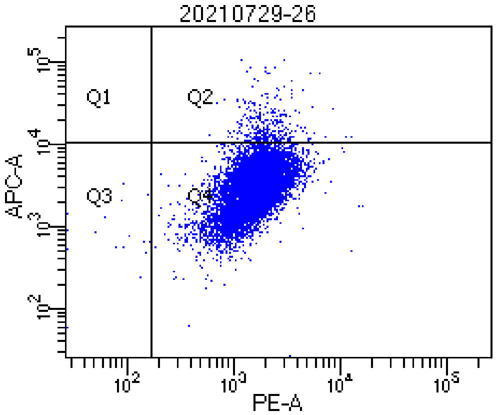

BD FACSDiva 8.0.2

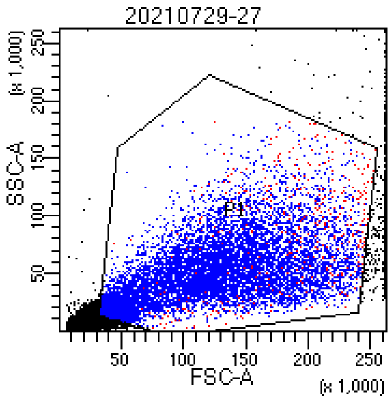

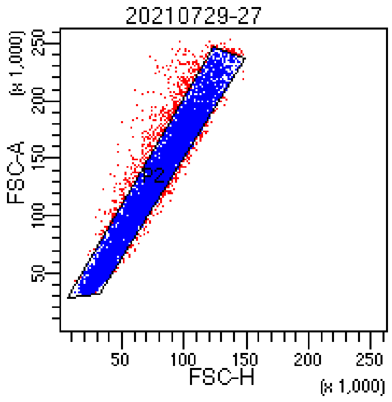

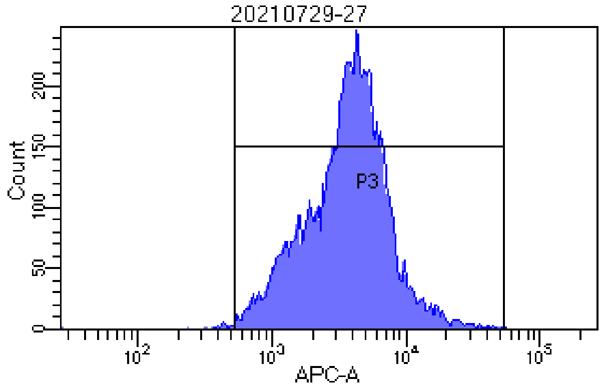

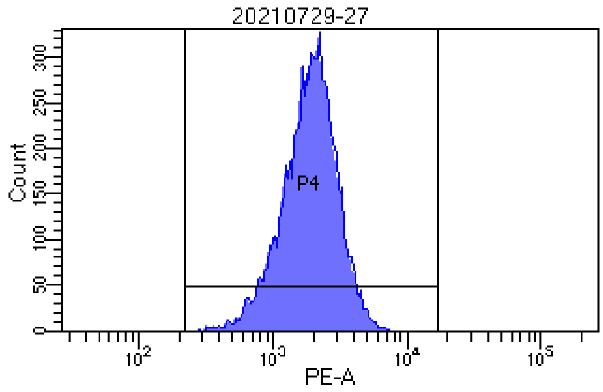

Experiment Name: YXQ-1
Specimen Name: 20210729
Tube Name: 27
Record Date: Jul 29, 2021 6:02:19 PM

Population	#Events	%Pare...	APC-A Geo M...	APC-A Mean	APC-A Median
P1	10,000	49.3	####	4,764	3,778
P2	9,237	92.4	####	4,575	3,651
P3	9,143	99.0	3,477	4,559	3,681
P4	9,220	99.8	####	4,582	3,657
Q1	0	0.0	####	####	####
Q2	507	5.5	16,937	18,848	15,000
Q3	8	0.1	927	1,073	876
Q4	8,722	94.4	####	3,749	3,509

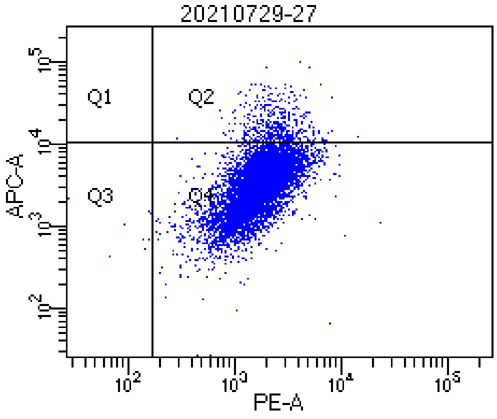

BD FACSDiva 8.0.2

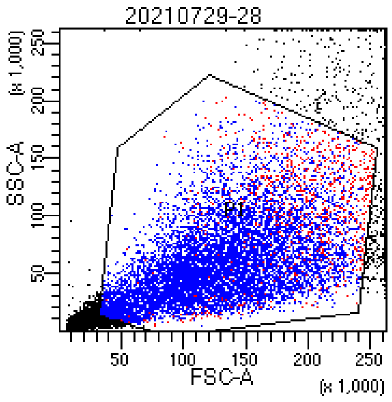

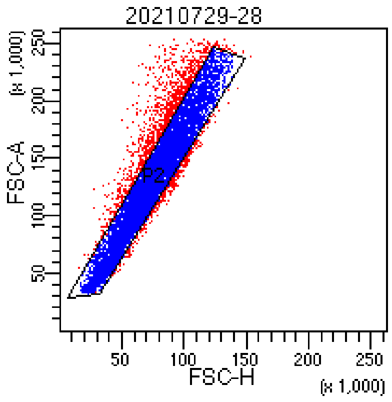

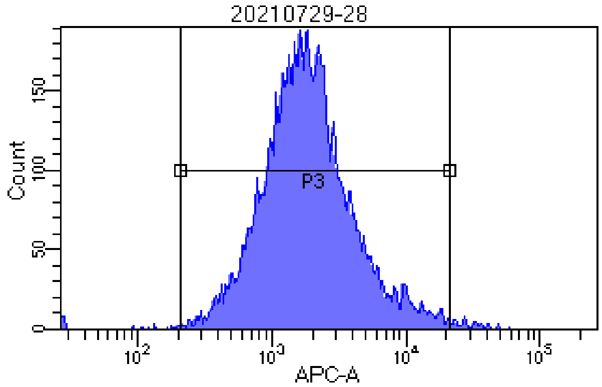

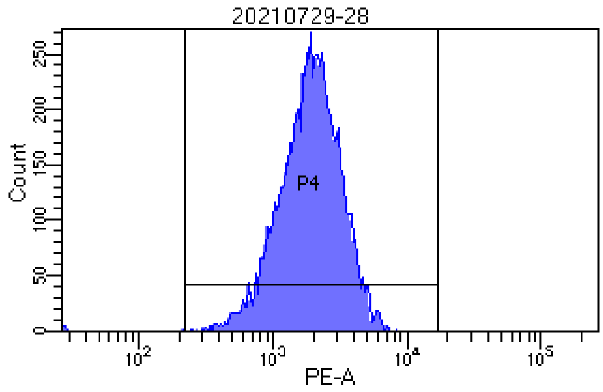

Experiment Name: YXQ-1
Specimen Name: 20210729
Tube Name: 28
Record Date: Jul 29, 2021 6:03:06 PM

Population	#Events	%Pare...	APC-A Geo M...	APC-A Mean	APC-A Median
P1	10,000	59.4	####	3,170	1,833
P2	8,567	85.7	####	2,919	1,703
P3	8,403	98.1	1,774	2,483	1,697
P4	8,540	99.7	####	2,925	1,708
Q1	0	0.0	####	####	####
Q2	331	3.9	18,748	22,155	16,450
Q3	17	0.2	372	451	421
Q4	8,219	95.9	####	2,149	1,654

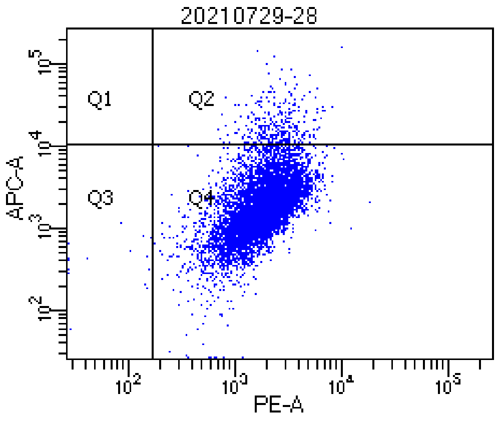

BD FACSDiva 8.0.2

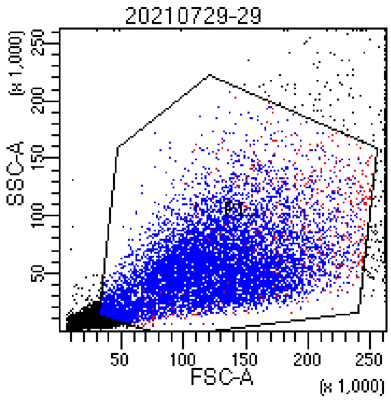

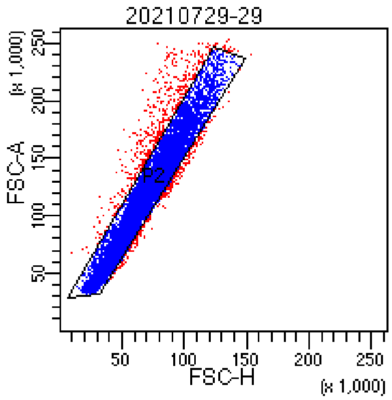

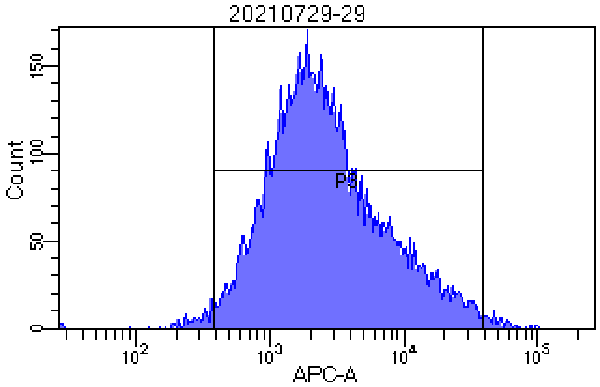

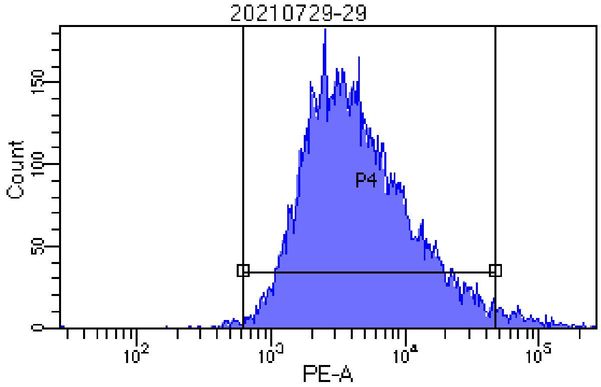

Experiment Name:	YXQ-1
Specimen Name:	20210729
Tube Name:	29
Record Date:	Jul 29, 2021 6:04:10 PM

Population	#Events	%Pare...	APC-A Geo M...	APC-A Mean	APC-A Median
P1	10,000	64.5	####	5,119	2,317
P2	9,173	91.7	####	4,971	2,253
P3	8,880	96.8	2,597	4,349	2,271
P4	8,799	95.9	####	4,967	2,250
Q1	0	0.0	####	####	####
Q2	1,015	11.1	19,698	23,179	17,342
Q3	10	0.1	655	847	549
Q4	8,148	88.8	####	2,708	1,983

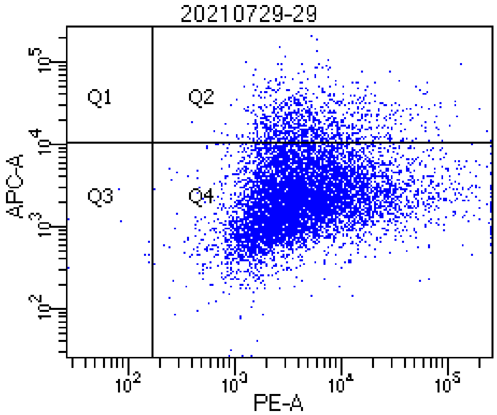

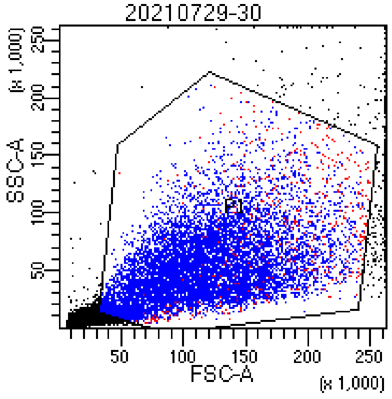

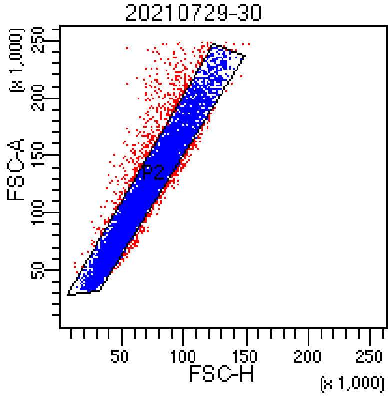

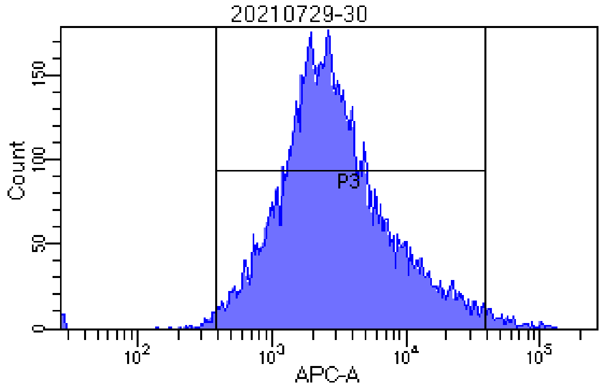

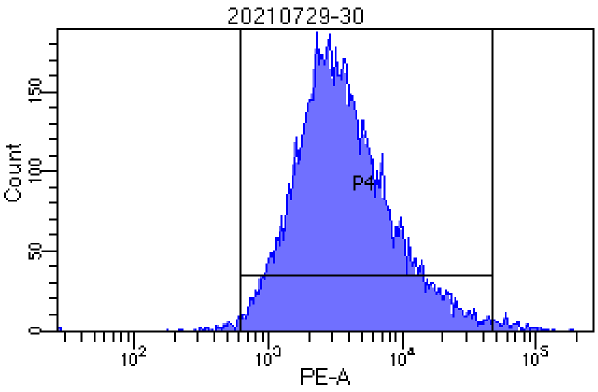

Experiment Name: YXQ-1
Specimen Name: 20210729
Tube Name: 30
Record Date: Jul 29, 2021 6:05:06 PM

Population	#Events	%Pare...	APC-A Geo M...	APC-A Mean	APC-A Median
P1	10,000	64.4	####	5,810	2,631
P2	9,126	91.3	####	5,630	2,553
P3	8,825	96.7	2,820	4,489	2,539
P4	8,852	97.0	####	5,649	2,562
Q1	1	0.0	27,709	27,709	27,709
Q2	1,041	11.4	21,564	26,731	18,767
Q3	12	0.1	598	2,101	1,408
Q4	8,072	88.5	####	2,912	2,282

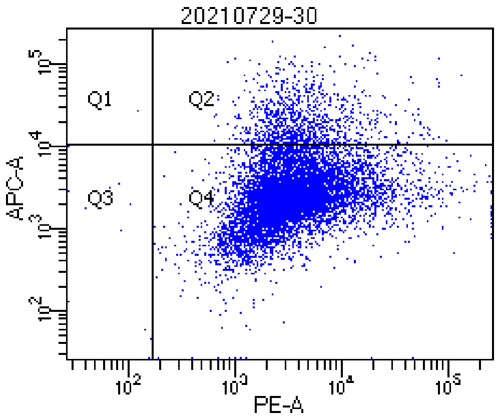

BD FACSDiva 8.0.2

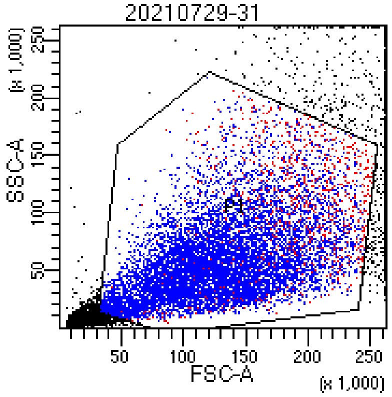

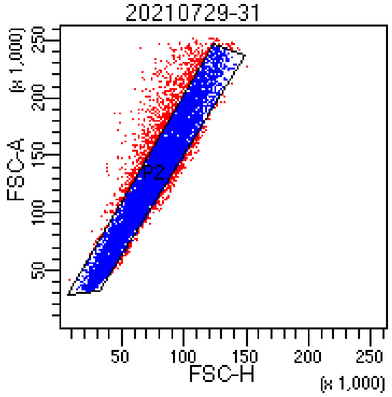

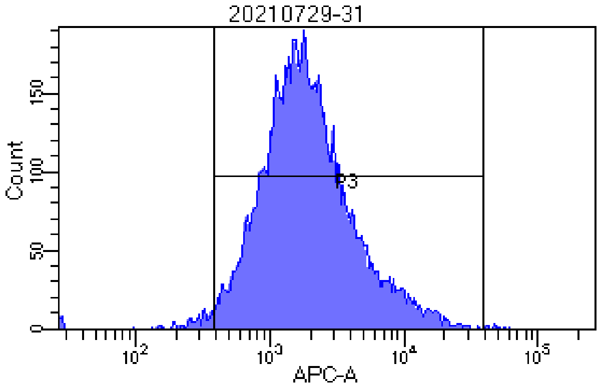

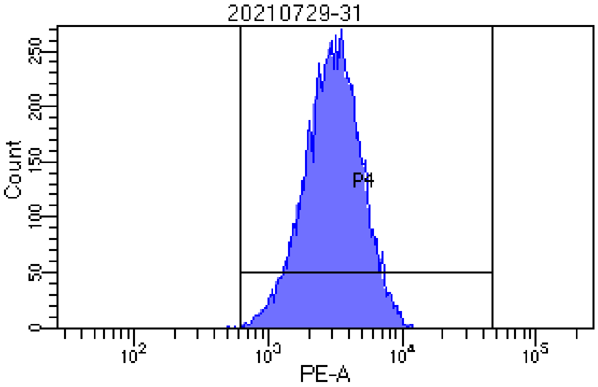

Experiment Name: YXQ-1
Specimen Name: 20210729
Tube Name: 31
Record Date: Jul 29, 2021 6:05:55 PM

Population	#Events	%Pare...	APC-A Geo M...	APC-A Mean	APC-A Median
P1	10,000	64.1	####	3,188	1,825
P2	8,598	86.0	####	2,943	1,700
P3	8,371	97.4	1,894	2,746	1,725
P4	8,560	99.6	####	2,953	1,704
Q1	0	0.0	####	####	####
Q2	334	3.9	18,063	21,275	15,504
Q3	1	0.0	355	355	355
Q4	8,263	96.1	####	2,202	1,649

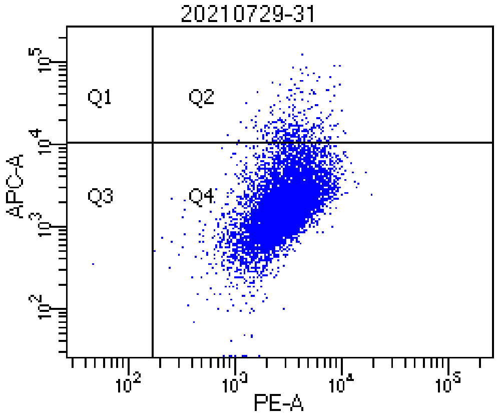

BD FACSDiva 8.0.2

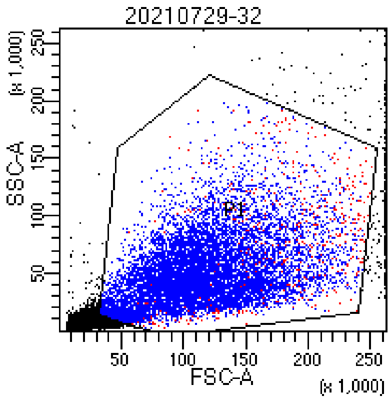

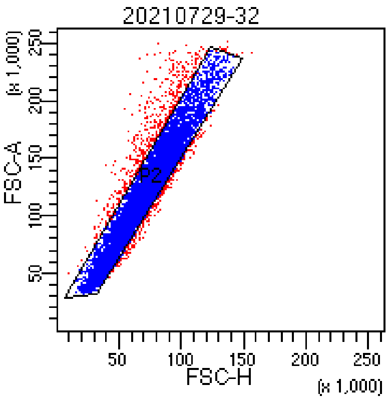

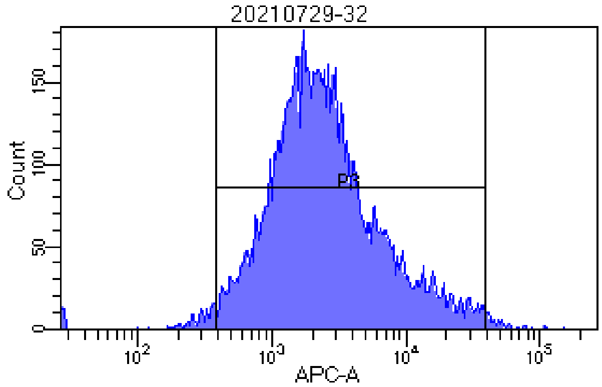

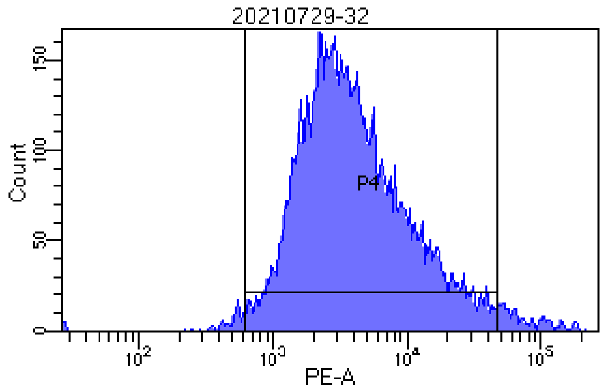

Experiment Name: YXQ-1
Specimen Name: 20210729
Tube Name: 32
Record Date: Jul 29, 2021 6:06:51 PM

Population	#Events	%Pare...	APC-A Geo M...	APC-A Mean	APC-A Median
P1	10,000	62.5	####	4,741	2,197
P2	9,173	91.7	####	4,648	2,147
P3	8,860	96.6	2,454	4,052	2,177
P4	8,683	94.7	####	4,680	2,154
Q1	0	0.0	####	####	####
Q2	847	9.2	20,832	24,911	18,279
Q3	11	0.1	321	568	407
Q4	8,315	90.6	####	2,590	1,951

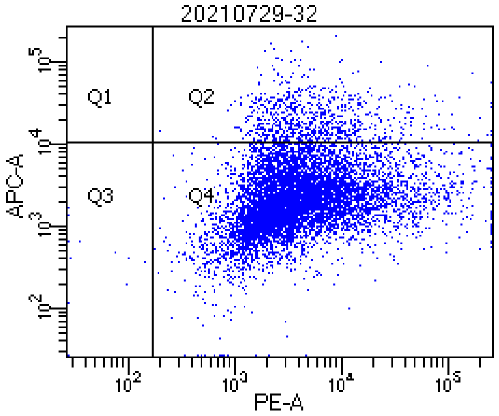

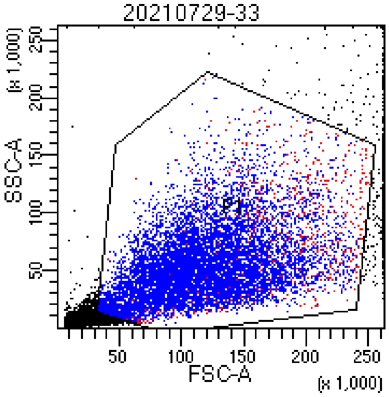

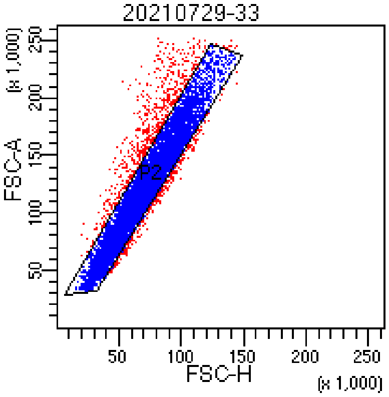

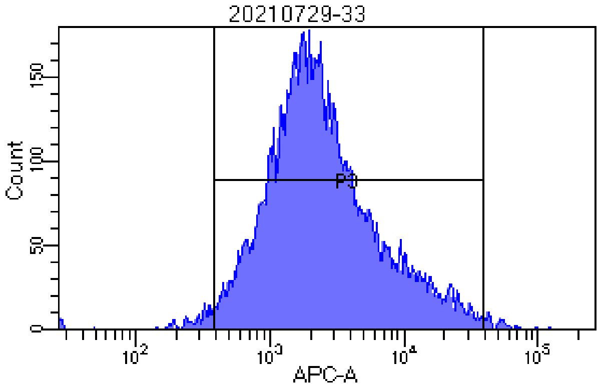

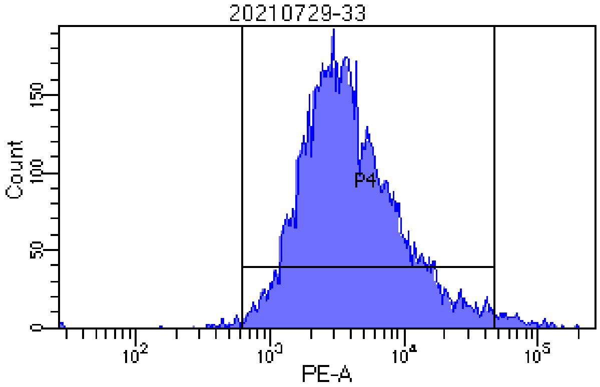

Experiment Name:	YXQ-1
Specimen Name:	20210729
Tube Name:	33
Record Date:	Jul 29, 2021 6:07:55 PM

Population	#Events	%Pare...	APC-A Geo M...	APC-A Mean	APC-A Median
P1	10,000	69.1	####	4,628	2,165
P2	9,067	90.7	####	4,498	2,091
P3	8,780	96.8	2,429	3,997	2,113
P4	8,720	96.2	####	4,553	2,096
Q1	1	0.0	12,227	12,227	12,227
Q2	864	9.5	19,546	22,838	17,482
Q3	15	0.2	####	1,023	723
Q4	8,187	90.3	####	2,567	1,902

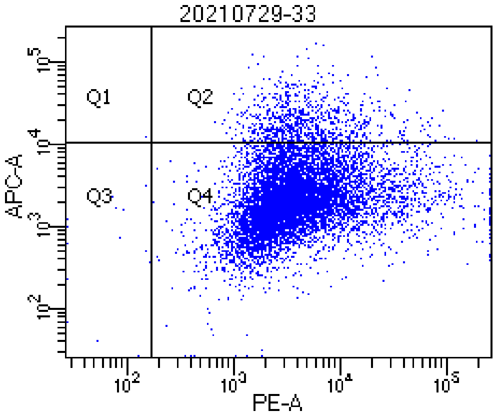

Supplement: Supplementary file 18 — Original Data File [file 41420_2022_968_MOESM18_ESM.pdf]
